# DNA-based platform for efficient and precisely targeted bioorthogonal catalysis in living systems

**DOI:** 10.1038/s41467-022-29167-x

**Published:** 2022-03-18

**Authors:** Yawen You, Qingqing Deng, Yibo Wang, Yanjuan Sang, Guangming Li, Fang Pu, Jinsong Ren, Xiaogang Qu

**Affiliations:** 1 State Key Laboratory of Rare Earth Resources Utilization and Laboratory of Chemical Biology, Changchun Institute of Applied Chemistry, Chinese Academy of Sciences, Changchun, 130022 P. R. China; 2 University of Science and Technology of China, Hefei, Anhui 230029 P. R. China; 3 Laboratory of Chemical Biology, Changchun Institute of Applied Chemistry, Chinese Academy of Sciences, Changchun, 130022 P. R. China

**Keywords:** Biotechnology, Biomaterials, Catalysis

## Abstract

As one of the typical bioorthogonal reactions, copper(I)-catalyzed azide-alkyne cycloaddition (CuAAC) reaction holds great potential in organic synthesis, bioconjugation, and surface functionalization. However, the toxicity of Cu(I), inefficient catalytic activity, and the lack of cell specific targeting of the existing catalysts hampered their practical applications in living systems. Herein, we design and construct a DNA-based platform as a biocompatible, highly efficient, and precisely targeted bioorthogonal nanocatalyst. The nanocatalyst presents excellent catalytic efficiency in vitro, which is one order of magnitude higher than the commonly used catalyst CuSO_4_/sodium ascorbate. The theoretical calculation further supports the contribution of DNA structure and its interaction with substrates to the superior catalytic activity. More importantly, the system can achieve efficient prodrug activation in cancer cells through cell type-specific recognition and produce a 40-fold enhancement of transformation compared to the non-targeting nanocatalyst, resulting in enhanced antitumor efficacy and reduced adverse effects. In vivo tumor therapy demonstrates the safety and efficacy of the system in mammals.

## Abstract (web summary)

Copper-click reaction has been used for a wide range of bio-conjugations but does suffer from toxicity issues. Here, the authors report on the growth of copper nanoparticles on DNA with linked aptamer targeting and demonstrate high catalytic effect and improved application due to targeting and biocompatibility.

## Introduction

Bioorthogonal reactions are emerging as promising approaches for intracellular labeling bio-targets and modulating biological processes without disturbing other biomolecules and function^1–11^. As one of the typical bioorthogonal reactions, CuAAC reaction has been applied as a powerful synthetic tool for bioconjugations or coupling functional groups and molecules with fast kinetics and high yields under mild conditions^12–15^. Despite great advance in organic synthesis, material science, and medicinal chemistry^16–18^, the implementation of CuAAC in living systems remains in its infancy. The inherent toxicity of the active catalytic species Cu(I) can damage normal cells and healthy tissues and cause numerous side effects, limiting the application of CuAAC within biological systems^19^. To eliminate the toxic effect of Cu(I), several approaches were explored to develop biocompatible catalysts, including utilization of ligand-chelated or support-stabilized Cu(I), in-situ reduction of Cu(II) salts with the addition of sodium ascorbate, metallic copper nanoparticles and nanostructured copper oxide^20–25^. However, these approaches still suffer from intrinsic drawbacks, which hamper their biological applications. First, the reaction time is rather long in general, and the yields are much insufficient. In some cases, high temperature, microwave or non-eco-friendly reagents was employed to enhance the reaction rate^26^, which cannot fulfill the requirements of CuAAC in living cells. Second, these catalysts themselves cannot recognize target cells^27^, limiting the applicable dosage to specific regions, compromising the catalytic transformation efficacy, even damaging normal cell populations, and causing numerous side effects due to nonspecific distribution. Recently, metal-organic frameworks supported heterogeneous copper nanocatalyst has been reported to catalyze CuAAC in mitochondria by decorating a triphenylphosphonium (TPP) group^28^. Nevertheless, the nanocatalyst showed low tumor selectivity and unsatisfactory treatment specificity since overcoming the barriers of extracellular matrix and cell membrane to selectively target specific cells is the premise of the subcellular organelle-targeted delivery^29^. Besides, complicated and tedious preparation processes, and difficulty in control of size, morphology, dispersion and stability which determine catalysis performance, are matters of concern.

DNA as a powerful and intelligent building block for construction of functional nanomaterials has attracted great attention^30–32^. Aside from excellent properties, including biocompatibility, programmability, and easy synthesis and modification, DNA can be used as a template to synthesize metal nanoclusters or nanoparticles, such as Au, Ag, and Cu^33,34^, whose size can be exquisitely controlled by the length and sequence of DNA^35^. These DNA-templated nanoparticles have been used in many areas, such as nanoelectronics, sensing, and bioimaging^36^. Meanwhile, aptamers, single-stranded DNA or RNA sequences selected through SELEX technology to recognize various targets with high affinity and specificity, have been widely utilized for analytical chemistry and nanomedicines^37–39^. Aptamer conjugated on the surface of nanoparticles can specifically bind to overexpressed receptors on tumor cells, and then improve cellular uptake through the receptor-mediated endocytosis^40^. In the meantime, DNA strands were recently exploited to regulate catalytic activity of nanozymes^41,42^. For example, DNA could enhance activity of Fe_3_O_4_ for the peroxidation of 3,3′,5,5′-tetramethylbenzidine by 10-fold^43^. The enhanced reactivity could be attributed to highly effective molarity of reaction components in or near the DNA facilitated by electrostatic attraction, hydrogen bonding and aromatic stacking^43^. These properties open the opportunities for designing DNA-based platform as biocompatible, highly active, and targeted bioorthogonal catalyst.

Inspired by these unique properties, we construct a DNA-based platform for efficient and precisely targeted bioorthogonal catalysis in living systems. The introduction of DNA offers an attractive catalyst synthesis method. The platform can catalyze azide-alkyne cycloaddition reaction in vitro with short reaction time and high yield. The theoretical calculation further supports the contribution of DNA to the enhanced catalytic activity. More importantly, the work addresses the issues of the lack of precise targeting and inefficient catalytic activity of the bioorthogonal catalyst in living system. The highly efficient, targeted, and biocompatible nanocatalyst can be applied in tumor therapy via in situ activation of prodrugs. The precise and efficient catalytic synthesis of drugs in tumor tissues can ensure satisfactory and specific antitumor efficacy and reduce the serious adverse effects. Furthermore, the programmability of nucleic acids enables the generality of DNA-based nanocatalyst by simply using different sequences. The work provides exciting opportunities to design versatile bioorthogonal catalysts for in vivo applications in complex multicellular organisms.

## Results and discussion

### The design and preparation of DNA-based copper nanoparticles

**Fig. 1:**
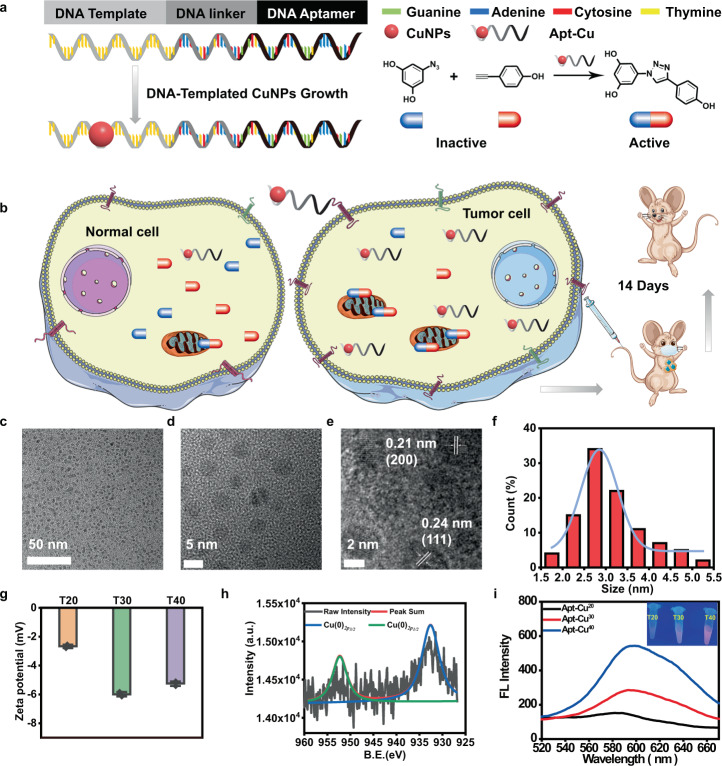
Design and characterization of DNA-based bioorthogonal catalyst. **a** Illustration of the design and synthesis of DNA-templated CuNPs as bioorthogonal catalyst. **b** Aptamer-mediated CuNPs for cell-specific identification and targeted cancer therapy in vivo; (**c**) Transmission electron microscopy (TEM) image of Apt-Cu^30^. **d**, **e** High-resolution TEM images of Apt-Cu^30^. **f** Histogram of size distribution of Apt-Cu^30^. The total number of nanoparticles counted was 100. **g** Zeta-potentials of Apt-Cu^20^, Apt-Cu^30^, and Apt-Cu^40^ in H_2_O (pH 6.6). Data were presented as mean ± SD (*n* = 3 independent experiments). **h** XPS spectra of Apt-Cu^30^ (red line: fitting curve). **i** Fluorescence spectra of Apt-Cu^20^, Apt-Cu^30^ and Apt-Cu^40^ excited at 340 nm. (Inset) Photographs of Apt-Cu under UV lamp. Source data are provided as a Source Data file.

The design principle of the DNA-based nanocatalyst is illustrated in Fig. 1a. The single-stranded DNA is composed of three domains. One portion is a thymine-rich sequence which can serve as a template for the formation of copper nanoparticles (CuNPs) with small sizes, good dispersion, stability, and low toxicity to cells. Since the size of the nanocatalyst is of utmost importance in catalysis for providing a large surface-to-volume ratio and increased number of defect sites^44,45^, CuNPs with different sizes and fluorescence intensities can be obtained by changing the number of thymine. Herein, DNA strands containing different lengths of thymine-rich regions (T20, T30, and T40) are chosen. The second portion is aptamer which can target cancer cells. Mucin 1 (MUC1), a cell-surface receptor protein, is known to be uniquely and abundantly expressed on the surface of adenocarcinoma cells rather than on normal cells^46^. MUC1 aptamer is selected for targeting MUC1 protein on the surface of cancer cells (Fig. 1b). Besides, AS1411 aptamer possessing high affinity to nucleolin overexpressed in tumor cells is also selected^47^. The use of different aptamers targeting different types of cancer cells allows for the versatile applicability. The third portion is a 15-base strand which links the different lengths of thymine-rich regions and different types of aptamers. The resulting nanocomposites are termed as Apt-Cu^20^, Apt-Cu^30^, and Apt-Cu^40^ and can be used for bioorthogonal catalysis.

First, DNA-templated CuNPs were synthesized according to the previously reported method. The copper content of the DNA-templated CuNPs after synthesis was shown in Supplementary Table 2. The obtained DNA-templated CuNPs were monodispersed (Fig. 1c–e). The average sizes of CuNPs were 1.78, 3.12, and 4.79 nm for DNA strands containing T20, T30, and T40, respectively (Supplementary Figs. 1–3, and Fig. 1f). Atomic force microscope (AFM) images showed one nanoparticle formed on one DNA template (Supplementary Fig. 4). The zeta potential measurement showed that the nanocomposites were negatively charged (Fig. 1g). X-ray photoelectron spectroscopy (XPS) spectrum of Cu*2p* showed two distinct peaks at 932.6 eV (Cu *2p*_3/2_), and 952.2 eV (Cu *2p*_1/2_), corresponding to Cu (0) nanoparticles (Fig. 1h). Then the fluorescence spectra of CuNPs were investigated. As shown in Fig. 1i, CuNPs presented fluorescence emissions at 600 nm upon excitation at 340 nm. The intensities increased significantly with the increase of thymine numbers. Under the irradiation of UV lamp, the nanocomposites exhibited red fluorescence. The results indicated the successful synthesis of DNA-templated CuNPs.

### Catalytic performance of the DNA-based nanocatalysts in vial

**Fig. 2:**
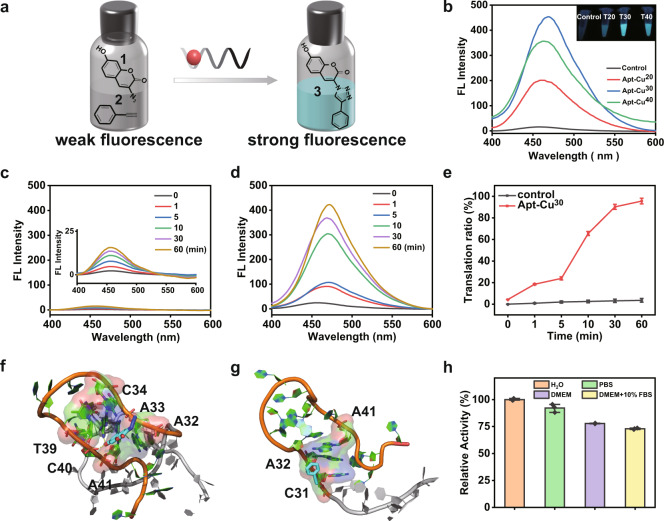
Characterization of CuAAC reaction in vial. **a** Scheme of CuAAC reaction catalyzed by Apt-Cu in vial. **b** Fluorescence spectra of the CuAAC reaction catalyzed by Apt-Cu (with an equivalent amount of copper 5 µM). (Inset) Fluorescence photogragh of precursors **1** and **2** without and with Apt-Cu. **c**, **d** Fluorescence spectra of CuAAC reaction without and with Apt-Cu^30^ at different reaction time, respectively. **e** Translation ratio of precursors **1** and **2** in the absence and presence of Apt-Cu^30^. Data were presented as mean ± SD (*n* = 3 independent experiments). **f**, **g** The binding mode of precursors **1** and **2** with DNA by molecular docking, respectively. **h** The relative catalytic capability of the nanocatalyst under different physiological conditions, including in phosphate-buffered saline (PBS), cell culture medium (DMEM) without or with 10% fetal bovine serum (FBS), respectively. Data were presented as mean ± SD (*n* = 3 independent experiments). Source data are provided as a Source Data file.

The catalytic capability of the DNA-based nanocatalysts was tested by catalyzing the CuAAC reaction in vitro. Transformation of precursors **1** (3-azido-7-hydroxycoumarin) and **2** (phenylacetylene) into triazole **3** in the presence of catalyst was chosen as a model reaction (Fig. 2a). **1** and **2** are non-fluorescent (Supplementary Fig. 5), while the product **3** emits strong fluorescence (Fig. 2b, Supplementary Fig. 6) (λ_ex_ = 340 nm; λ_em_ = 460 nm). In the experiment, Apt-Cu^20^, Apt-Cu^30^ and Apt-Cu^40^ were incubated with the mixture of **1** and **2** in vials, respectively. The samples instantaneously showed cyan-blue fluorescence, suggesting the superior catalytic activity of these CuNPs. The products were measured using fluorescence spectra. As shown in Fig. 2b, all three Apt-Cu nanocatalysts could induce increase of fluorescence intensity at 460 nm upon excitation at 340 nm, implying the formation of triazole **3**. Moreover, the fluorescence intensities increased in the order of Apt-Cu^30^ > Apt-Cu^40^ > Apt-Cu^20^. Subsequently, to investigate the catalytic efficiency and kinetic process, the changes of fluorescence intensity at 460 nm with time were recorded. As shown in Fig. 2c, in the absence of nanocatalyst, the fluorescence remained constant over the period. Upon addition of nanocatalyst, the fluorescence turned up instantaneously. Within the first 10 min, a rapid increase in fluorescence was clearly observed (Fig. 2d, e), suggesting the excellent catalytic activity. The reaction rates increased in the order of Apt-Cu^30^ > Apt-Cu^40^ > Apt-Cu^20^ (Supplementary Fig. 7). The conversion rates of the three Apt-CuNPs at 10 min were analyzed by high-performance liquid chromatography (HPLC), respectively (Supplementary Fig. 8). The results were consistent with the fluorescent results. The fluorescence intensity reached the plateau after 60 min, indicating the catalytic reaction was nearly completed. Furthermore, the conversion rates of Apt-Cu^30^ at different time points were analyzed by HPLC. As shown in Supplementary Figs. 9–11, in the presence of Apt-Cu^30^, the conversion rate increased with time. 90% of the substrate was converted into the product within 60 min, supporting the result of the fluorescence measurement. Meanwhile, the commonly used catalyst CuSO_4_/sodium ascorbate was tested for comparison. As shown in Supplementary Table 3, the catalytic transformation using Apt-Cu^30^ was 26 times higher than that of using CuSO_4_/sodium ascorbate in 0.5 h. Besides, CuNPs synthesized using DNA strand without aptamer region (WA-Cu^30^) were used for comparison. Both Apt-Cu^30^ and WA-Cu^30^ could catalyze CuAAC reaction in H_2_O, and there was no obvious difference of fluorescence intensity, indicating aptamer did not influence the catalytic efficiency of CuNPs in vitro (Supplementary Fig. 12). Moreover, DNA strand without thymine-rich region was tested as control. Due to the lack of thymine-rich sequence as a template, CuNPs could not be synthesized. The fluorescence spectra of click reaction showed weak signal (Supplementary Fig. 13), indicating the catalytic capability of this DNA strand was low even in a long reaction time (Supplementary Table 4).

Sizes, supporting materials and surface sites of nanocatalysts have major impacts on their performance. Generally, the smaller size possesses the larger specific surface area, leading to the higher catalytic activity and the higher atom utilization rate. Herein, the size of CuNPs obtained increased with the length of DNA templates. However, their catalytic activities did not completely follow the size-activity relationship, and Apt-Cu^20^ presented the poorest activity. Therefore, it was deemed important to explore other factors. It is reported that more poly T bases could provide more stability for CuNPs^48^. Apt-Cu^20^ possessed the worst stability due to the shortest DNA template, which might result in the lower catalytic activity. Cu is easily oxidized due to its low reduction potential. XPS analysis was performed to determine the oxidation state of Apt-Cu^20^. As shown in Supplementary Fig. 14, two peaks at 932.4 and 952.2 eV assigned to Cu (0) were observed. Meanwhile, there were two peaks at 933.6 eV (Cu *2p*_*3/2*_) and 953.6 eV (Cu 2*p*_*1/2*_) identical to Cu (II), indicating the presence of Cu (II). The content of Cu (0) accounts for only 38.8%. The reduced content of Cu (0) and the existing oxidation state on the surface of Apt-Cu^20^ would compromise its catalytic activity. To the end, the synergic effects of size, supporting materials and surface sites determined the catalytic efficiency of these CuNPs.

In vitro, aptamer did not present specific recognition because there are no cells. However, the nanocatalysts still showed higher catalytic activity. To further clarify the role of DNA in the highly efficient catalysis, molecular dynamics simulation was used to explore the structure of DNA and their interaction with substrates^49,50^. The DNA structure (T30-15 base linker) was first relaxed by molecular dynamics simulation. The root-mean-square deviation (RMSD) values during the simulation indicated that the DNA sequence can reach a stable state in 20 ns (Supplementary Fig. 15). During the 30 ns simulation, the 15-base motif (linker sequence) can quickly fold to a hairpin-like secondary structure. In contrast, the T30 motif did not form any stable secondary structure. The last frame of simulation was employed for docking with **1** and **2**. As illustrated in Fig. 2f, the hydroxyl group of **1** inserted in the hairpin-like structure and generated two hydrogen bonds with A33 and C40. What’s more, the N_3_ group exposed to the bulk water, which would not affect the combination with **2**. Meanwhile, the hydrophobic interactions between **1** and nucleic acids A32, C34, T39 and A41 stabilized the complex. For **2**, the complex was stabilized only by hydrophobic interactions between the ligand and the nucleic acids C31, A32 and A41 (Fig. 2g). The interactions between DNA and substrates indicated high molarity of reaction components in or near the DNA and proximity to the catalytic sites, resulting in more efficient catalytic activity.

We also tested the catalytic capability of these nanocatalysts under different physiological conditions (Fig. 2h, Supplementary Fig. 16). Similar results were obtained in these buffers, suggesting the nanocatalyst could be used in biological systems without loss of catalytic activity.

### Cell targeting ability and intracellular catalysis of the nanocatalyst

**Fig. 3:**
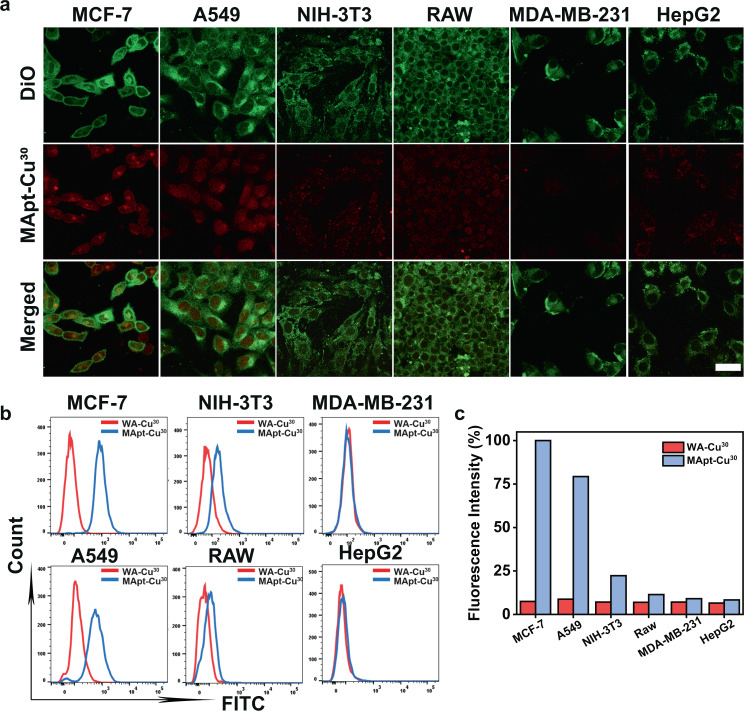
Specific recognition of target cells by MApt-Cu^30^ and WA-Cu^30^. **a** Confocal microscopy images of MCF-7, A549, NIH-3T3, RAW, MDA-MB-231, and HepG2 cells treated with MApt-Cu^30^ (copper 5 µM); Images are representative of three independent biological samples. scale bars: 50 µm. **b** Flow cytometry assay of different cells treated with MApt-Cu^30^ and WA-Cu^30^ (copper 5 µM), respectively. **c** Quantitative analysis of fluorescence intensity of MApt-Cu^30^ and WA-Cu^30^ internalized into different cells by flow cytometry. In the section of Fig. 3b, c FAM-labeled MUC1 aptamer was used to prepare MApt-Cu^30^ for fluorescence assay. Source data are provided as a Source Data file.

After demonstrating the catalytic activity of Apt-Cu in vial, we next tested their targeting capability. Due to the optimal catalytic activity of T30-templated CuNPs among the three CuNPs, MUCI aptamer-T30-templated-CuNPs (MApt-Cu^30^) were used for further research. CuNPs synthesized using DNA strand without aptamer region (WA-Cu^30^) were used as a control. First, the cytotoxicity of MApt-Cu^30^ to different living cells was measured. 3-(4, 5-dimethyl-2-yl)-2, 5-diphenyltetrazolium bromide (MTT) experiments indicated that MApt-Cu^30^ was biocompatible even at the concentration of 50 μM (Supplementary Fig. 17). For targeting research, MCF-7 cells (human breast cancer cells), a MUC1-overexpressing cancer cell line, were used as a model. The cellular uptake of MApt-Cu^30^ and WA-Cu^30^ was monitored and quantified by confocal microscopy and inductively coupled plasma mass spectrometry (ICP-MS) analyzing via tracking Cu, respectively. Confocal microscopy images showed the cells incubated with MApt-Cu^30^ presented bright red fluorescence in the cytoplasm within 6 h (Supplementary Figs. 18, 19), while the cells incubated with WA-Cu^30^ showed weak red fluorescence even for 30 h (Supplementary Figs. 20, 21). The ICP-MS results confirmed the maximum cellular internalization efficacy of MApt-Cu^30^ could be reached in 4 h (Supplementary Fig. 22). The result confirmed that MApt-Cu^30^ nanocatalyst could specifically bind to MUC1 protein on the surface of MCF-7 cells, resulting in enhanced endocytosis. Furthermore, the ability of MApt-Cu^30^ to recognize different types of cancer cells was explored using MUC1-positive (MUC1^+^) cancer cell lines, A549 (human lung cancer cell) and MCF-7, MUC1-negative (MUC1^-^) cancer cell lines, HepG2 (human hepatoma cell) and MDA-MB-231 (human breast cancer cell), and the normal cells NIH 3T3 (rat fibroblast cell), RAW 264.7 (murine macrophage cell) and HEK293 (human embryonic kidney cell). As shown in Fig. 3a, the cytomembranes were stained with DiO dye and emitted green fluorescence. MUC1^+^ cells incubated with MApt-Cu^30^ presented strong red emission inside the green cytomembranes, while MUC1^−^ cells and normal cells exhibited weak red emission, indicating MApt-Cu^30^ could differentiate the specific type of cancer cells and normal cells. Furthermore, the flow cytometry measurement showed that MCF-7 and A549 cells treated with MApt-Cu^30^ had visible right shift (Fig. 3b, Supplementary Fig. 23a) and high fluorescence intensity ratio (Fig. 3c) in comparison with MCF-7 and A549 cells treated with WA-Cu^30^. Slight or no shift and fluorescence change was observed for normal cells or MUC1^−^ cancer cells under identical conditions, confirming the target recognition of MApt-Cu^30^ for type-specific cells. In addition, we tested the targeting capability of a single DNA sequence MApt and WA without growth of CuNPs. The flow cytometry analysis results proved that the target recognition capability was not influenced after the synthesis of CuNPs (Supplementary Figs. 23b, 24, 25).

**Fig. 4:**
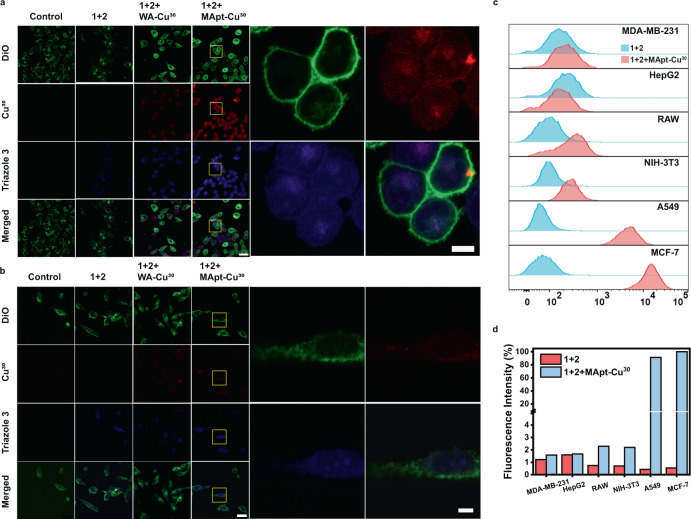
CuAAC reaction catalyzed by MApt-Cu^30^ in specific-type cells. **a**, **b** Confocal microscopy images of MCF-7 and MDA-MB-231 cells treated with **1** + **2**, **1** + **2** + WA-Cu^30^, and **1** + **2** + MApt-Cu^30^, respectively. Images are representative of three independent biological samples. Scale bars, 50 μm (main images), 10 μm (High-resolution images of corresponding cells treated with **1** + **2** + MApt-Cu^30^). **c** Flow cytometry assays of different cells treated with **1** + **2**, and **1** + **2** + MApt-Cu^30^. **d** Quantitative analysis of fluorescence intensity of different cells treated with **1** + **2**, and **1** + **2** + MApt-Cu^30^ by flow cytometry. Source data are provided as a Source Data file.

Subsequently, the ability of MApt-Cu^30^ to target cancer cells to perform catalysis was determined by monitoring the signals of cell membrane dye-DiO (green), CuNPs (red) and the product triazole **3** (blue) using confocal microscopy. As shown in Fig. 4a, MCF-7 cells treated with precursors+WA-Cu^30^ presented weak red fluorescence, while cells treated with precursors+MApt-Cu^30^ exhibited strong red fluorescence. It indicated that aptamer could increase CuNPs uptake via receptor-mediated endocytosis. MCF-7 cells treated with precursors or precursors+WA-Cu^30^ displayed weak blue fluorescence, while cells treated with precursors+MApt-Cu^30^ showed intense blue fluorescence. It implied that higher transformation efficiency of precursors **1** and **2** into triazole **3** in the presence of MApt-Cu^30^ was achieved, which could be attributed to the higher accumulation of CuNPs as catalyst in the living cell. The merged fluorescence image of CuNPs and triazole **3** revealed that the CuAAC reaction was efficiently achieved within the MUC1^+^ cancer cells. The LC-MS analysis result also demonstrated the synthesis of triazole **3** in MCF-7 cells (Supplementary Fig. 26). Similar results were obtained for A549 cells (Supplementary Fig. 27). For MDA-MB-231 cells treated with precursors+WA-Cu^30^ and precursors+MApt-Cu^30^, they presented weak red fluorescence and there was no obvious difference in red fluorescence (Fig. 4b). It indicated that MUC1 aptamer could not recognize MUC1^−^ cells without related receptors to enhance CuNPs uptake, leading to the lower amount of CuNPs in these cells. Consequently, these cells presented low blue fluorescence due to the low yield of triazole **3** catalyzed by a limited amount of CuNPs. Similar phenomena were observed for HepG2, RAW 264.7, NIH-3T3 and HEK293 (Supplementary Figs. 28–31). Furthermore, the flow cytometric analysis showed that MApt-Cu^30^ caused above 180-fold increase of blue fluorescence in MUC1^+^ cells incubated with precursors **1** and **2** compared with the cells without MApt-Cu^30^ (Fig. 4c, d). In sharp contrast, WA-Cu^30^ presented rather low catalytic activity since the DNA strand could not specifically recognize these cells. Above 40-fold fluorescence enhancement was obtained for MApt-Cu^30^ catalyzed CuAAC reaction compared that with WA-Cu^30^. The above results confirmed that the higher catalytic yield of triazole **3** could be attributed to aptamer-mediated cell selectivity and enhanced uptake of CuNP catalyst.

To demonstrate the DNA-based CuNP catalyst as a universal platform for aptamer-mediated cell selectivity, AS1411 aptamer which can specifically bind to nucleolin overexpressed on human cervical cancer (HeLa) cells was selected as another model. First, the cytotoxicity of AS1411 aptamer-T30-templated-CuNPs (AApt-Cu^30^) was tested through MTT experiment. As shown in Supplementary Fig. 32, AApt-Cu^30^ was biocompatible to HeLa cells even at the concentration of 50 μM. Next, the cellular internalization and intracellular catalysis performance of AApt-Cu^30^ was monitored by confocal microscopy images (Supplementary Figs. 33, 34). HeLa cells treated with AApt-Cu^30^ presented stronger green, red and blue fluorescence compared with cells treated with WA-Cu^30^. The overlap of green fluorescence from FAM fluorescein labeled on AApt-Cu^30^, red fluorescence of AApt-Cu^30^, and the blue fluorescence of the product triazole **3** indicated that AApt-Cu^30^ was specifically internalized by HeLa cells and exhibited good intracellular catalytic activity. The results confirmed the universality of the DNA-based platform by simply using different aptamers.

### Targeted prodrug activation in living cells

Prodrug activations by bioorthogonal reactions have been considered as a powerful strategy for tumor inhibition with satisfactory effectiveness^51–54^. Having demonstrated the superior cell-specific recognition of MApt-Cu^30^ and the resulting excellent catalytic effect in CuAAC reaction, we next explored the application of the nanocatalyst in cancer therapy using Resveratrol (3,5,4′-trihydroxystilbene; Rsv) analog **6** as model drug^55^. Rsv and its analogs have elicited a vast interest due to its multiple beneficial properties, such as chemopreventive and antitumor activities^56^. However, the compounds can target many biomolecules and have different effect in different environments. The in-situ generation of Rsv analogues **6** could improve its anti-tumor effect and minimize the side effects. We synthesized the prodrug **4**, **5** and then utilized a MTT assay to investigate the cytotoxicity of **4**, **5**, and the resulting Rsv analogues **6**.

**Fig. 5:**
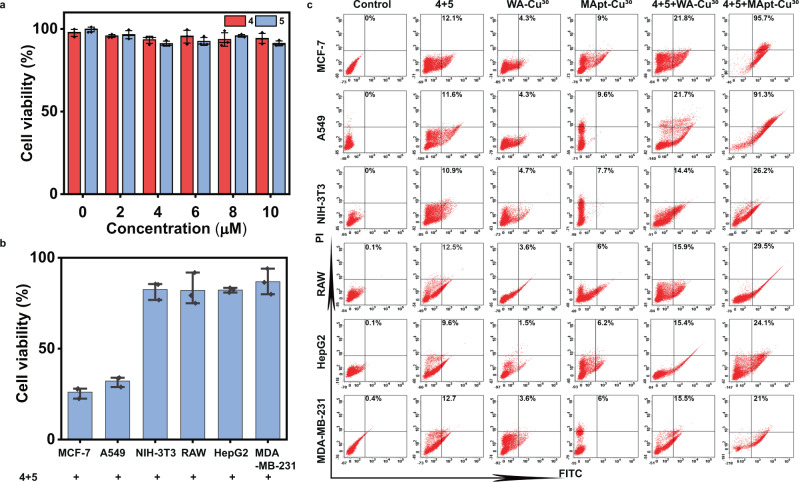
CuAAC reaction catalyzed by MApt-Cu^30^ in specific-type cells for prodrug activation. **a** Cytotoxicity of prodrug **4** and **5** on MCF-7 cells. Data were presented as mean ± SD (*n* = 3 independent experiments). **b** Cell viability of different cells treated with **4** + **5** + MApt-Cu^30^. The prodrug was activated and Rsv analogue was synthesized to assess the targeted therapeutic effect. Data were presented as mean ± SD (*n* = 3 independent experiments). **c** Flow cytometry analysis of the apoptotic proportion of different cells treated with **4** + **5**, WA**-**Cu^30^, MApt-Cu^30^, **4** + **5** + WA**-**Cu^30^ and **4** + **5** + MApt-Cu^30^, respectively. Source data are provided as a Source Data file.

As shown in Fig. 5a and Supplementary Figs. 35, 36, in the absence of MApt-Cu^30^, low cytotoxicity was found for the prodrugs **4** and **5** even at the concentration of 50 μM in various cells. Upon addition of MApt-Cu^30^, MUC1^+^ cells showed a notable decrease in cell viability while MUC1^-^ cells and normal cells showed a slight decrease (Fig. 5b, Supplementary Fig. 36). It implied that Rsv analogues **6** could be synthesized under the catalysis of CuNPs and resulted in a cytotoxicity to cells. The difference in cytotoxicity to MUC1^+^ cells and MUC1^-^/normal cells resulted from specific recognition of MUC1 aptamer. To further prove the effect of specific binding of MApt-Cu^30^ to MUC1 on prodrug activation, the control experiment was performed. First, a DNA strand complementary to MUC1 aptamer was mixed with MApt-Cu^30^ and denoted as MApt-Cu^30^-block (Supplementary Table 1). Then, CuNPs were synthesized using MUCI aptamer-T30 with two mutants and denoted as mutMApt-Cu^30^. MTT assay was carried out to investigate the prodrug activation by MApt-Cu^30^-block and mutMApt-Cu^30^. The results showed that the cell viabilities of MCF-7 treated with MApt-Cu^30^-block and mutMApt-Cu^30^ were higher than that treated with MApt-Cu^30^ (Supplementary Fig. 37). For MApt-Cu^30^-block, the complementary sequence could inhibit aptamer-protein binding. For mutMApt-Cu^30^, the mutant aptamer had much lower affinity to MUC1 compared to MUC1 aptamer. Therefore, the accumulation of the two CuNPs was much lower, resulting in lower catalytic efficiency. Flow cytometry analysis by double staining showed that the death of MUC1^+^ cell was primarily associated with apoptosis (Fig. 5c). As negative controls, the percentage of apoptosis was much lower in MUC1^−^ cells. Combined with HPLC (Supplementary Fig. 38) and LC-MS (Supplementary Fig. 39) analysis, it can be proved that the CuAAC reaction proceeded smoothly in the living cells and Rsv analogues **6** with the molecular weight of 270.0 was generated. These experiments demonstrated that MApt-Cu^30^ could significantly promote the CuAAC reaction for prodrug activation, thereby exhibiting stronger antitumor efficacy in specific type cancer cells and minimizing the side effects to normal cells.

### Targeted prodrug activation in vivo

With the outstanding targeted catalytic performance of MApt-Cu^30^ in living cells, we next investigated its behaviors in vivo. We initially evaluated the in vivo toxicology of MApt-Cu^30^ using *Caenorhabditis elegans* (*C. elegans*) model (Supplementary Fig. 40a). The N_2_ wild-type strain worms were chosen to monitor the toxicity and phenotype alteration. Similar to the control group, the worms incubated with MApt-Cu^30^ were completely paralyzed around 16 days (Supplementary Fig. 40b), suggesting the biocompatibility of MApt-Cu^30^ in vivo. Then the catalytic activity of MApt-Cu^30^ on precursors **1** and **2** in the *C. elegans* model was tested. A distinct fluorescence was observed in the experimental group which was fed with MApt-Cu^30^ and precursors (Supplementary Fig. 40c). The results showed that MApt-Cu^30^ could effectively achieve CuAAC reaction in vivo with biocompatibility.

Furthermore, we systematically studied the toxicology of MApt-Cu^30^ to further clarify their safety in vivo using athymic nude mouse model. Hemolysis test of MApt-Cu^30^ was first carried out. No or negligible hemolysis of red-blood cells occurred at concentrations ranging from 3.125 to 50 μM (Supplementary Fig. 41). After intravenous injection of the nanocatalyst into MCF-7 tumor bearing nude mice, the biodistribution of MApt-Cu^30^ was studied at 4 h, 12 h, and 24 h (Supplementary Fig. 42) via ICP-MS analysis. Owing to the high specificity and affinity of MUC1 aptamer to MUC1^+^ cells, the accumulation of MApt-Cu^30^ in MCF-7 tumor region was largely completed within 4 h. In addition, the accumulation of MApt-Cu^30^ was only decreased 16%, elucidating that MApt-Cu^30^ could be remained in tumors at a high concentration for at least 24 h. The results indicated MApt-Cu^30^ with favorable accumulation and retention could be used as effective modality for selective targeted cancer therapy. Hematology analysis and blood biochemical assay were performed to evaluate the long-term toxicity of MApt-Cu^30^. The parameters in the MApt-Cu^30^ group were compared with those in the control group (Supplementary Fig. 43). The injection of MApt-Cu^30^ had negligible impact on the mice growth (Supplementary Fig. 44). Hematoxylin and eosin (H&E) stained images showed that no organ damage or adverse effects occurred after administration of MApt-Cu^30^ (Supplementary Fig. 45). These biosafety results indicated MApt-Cu^30^ was nontoxic as well as favored their further bioapplications.

**Fig. 6:**
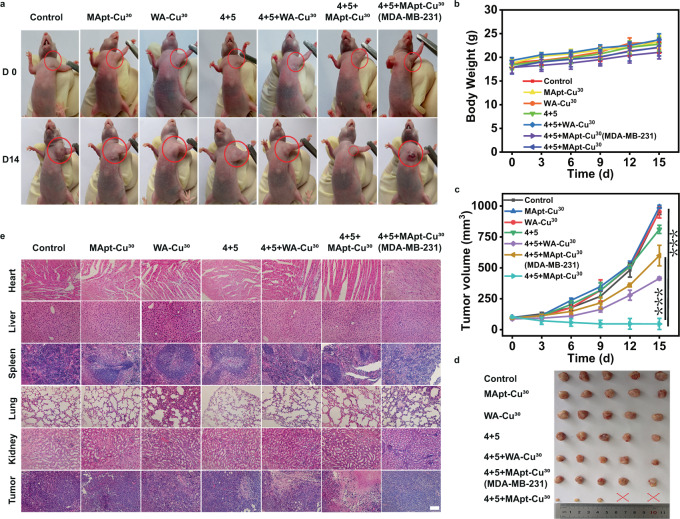
In vivo prodrug activation by MApt-Cu^30^-catalyzed CuAAC reaction for tumor therapy in tumor-bearing nude mice models. **a** Representative photographs of the nude mice bearing MCF-7 tumor before treatment (D0) and after 14 days of treatment (D14) in group of Control, MApt-Cu^30^, WA-Cu^30^, **4** + **5**, **4** + **5** + WA-Cu^30^, **4** + **5** + MApt-Cu^30^, and **4** + **5** + MApt-Cu^30^ (MDA-MB-231), respectively. **b** Changes of body weight of the mice in each group treated with different conditions. Data were presented as mean ± SD (*n* = 5). **c** Average tumor volume of the mice in each group treated with different conditions. Data were presented as mean ± SD (*n* = 5). Significance was assessed using Student’s t test (two-tailed). *P* values: 0.0000011 for MCF-7 tumor-bearing model treated with **4** + **5** + MApt-Cu^30^ vs MDA-MB-231 tumor-bearing model treated with **4** + **5** + MApt-Cu^30^; 0.000108 for MCF-7 tumor-bearing nude mice treated with **4** + **5** + MApt-Cu^30^ vs. Control. (***P < 0.001.) **d** Representative photographs of the excised tumors after different treatments on D14. **e** Representative images of H&E-stained slices after different treatment. Images are representative of three independent biological samples. Scale bars: 50 µm. Source data are provided as a Source Data file.

Encouraged by the satisfactory treatment result at the cellular level, we further explored the performance of MApt-Cu^30^ in CuAAC reaction in vivo for cancer therapy. MCF-7 tumor-bearing athymic nude mouse model, characterized by MUC1^+^ cell lines, was established (Fig. 6a). The mice were randomly divided into six groups (five mice per group): (1) control; (2) MApt-Cu^30^; (3) WA-Cu^30^; (4) prodrugs **4** + **5**; (5) **4** + **5** + WA-Cu^30^; (6) **4** + **5** + MApt-Cu^30^. Meanwhile, MDA-MB-231 tumor-bearing athymic nude mouse model, characterized by MUC1^-^ cell lines, was established for a comparison. The mice treated with **4** + **5** + MApt-Cu^30^ were named as group 7. We treated nude mice with MApt-Cu^30^ or WA-Cu^30^ nanocatalysts by intravenous injection (Supplementary Fig. 46). After 4 h, prodrugs **4** and **5** were administered intraperitoneally. PBS was used as the control. The administrations of nanocatalysts and prodrugs were repeated every 5 days. The body weights and tumor sizes of each group were monitored to conduct separate efficacy study during the course of treatments (Fig. 6b, c). By comparing the sizes of excised tumors mass from each group, it could be proved that **4** + **5** + MApt-Cu^30^ greatly reduced tumor growth rate and exhibited admirable antitumor efficacy in MCF-7 tumors model (Fig. 6d). As a result of significantly enhanced accumulation of MApt-Cu^30^ nanocatalyst in MCF-7 tumor, prodrugs could be effectively activated via CuAAC reaction. The staining assay showed apoptotic cells significantly increased, implying the tumors cells were badly damaged and adequately reduced (Fig. 6e). Above all, **4** + **5** + MApt-Cu^30^ possessed desirable therapeutic effect on in vivo tumor and negligible side effects to normal tissues, resulting from specific cancer cell-targeting ability of nanocatalyst for CuAAC reaction.

To further elucidate the behaviors in vivo, pharmacokinetic analysis of prodrug **4**, prodrug **5** and product **6** via HPLC was performed. As shown in Supplementary Table 5 and Supplementary Fig. 47, both prodrugs could reach maximum in the blood circulation system within 30 min, while the content of product **6** was relatively low. The result suggested very few **6** was synthesized through bioorthogonal reaction in the blood circulation system, demonstrating the specific targeting of the nanocatalyst.

In summary, a DNA-based nanocatalyst for bioorthogonal catalysis was successfully designed and constructed. The DNA strand not only served as the building block of nanocarrier and template of CuNPs, but also performed a specific recognition to target cells. The resulting nanocatalyst presented good biocompatibility, superior catalytic transformations, and type-specific cell recognition in vitro. The catalytic transformation efficiency was improved by an order of magnitude compared to the commonly used catalyst CuSO_4_/sodium ascorbate, and 40 fold compared to the nontargeting nanocatalyst. Molecular dynamics simulation was performed and showed that DNA structure and its interaction with substrates contributed to the powerful catalytic activity of the nanocatalyst. The system revealed a promising prospect in the practical use of prodrug activation in vivo. Animal studies using tumor-bearing mouse model validated our nanocatalyst and prodrugs with excellent tumor inhibition and negligible side effect to major organs. Furthermore, the programmability of nucleic acids enables the generality of aptamer-based nanocatalyst by simply using different aptamers. This proof-of-concept meant flexible targeting can be achieved, representing an easy and promising approach for the personalized therapy of cancers. It is expected this precision-targeted bioorthogonal nanocatalyst based on nucleic acid with superior performance to be a significant advance in the application of nanomaterials in biomedicine and the work will pave a new way for the treatment of important human diseases.

## Methods

### Preparation of DNA-based Cu nanoparticles

DNA strands (1 μM) containing thymine-rich regions were dissolved in 3-(N-Morpholino) propanesulfonic acid (MOPS) buffer (10 mM, pH 7.6) and sodium ascorbate solution (2 mM). After blending completely, CuSO_4_ solution (100 μM) was added and incubated for 15 min at room temperature. The mixtures were centrifuged via ultrafiltration centrifuge tube (retained molecular weight: 10 K) to remove unreacted substance. Fluorescent copper nanoparticles (CuNPs) were formed within 5 min (λex = 340 nm). The fluorescence spectra were generally obtained after five minutes reaction in our experiments. The optimized synthetic condition of Apt-Cu^30^ was obtained by testing different Cu (II) concentration and T30 concentration.

The oligonucleotide sequences used in the experiments are listed in a separate Supplementary Table.

### Catalytic performance in vial

Apt-Cu (with an equivalent amount of copper 5 µM), **1** (10 µM), and **2** (10 µM) were mixed in water at 25°C. The fluorescence intensities of the mixtures were measured by fluorescence spectrometer. The catalytic efficiency and kinetic process were measured by HPLC and fluorescence spectra.

### Molecular dynamics simulation of DNA structure

The timestep was set as 2 fs and the system was run for 30 ns. The last frame of simulation was used for docking. The RMSD values during the simulation indicated that the DNA sequence can reach a stable state in 20 ns. During the 30 ns simulation, the 15-base motif can quickly fold to a hairpin-like secondary structure. In contrast, the T30 motif did not form any stable secondary structures.

### Cellular internalization of MApt-Cu^30^ and WA-Cu^30^ quantified by ICP-MS

MCF-7 cells were preincubated with MApt-Cu^30^ (5 µM) or WA-Cu^30^ (5 μM) for different time, then washed with PBS for 3 times. After the cells were digested by trypsin, lysis buffer (300 µL) was added. The obtained cell lysate was treated with mixture of HNO_3_ and H_2_O_2_ (3:1 mL) overnight. Then aqua regia (3 mL) was added to the samples for another 2–3 h. The obtained samples were dried and re-dispersed into the water, followed by analysis using ICP-MS.

### MApt-Cu^30^-mediated click reactions in living cells

#### Confocal microscopy imaging

MCF-7, A549, NIH-3T3, RAW, MDA-MB-231 and HepG2 cells were seeded on sterilized cover slips for 6 h in 24-well plates. MApt-Cu^30^ or WA-Cu^30^ (5 µM) was added and incubated with the cells for 4 h. Then the cells were washed with PBS for 3 times. Next, azide coumarin **1** (10 µM) and alkyne **2** (10 µM) were added and incubated with the cells for 12 h, followed by washing with PBS for 2 times. After staining cytomembrane by DiOC18(3), the fluorescence images were taken by OLYMPUS-BX51 microscopes with U-25ND25 filter.

### Flow cytometric analysis

MCF-7, A549, NIH-3T3, RAW, MDA-MB-231, and HepG2 cells were plated in 6-wellplates. Apt-Cu^30^ (5 μM) was incubated with the cells for 4 h, and then washed with PBS for 3 times. Next, azide coumarin **1** (10 µM) and alkyne **2** (10 µM) were added and incubated with the cells for 12 h. After being washed with PBS for 2 times, the cells were harvested using trypsin and resuspended in PBS. The intracellular fluorescence of product **3** was analyzed using flow cytometry (450/50).

For MApt-Cu^30^-mediated synthesis of resveratrol analogue inside living cells, the experimental conditions were the same as above, except for replacing azide coumarin **1** and alkyne **2** with **3** and **4**. The apoptosis of these cells induced by resveratrol analogue was analyzed by double staining with Annexin VFITC and propidium iodide using commercial apoptosis detection kit. The flow cytometry data were obtained by BD LSRFortessa™ Cell Analyzer and analyzed using FlowJo_V10. software.

### LC-MS analysis

The MCF-7 cells were plated in 6-well plates for CuAAC reaction. Apt-Cu^30^ (5 μM) was incubated with MCF-7 cells for 4 h, then washed with PBS for 3 times. Then, azide coumarin **1** (10 µM) and alkyne **2** (10 µM) were added and incubated with the cells for 12 h. After the reaction was completed, the cells were treated with trypsin, collected in ultrapure water, and then lysed by sonication. The resulting lysate was centrifuged at 12,470 g for 5 min. The supernatant was extracted to mix with cold acetone and kept at −20 °C overnight. Then the mixture was centrifuged at 12,470 g for 15 min to collect the precipitate. The precipitate was re-dispersed in methanol and analyzed by LC-MS.

### Caenorhabditis elegans (C. elegans) experiments

Wild-type (N2) strain were cultured in nematode growth medium (NGM) containing *E. coli* (OP50) at 20°C. MApt-Cu^30^ (5 µM) was incubated with the N2 worms (about 50 worms per plate) for worm paralysis assay. After 4 h, worms were washed with PBS for 3 times to remove excess MApt-Cu^30^. Next, azide coumarin **1** (10 µM) and alkyne **2** (10 µM) were added, and incubated for 12 h. Worms were washed with PBS twice and fixed with 4% paraformaldehyde. The images of worms were taken using a fluorescence microscope after being washed by PBS.

### Animal experiments

Healthy six-week-old *BalB/c nude mice* (14~16 g) were purchased from the Laboratory Animal Center of Jilin University (Changchun, China). They were housed in a specific pathogen-free environment at 26 ± 1 °C and 50 ± 5% humidity, with a 12 h light-dark cycle. The handling procedures of animal were in accordance with the guidelines of the Animal Ethics Committee of Jilin University for Animal Experiments.

### The biocompatibility of MApt-Cu^30^ in athymic nude mouse

Healthy BalB/c-nu mice were randomly divided into two groups. The mice in control group and experimental group were intravenously injected with PBS and MApt-Cu^30^ (3.5 mg kg^−1^), respectively. Blood samples of the mice at different periods were collected for whole blood panel analysis and serum biochemical analysis. The blood biochemistry markers (WBC, RBC, MCV, HCT, MCH, MCHC, PLT and HGB), liver function markers (ALT, ALP, ALB, AST, and TP) and kidney function markers (BUN and CREA) were tests. Within 28 days after injection, the weight of mice was measured every 3 days. On the 28th day, the heart, liver, spleen, lung, and kidney of mice were collected, and fixed with 4% paraformaldehyde for subsequent H&E staining.

### Biodistribution and pharmacokinetics of the nanocatalyst in tumor-bearing mice

The mice were intravenously injected with MApt-Cu^30^ (3.5 mg kg^−1^). At 4, 12 and 24 h after injection, the mice were sacrificed. The organs and tumors were collected and digested in aqua regia at 80 °C. The accurate distribution was evaluated by ICP-MS.

### Targeted prodrug activation of MApt-Cu^30^ in CuAAC reaction for cancer therapy in vivo

MCF-7 tumor-bearing athymic nude mouse model, characterized by MUC1 + cell lines, was established. The mice were divided into six groups and treated under different conditions: (1) control; (2) MApt-Cu^30^; (3) WA-Cu^30^; (4) prodrugs **4** + **5**; (5) **4** + **5** + WA-Cu^30^; (6) **4** + **5** + MApt-Cu^30^. Meanwhile, MDA-MB-231 tumor-bearing athymic nude mouse model, characterized by MUC1- cell lines, was established for a comparison. The mice were intravenously injected with MApt-Cu^30^ or WA-Cu^30^ (3.5 mg kg^−1^), respectively. After 4 h, prodrugs **4** and **5** (20 mg kg^−1^) were administered intraperitoneally. PBS was used as the control.

### H&E staining

The major organs (heart, liver, spleen, lung, and kidney) of the mice in the above seven groups were collected, and fixed with neutral buffered formalin (10%). Then the organs were embedded into paraffin and sectioned into 4 μm thickness for H&E staining. Furthermore, to evaluate the pathological damages to tumors, the tumor tissues sections were collected for H&E staining. The images of these sections were taken using a microscope under bright field.

### Statistical analysis

All of the data were presented as the mean ± standard deviation (SD). All figures were obtained from a minimum of three independent experiments. Data analysis was performed using Origin 2020 and Excel 2018 software. Statistical evaluation was performed using two-tailed Student’s *t* test analysis. Asterisks indicated significant differences (*P  <  0.01, **P  <  0.005, ***P  <  0.001). Notably, no samples and animals were excluded from the analysis.

### Reporting summary

Further information on research design is available in the Nature Research Reporting Summary linked to this article.

## Supplementary information

Supplementary Information

Reporting Summary

## Data Availability

The source data underlying Figs. 1–6 and Supplementary Figures 1-2, 5-17, 22, 25, 32, 35-38, 40-44 are provided as a Source Data file. The data is available from the corresponding authors upon reasonable request. Source data are provided with this paper.

## Source data

Source Data

## Code availability

The original analysis code for molecular dynamics simulation is kept at CAS State Key Laboratory of Rare Earth Resources Utilization at Changchun Institute of Applied Chemistry and is available from the corresponding authors upon reasonable request.

## References

[1] Sancho-Albero M (2019). Cancer-derived exosomes loaded with ultrathin palladium nanosheets for targeted bioorthogonal catalysis. Nat. Catal..

[2] Ji X (2019). Click and release: bioorthogonal approaches to “on-demand” activation of prodrugs. Chem. Soc. Rev..

[3] Yang M, Li J, Chen PR (2014). Transition metal-mediated bioorthogonal protein chemistry in living cells. Chem. Soc. Rev..

[4] Yao Q (2018). Synergistic enzymatic and bioorthogonal reactions for selective prodrug activation in living systems. Nat. Commun..

[5] Tonga GY (2015). Supramolecular regulation of bioorthogonal catalysis in cells using nanoparticle-embedded transition metal catalysts. Nat. Chem..

[6] Chankeshwara SV, Indrigo E, Bradley M (2014). Palladium-mediated chemistry in living cells. Curr. Opin. Chem. Biol..

[7] Sasmal PK, Streu CN, Meggers E (2013). Metal complex catalysis in living biological systems. Chem. Commun..

[8] Volker T, Dempwolff F, Graumann PL, Meggers E (2014). Progress towards Bioorthogonal Catalysis with Organometallic Compounds. Angew. Chem. Int. Ed..

[9] Yusop RM, Unciti-Broceta A, Johansson EM, Sanchez-Martin RM, Bradley M (2011). Palladium-mediated intracellular chemistry. Nat. Chem..

[10] Streu C, Meggers E (2006). Ruthenium-Induced Allylcarbamate Cleavage in Living Cells. Angew. Chem. Int. Ed..

[11] Unciti-Broceta A, Johansson EM, Yusop RM, Sanchez-Martin RM, Bradley M (2012). Synthesis of polystyrene microspheres and functionalization with Pd(0) nanoparticles to perform bioorthogonal organometallic chemistry in living cells. Nat. Protoc..

[12] Rostovtsev VV, Green LG, Fokin VV, Sharpless KB (2002). A stepwise Huisgen cycloaddition process: Copper(I)-catalyzed regioselective “ligation” of azides and terminal alkynes. Angew. Chem. Int. Ed..

[13] Tornoe CW, Christensen C, Meldal M (2002). Peptidotriazoles on solid phase: [1,2,3]-triazoles by regiospecific copper(I)-catalyzed 1,3-dipolar cycloadditions of terminal alkynes to azides. J. Org. Chem..

[14] Meldal M, Tornøe CW (2008). Cu-Catalyzed Azide−Alkyne Cycloaddition. Chem. Rev..

[15] Porte K, Riomet M, Figliola C, Audisio D, Taran F (2021). Click and Bio-Orthogonal Reactions with Mesoionic Compounds. Chem. Rev..

[16] Chu CH, Liu RH (2011). Application of click chemistry on preparation of separation materials for liquid chromatography. Chem. Soc. Rev..

[17] Liang LY, Astruc D (2011). The copper(I)-catalyzed alkyne-azide cycloaddition (CuAAC) “click” reaction and its applications. An overview. Coord. Chem. Rev..

[18] Wang YQ, Weng JH, Lin JG, Ye DJ, Zhang Y (2020). NIR Scaffold Bearing Three Handles for Biocompatible Sequential Click Installation of Multiple Functional Arms. J. Am. Chem. Soc..

[19] Kennedy DC (2011). Cellular Consequences of Copper Complexes Used To Catalyze Bioorthogonal Click Reactions. J. Am. Chem. Soc..

[20] Hong V, Presolski SI, Ma C, Finn MG (2009). Analysis and Optimization of Copper-Catalyzed Azide-Alkyne Cycloaddition for Bioconjugation. Angew. Chem. Int. Ed..

[21] Clavadetscher J (2016). Copper Catalysis in Living Systems and In Situ Drug Synthesis. Angew. Chem. Int. Ed..

[22] Alonso F, Moglie Y, Radivoy G (2015). Copper Nanoparticles in Click Chemistry. Acc. Chem. Res..

[23] Uttamapinant C (2012). Fast, Cell-Compatible Click Chemistry with Copper-Chelating Azides for Biomolecular Labeling. Angew. Chem. Int. Ed..

[24] Liu K, Lat PK, Yu H-Z, Sen D (2020). CLICK-17, a DNA enzyme that harnesses ultra-low concentrations of either Cu+ or Cu2+ to catalyze the azide-alkyne ‘click’ reaction in water. Nucleic Acids Res..

[25] Chen JF, Li K, Bonson SE, Zimmerman SC (2020). A Bioorthogonal Small Molecule Selective Polymeric “Clickase”. J. Am. Chem. Soc..

[26] Jin TN, Yan M, Yamamoto Y (2012). Click Chemistry of Alkyne-Azide Cycloaddition using Nanostructured Copper Catalysts. ChemCatChem.

[27] You Y (2020). Near-Infrared Light Dual-Promoted Heterogeneous Copper Nanocatalyst for Highly Efficient Bioorthogonal Chemistry in Vivo. ACS Nano.

[28] Wang FM (2019). A Biocompatible Heterogeneous MOF-Cu Catalyst for In Vivo Drug Synthesis in Targeted Subcellular Organelles. Angew. Chem. Int. Ed..

[29] Mo R (2012). Multistage pH-responsive liposomes for mitochondrial-targeted anticancer drug delivery. Adv. Mater..

[30] Pinheiro AV, Han DR, Shih WM, Yan H (2011). Challenges and opportunities for structural DNA nanotechnology. Nat. Nanotechnol..

[31] Seeman NC (2010). Nanomaterials Based on DNA. Annu. Rev. Biochem..

[32] Liu X, Lu C-H, Willner I (2014). Switchable Reconfiguration of Nucleic Acid Nanostructures by Stimuli-Responsive DNA Machines. Acc. Chem. Res..

[33] Qing Z (2013). Poly(thymine)-templated selective formation of fluorescent copper nanoparticles. Angew. Chem. Int. Ed..

[34] Chen Y, Phipps ML, Werner JH, Chakraborty S, Martinez JS (2018). DNA Templated Metal Nanoclusters: From Emergent Properties to Unique Applications. Acc. Chem. Res..

[35] Chen Z, Liu C, Cao F, Ren J, Qu X (2018). DNA metallization: principles, methods, structures, and applications. Chem. Soc. Rev..

[36] Zhou L, Ren J, Qu X (2017). Nucleic acid-templated functional nanocomposites for biomedical applications. Mater. Today.

[37] Fang X, Tan W (2010). Aptamers Generated from Cell-SELEX for Molecular Medicine: A Chemical Biology Approach. Acc. Chem. Res..

[38] Tan W, Donovan MJ, Jiang J (2013). Aptamers from Cell-Based Selection for Bioanalytical Applications. Chem. Rev..

[39] Ouyang C (2020). Precision-Guided Missile-Like DNA Nanostructure Containing Warhead and Guidance Control for Aptamer-Based Targeted Drug Delivery into Cancer Cells in Vitro and in Vivo. J. Am. Chem. Soc..

[40] Zhou J, Rossi J (2017). Aptamers as targeted therapeutics: current potential and challenges. Nat. Rev. Drug. Discov..

[41] Li Y, Liu J (2021). Nanozyme’s catching up: activity, specificity, reaction conditions and reaction types. Mater. Horiz..

[42] Zhao L (2020). The DNA controllable peroxidase mimetic activity of MoS2 nanosheets for constructing a robust colorimetric biosensor. Nanoscale.

[43] Liu B, Liu J (2015). Accelerating peroxidase mimicking nanozymes using DNA. Nanoscale.

[44] Yang B, Chen Y, Shi J (2019). Nanocatalytic Medicine. Adv. Mater..

[45] Huo M, Wang L, Chen Y, Shi J (2017). Tumor-selective catalytic nanomedicine by nanocatalyst delivery. Nat. Commun..

[46] Brayman M, Thathiah A, Carson DD (2004). MUC1: a multifunctional cell surface component of reproductive tissue epithelia. Reprod. Biol. Endocrinol..

[47] Xuan W (2019). Molecular Self-Assembly of Bioorthogonal Aptamer-Prodrug Conjugate Micelles for Hydrogen Peroxide and pH-Independent Cancer Chemodynamic Therapy. J. Am. Chem. Soc..

[48] Xu FZ (2018). Label-free and sensitive microRNA detection based on a target recycling amplification-integrated superlong poly(thymine)-hosted copper nanoparticle strategy. Anal. Chim. Acta.

[49] Laskowski RA, Swindells MB (2011). LigPlot+: multiple ligand-protein interaction diagrams for drug discovery. J. Chem. Inf. Model..

[50] Reuter JS, Mathews DH (2010). RNAstructure: software for RNA secondary structure prediction and analysis. BMC Bioinforma..

[51] Clavadetscher J, Indrigo E, Chankeshwara SV, Lilienkampf A, Bradley M (2017). In-Cell Dual Drug Synthesis by Cancer-Targeting Palladium Catalysts. Angew. Chem. Int Ed..

[52] Indrigo E, Clavadetscher J, Chankeshwara SV, Lilienkampf A, Bradley M (2016). Palladium-mediated in situ synthesis of an anticancer agent. Chem. Commun..

[53] Neumann K, Gambardella A, Lilienkampf A, Bradley M (2018). Tetrazine-mediated bioorthogonal prodrug-prodrug activation. Chem. Sci..

[54] Geng J (2021). Switching on prodrugs using radiotherapy. Nat. Chem..

[55] Xianfeng-Huang, Zhu HL (2011). Resveratrol and Its Analogues: Promising Antitumor Agents. Anti-Cancer Agents Med. Chem..

[56] Fulda S (2010). Resveratrol and derivatives for the prevention and treatment of cancer. Drug Discov. Today.

