# Author Correction: DNA-based platform for efficient and precisely targeted bioorthogonal catalysis in living systems

**DOI:** 10.1038/s41467-022-31528-5

**Published:** 2022-06-28

**Authors:** Yawen You, Qingqing Deng, Yibo Wang, Yanjuan Sang, Guangming Li, Fang Pu, Jinsong Ren, Xiaogang Qu

**Affiliations:** 1 State Key Laboratory of Rare Earth Resources Utilization and Laboratory of Chemical Biology, Changchun Institute of Applied Chemistry, Chinese Academy of Sciences, Changchun, 130022 P. R. China; 2 University of Science and Technology of China, Hefei, Anhui 230029 P. R. China; 3 Laboratory of Chemical Biology, Changchun Institute of Applied Chemistry, Chinese Academy of Sciences, Changchun, 130022 P. R. China

**Keywords:** Biotechnology, Biomaterials, Catalysis

Correction to: *Nature Communications* 10.1038/s41467-022-29167-x, published online 18 March 2022.

In the version of this article initially published, affiliation 1 and 2 were listed in the wrong order. The order has been corrected to read: 1, State Key Laboratory of Rare Earth Resources Utilization and Laboratory of Chemical Biology, Changchun Institute of Applied Chemistry, Chinese Academy of Sciences, Changchun 130022, P. R. China and 2, University of Science and Technology of China, Hefei, Anhui 230029, P. R. China. This has been corrected in both the PDF and HTML versions of the Article.

