# Supplementary material for: DNA-based platform for efficient and precisely targeted bioorthogonal catalysis in living systems
Source: Nat Commun. 2022 Mar 18;13:1459. doi: 10.1038/s41467-022-29167-x (PMC8933418; doi:10.1038/s41467-022-29167-x)
Supplement: Supplementary file 1 — Supplementary Information [file 41467_2022_29167_MOESM1_ESM.pdf]

Supplementary Information

DNA-Based Platform for Efficient and Precisely Targeted Bioorthogonal Catalysis in Living Systems

Yawen You,[1,2] *Qingqing Deng,* [1,2] *Yibo Wang,* [3] *Yanjuan Sang,*[1,2] *Guangming Li,* [*,2] *Fang Pu,* [*, 1,2] *Jinsong Ren,* [*, 1,2] *and Xiaogang Qu*[*,1,2]

AUTHOR ADDRESS

[1] University of Science and Technology of China, Hefei, Anhui 230029, P. R. China.

[2] State Key Laboratory of Rare Earth Resources Utilization and Laboratory of Chemical Biology, Changchun Institute of Applied Chemistry, Chinese Academy of Sciences, Changchun 130022, P. R. China.

[3]Laboratory of Chemical Biology, Changchun Institute of Applied Chemistry, Chinese Academy of Sciences, Changchun 130022, P. R. China.

[*]email: lgm@ciac.ac.cn, pufang@ciac.ac.cn, jren@ciac.ac.cn, xqu@ciac.ac.cn

Contents

Supplementary 1. General Information 3
Reagents and materials 3
Measurements and characterizations 5
Supplementary 2. Preparation and Characterization of DNA-based CuNPs 6
Supplementary 3. Apt-Cu Promoted Click Reaction in vial 11
In silico simulations 17
Supplementary 4. Activating the Fluorescence Probe by MApt-Cu^{30} inside Cell 19
Cellular culture 19
Cell viability assay 19
Cellular internalization of MApt-Cu^{30} and WA-Cu^{30} 21
MApt-Cu^{30} mediated click reactions in living cells 25
Fluorescence imaging for studying the catalytic activity of AApt-Cu^{30} in living cells 31
Supplementary 5. Synthesis of Resveratrol Analogue Catalyzed by MApt-Cu^{30} in Living Cells. . 33
Supplementary 6. Targeted prodrug activation in vivo for Tumor Therapy 37
CuAAC reaction catalyzed by MApt-Cu^{30} in *C. elegans* model. 37
Animal Experiments. 37
The biocompatibility of the MApt-Cu^{30} in mice. 38
Pharmacokinetic analysis of prodrug 4, prodrug 5 and product 6 42
Supplementary 7. Synthesis of Chemical Substrate Molecules 45
3-Azido-7-hydroxy-chromen-2-one (3-Azido-7-hydroxycoumarin) (1) 45
7-hydroxy-3-(4-phenyl-1H- [1,2,3] triazole-1-yl)-coumarin (3) 47
5-azidobenzene-1,3-diol (4) 48
The precursor of 4-ethynylphenol (5) 4-((trimethylsilyl)ethynyl) phenol 50
4-ethynylphenol (5) 51
5-(4-(4-hydroxyphenyl)-1H-1,2,3-triazol-1-yl) benzene-1,3-diol (6) 52
References 53

Supplementary 1. General Information

Reagents and materials

Nanopure water (Millpore Co., 18.2 MΩ; USA) was used in all experiments. All oligonucleotides were purchased from Sangon Biotechnology Inc. (Shanghai, China), and used without further purification. The oligonucleotide sequences used in this work were listed in Supplementary Table 1. The stock solutions of DNA were obtained via dissolving DNA in nanopure water. The reaction buffer contained 10 mM MOPS (150 mM NaCl, pH 7.6). Palladium (II) (Pd $(PPh_3)_2Cl_2$), 3-(N-morpholino) propanesulfonic acid (MOPS), sodium ascorbate, Dichlorobis (triphenyl-phosphine) ethynyltrimethylsilane (TMS), sodium azide (NaN_3), phloroglucinol, iodocopper (CuI), 4-iodophenol and 4-dimethylaminopyridine (DMAP) were obtained from Alfa Aesar. Copper sulfate anhydrous ($CuSO_4$), phloroglucinol, dimethyl sulfoxide-D6, acetone-d6, Chloroform-D and dimethyl formamide (DMF) were obtained from Aladdin Reagent (Shanghai, China). Sodium chloride, sodium hydroxide (NaOH), dichloromethane (CH_2Cl_2), methyl alcohol (MeOH), $NaNO_2$, NaH_2PO_4, Na_2HP_4, anhydrous sodium acetate, acetic anhydride, ammonia water ($NH_3 \cdot H_2O$), conc. hydrochloric acid (conc. HCl) and conc. nitric acid (conc. HNO_3) were purchased from Beijing Chemicals (Beijing, China). 3-(4, 5-dimethyl-2-yl)-2, 5-diphenyltetrazolium bromide (MTT) was purchased from Sangon Biotechnology Inc. (Shanghai, China). 3,3′-dioctadecyloxacarbocyanine perchlorate (DiO-C1038) was purchased from Beyotime Biotechnology. (Shanghai, China).

All other reagents were of analytical reagent grade and used as received.

Supplementary Table 1. Oligonucleotides used in the study.

Name	Sequence (from 5' to 3')
MUC1-T20	TTTTTTTTTTTTTTTTTTTTCAACATCATCAACACGGC TATAGCACATGGGTAAAACGAC
MUC1-T30	TTTTTTTTTTTTTTTTTTTTTTTTTTTTTTCAACATCAT CAACACGGCTATAGCACATGGGTAAAACGAC
MUC1-T40	TTTTTTTTTTTTTTTTTTTTTTTTTTTTTTTTTTTTTTTT CAACATCATCAACACGGCTATAGCACATGGGTAAAAC GAC
WA-T30	TTTTTTTTTTTTTTTTTTTTTTTTTTTTTTCAACATCAT CAACAC
AS1411-T30	GGTGGTGGTGGTTGTGGTGGTGGTGGCAACATCATC AACACTTTTTTTTTTTTTTTTTTTTTTTTTTTTTT
AS1411	GGTGGTGGTGGTTGTGGTGGTGGTGG
MUC1-T30-FAM	TTTTTTTTTTTTTTTTTTTTTTTTTTTTTTCAACATCAT CAACACGGCTATAGCACATGGGTAAAACGAC-FAM
AS1411-T30-FAM	GGTGGTGGTGGTTGTGGTGGTGGTGGCAACATCATC AACACTTTTTTTTTTTTTTTTTTTTTTTTTTTTTT-FAM
MUC1(block)	GTCGTTTTACCCATGTGCTATAGCC
MUC1(mutational) -T30	TTTTTTTTTTTTTTTTTTTTTTTTTTTTTTCAACATCAT CAACACGGC**AT**TAGCACATGGGTAAAACGAC
T30-linker-random	TTTTTTTTTTTTTTTTTTTTTTTTTTTTTTCAACATCAT CAACACATCGATCGATCGATCGATCGATCGA

Note: The underlined parts are the sequences of two aptamers: MUC1 and AS1411.

Measurements and characterizations

Transmission electron microscopic (TEM) images were captured by a FEI TECNAI G2 20 high-resolution transmission electron microscope (200 kV). The zeta-potential was measured in a Zetasizer 3000HS analyzer. The X-ray photoelectron Spectroscopy (XPS) spectra were analyzed by Thermo Fisher Scientific ESCALAB 250Xi Spectrometer Electron Spectroscopy (USA). ^{1}H NMR spectrum was recorded via a Bruker-600 MHz NMR instrument. The DNA concentration was measured with a JASCOV-550 spectrophotometer (JASCO International Co., LTD., Japan), equipped with a temperature-controlled cuvette holder controlled via using a circulating bath. ICP-MS measurements were performed on a TheroScientific Xseries II inductively coupled plasma mass spectrometer. The Liquid Chromatography Mass Spectra (LCMS) was obtained using Quattro Premier XE (USA). Fluorescence spectra were detected by JASCO F-6000 fluorescence spectrometer with a Peltier temperature control accessory. The high-performance liquid chromatography (HPLC) was measured by Ultimate 3000. The confocal laser scanning microscopy (CLSM) characterization was acquired by a top-of-the-line motorized upright (Nikon Eclipse Ni-E, Japan). The flow cytometry data were detected by BD LSRFortessa™ Cell Analyzer.

Supplementary 2. Preparation and Characterization of DNA-based CuNPs

The excitation spectrum of CuNPs in H_2O was measured via fluorescence spectrometer. The peak of excitation wavelength is 340 nm (Figure a). Fluorescent CuNPs were formed within 5 minutes, judging by fluorescence spectra in Figure b (λex= 340 nm). So we generally measured fluorescence spectra after five minutes of reaction.

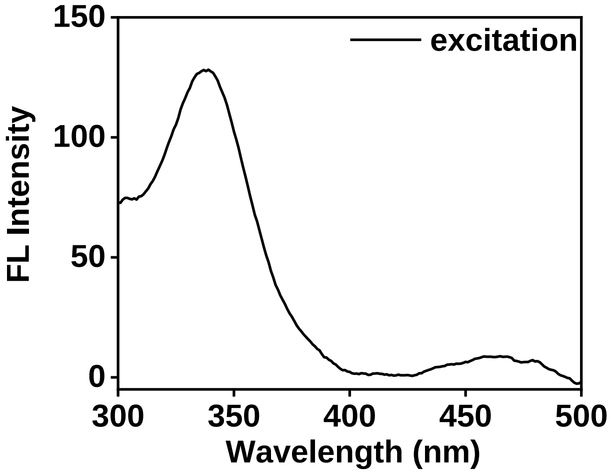

Figure a. Fluorescent excitation spectrum of Apt-Cu[30]. (λex=340 nm)

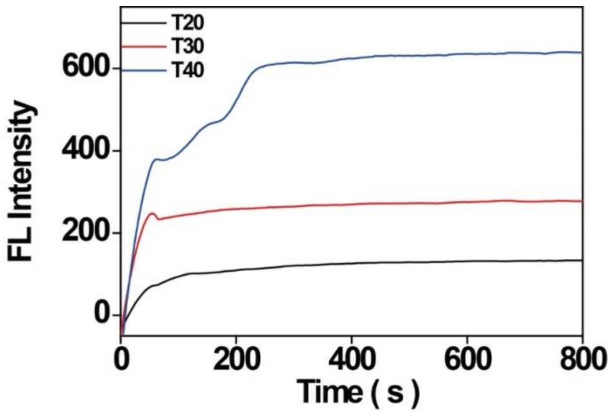

Figure b. Real-time fluorescence intensity of CuNPs recorded at 600 nm using different length of poly T as template.

For achieving the balance of high reaction rates and stability with low catalyst loadings, we further obtained the optimized synthetic condition of Apt-Cu[30] by testing different Cu^{2+} concentration (Figure c) and T30 concentration (Figure d).

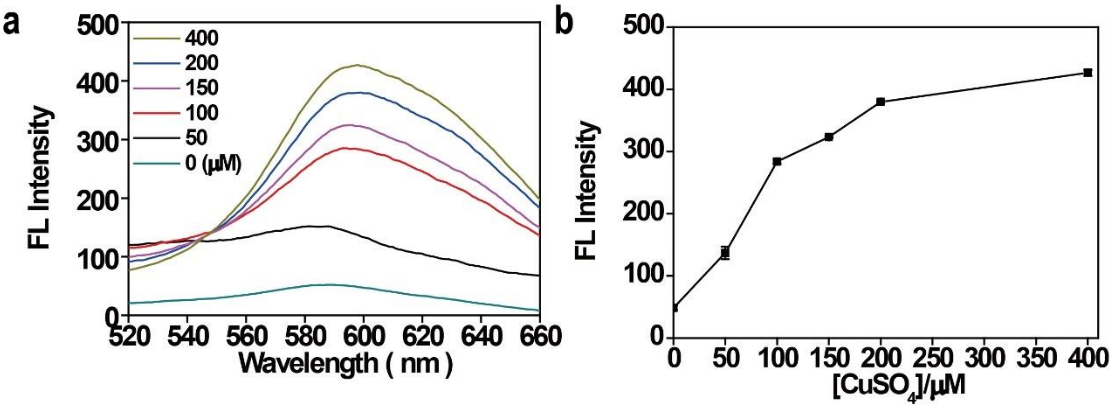

Figure c. Influence of Cu^{2+} concentration on the formation of fluorescent Apt-Cu[30] in the presence of 500 nM T30. **a**) Fluorescence spectra of Apt-Cu[30] under different concentrations of Cu^{2+}. **b**) Increase of fluorescence intensity at λem=600 nm (λex=340 nm) under different concentrations of the Cu^{2+}.

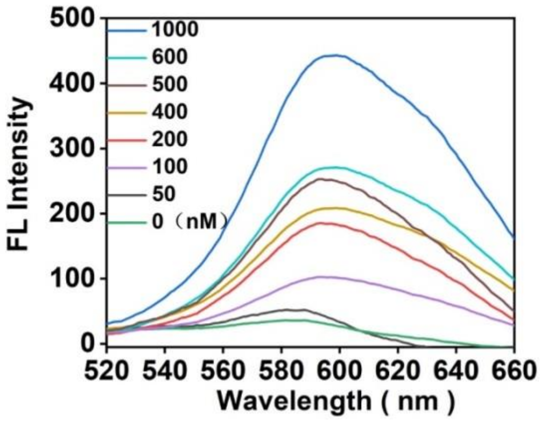

Figure d. Influence of T30 concentration in the presence of 100 μM Cu^{2+} during the formation of Apt-Cu[30]. Fluorescence spectra of Apt-Cu[30] using different concentrations of T30.

Supplementary Table 2. The content of DNA-templated copper nanocatalysts after synthesis.

	Catalyst	Cu (ppb)
1	MApt-Cu^{20}	782.5
		792.4
		772.6
2	MApt-Cu^{30}	2605
		2553
		2579
3	MApt-Cu^{40}	1766
		1774
		1758
4	AApt-Cu^{20}	701
		722
		718
5	AApt-Cu^{30}	2598
		2557
		2557
6	AApt-Cu^{40}	1807
		1793
		1778

Note: In the catalytic study, the nanocatalysts with an equivalent amount of copper were tested.

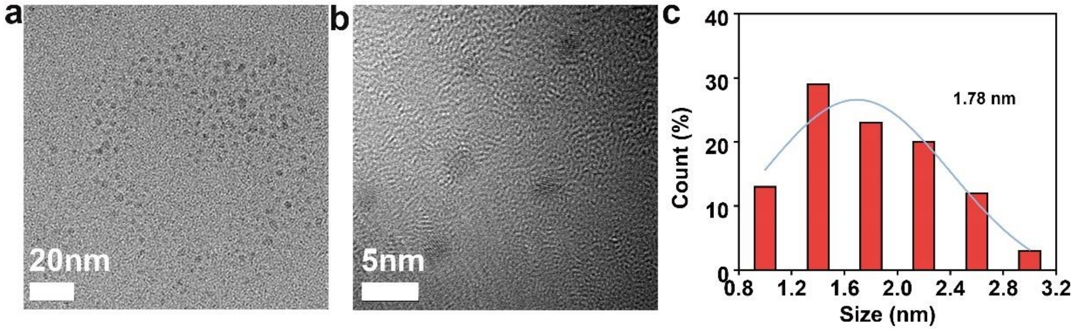

Supplementary Figure 1. a, **b**) TEM images of Apt-Cu^{20} under different magnifications. **c**) Histogram of size distribution of Apt-Cu^{20}. The average diameter of Apt-Cu^{20} is 1.78 nm.

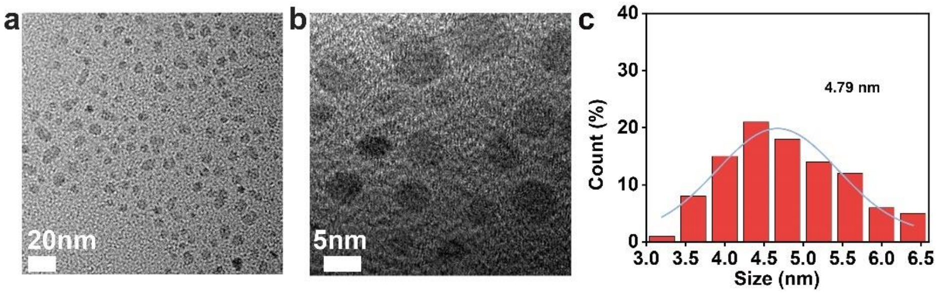

Supplementary Figure 2. a, b) TEM images of Apt-Cu[40] under different magnifications. **c**) Histogram of size distribution of Apt-Cu[40]. The average diameter of Apt-Cu[40] is 4.79 nm.

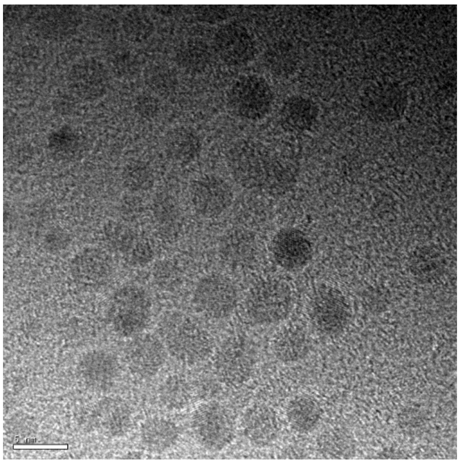

Supplementary Figure 3. TEM image of Apt-Cu[30] in DMEM with 10% FBS.

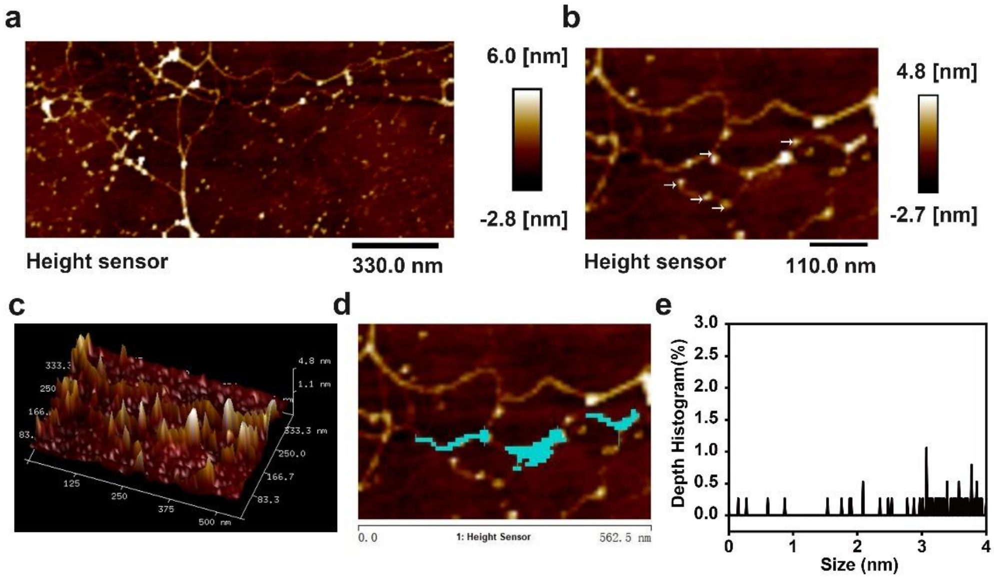

Supplementary Figure 4. Atomic force microscope (AFM) images of T30-templated CuNPs deposited on silicon. **a**) Image of a large area of T30-templated CuNPs. Scale bar is 330 nm. **b**) Enlarged image. The arrows indicate CuNPs on DNA template. Scale bar is 110 nm. Colour scales are presented next to AFM height image. **c**) 3D-view of AFM image. **d**) Particle analysis of enlarged image. **e**) Depth histogram analysis of T30-templated CuNPs. Images are representative of three independent biological samples.

Supplementary 3. Apt-Cu Promoted Click Reaction in vial

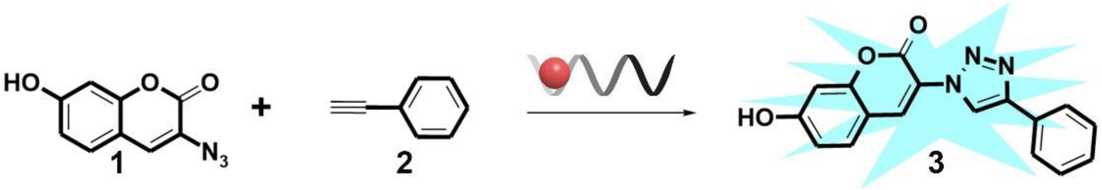

The click reaction between 3-azido-7-hydroxycoumarin, **1**, and phenylacetylene, **2** to form triazole **3** in vial, was carried out to assess the catalytic efficiency of Apt-Cu. Briefly, Apt-Cu, 10 µM **1** (4 mM in DMSO) and **2** were mixed at 25°C in water. We synthesized Apt-Cu with different length of poly T segment in the ssDNA. Apt-Cu^{20}, Apt-Cu^{30} and Apt-Cu^{40} were incubated with **1** and **2** in vial, respectively. The mixture was instantaneously showed cyan-blue fluorescence, demonstrating the superior catalytic activity of the Apt-Cu. The fluorescence of the mixture was detected via fluorescence spectrometer.

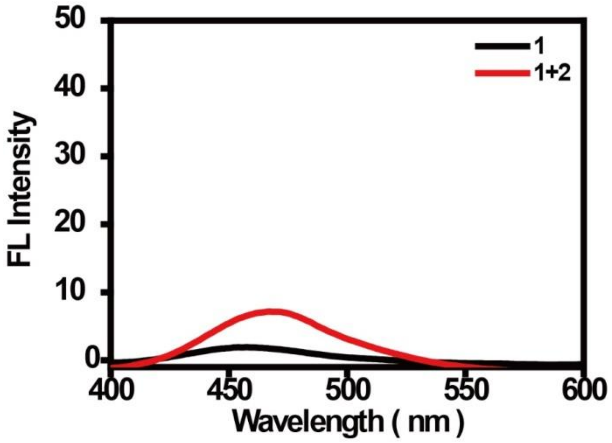

Supplementary Figure 5. The fluorescence spectra of the precursors **1** and **2**.

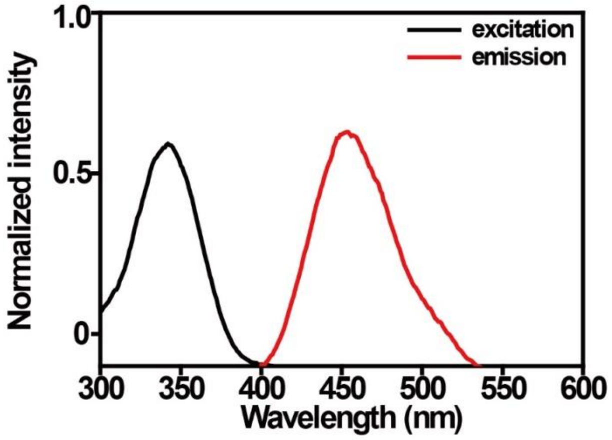

Supplementary Figure 6. Normalized excitation and emission of **3** in H_2O. Cycloaddition of **1** and **2** afforded **3** (λ_{ex} =340 nm and λ_{em} =460 nm).

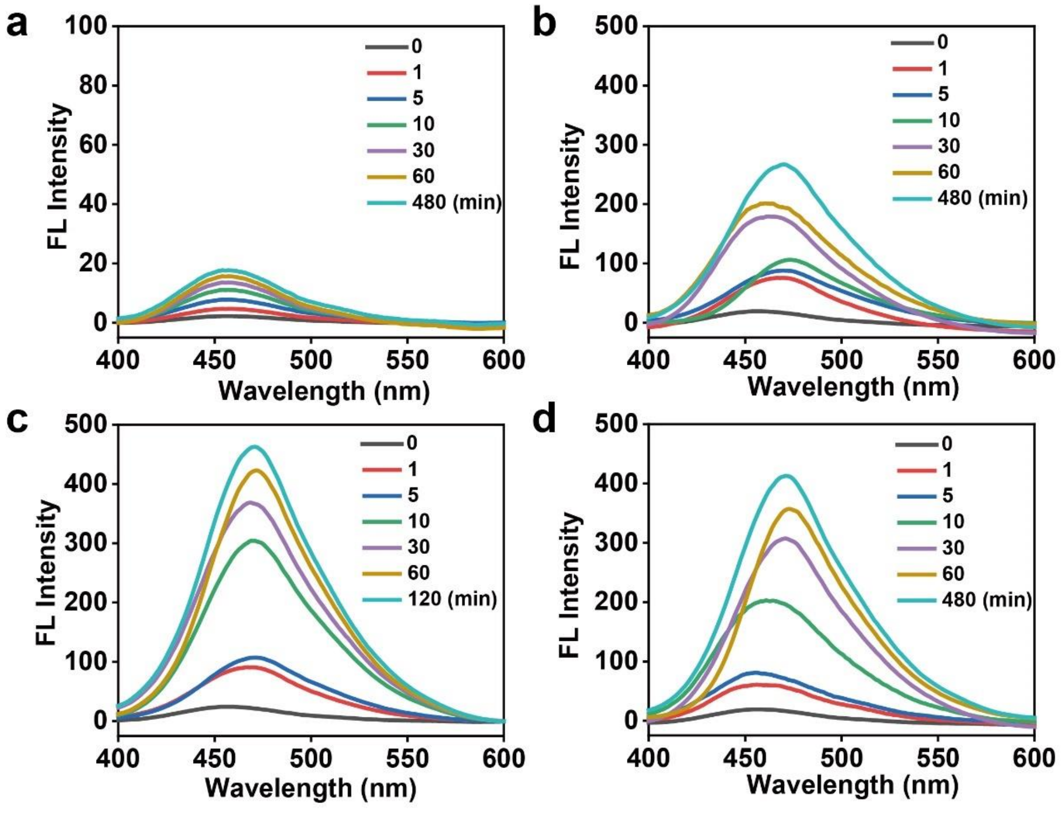

Supplementary Figure 7. Fluorescent spectra of Apt-Cu catalyzed CuAAC reaction with different reaction time in vial. **a**) **1** and **2**; **b, c, d**) Apt-Cu^{20}, Apt-Cu^{30} and Apt-Cu^{40} were catalyzed with **1** and **2** in vial, respectively.

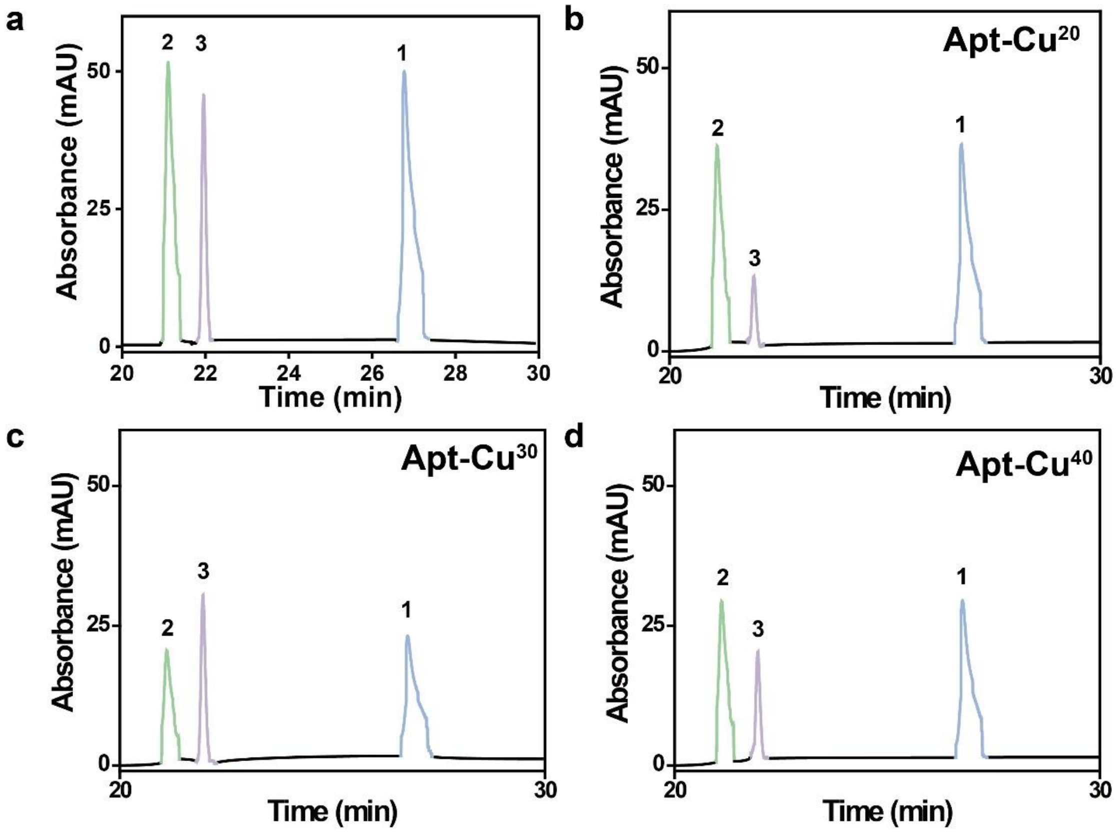

Supplementary Figure 8. HPLC analysis of CuAAC reaction catalyzed by Apt-Cu at 10 min. **a**) HPLC chromatogram of the catalytic reaction showed compound **1** at retention time 26.7 min, compound **2** at 21.4 min and product **3** at 22.5 min. **b-d**) HPLC chromatogram of catalytic reaction of **1** and **2** at 10 min by Apt-Cu^{20}, Apt-Cu^{30} and Apt-Cu^{40}, respectively.

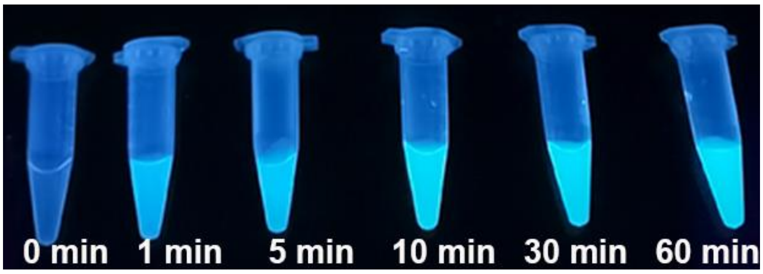

Supplementary Figure 9. Fluorescence image of the CuAAC reaction in H_2O catalyzed by Apt-Cu^{30} at different time (0, 1, 5, 10, 30, 60 min).

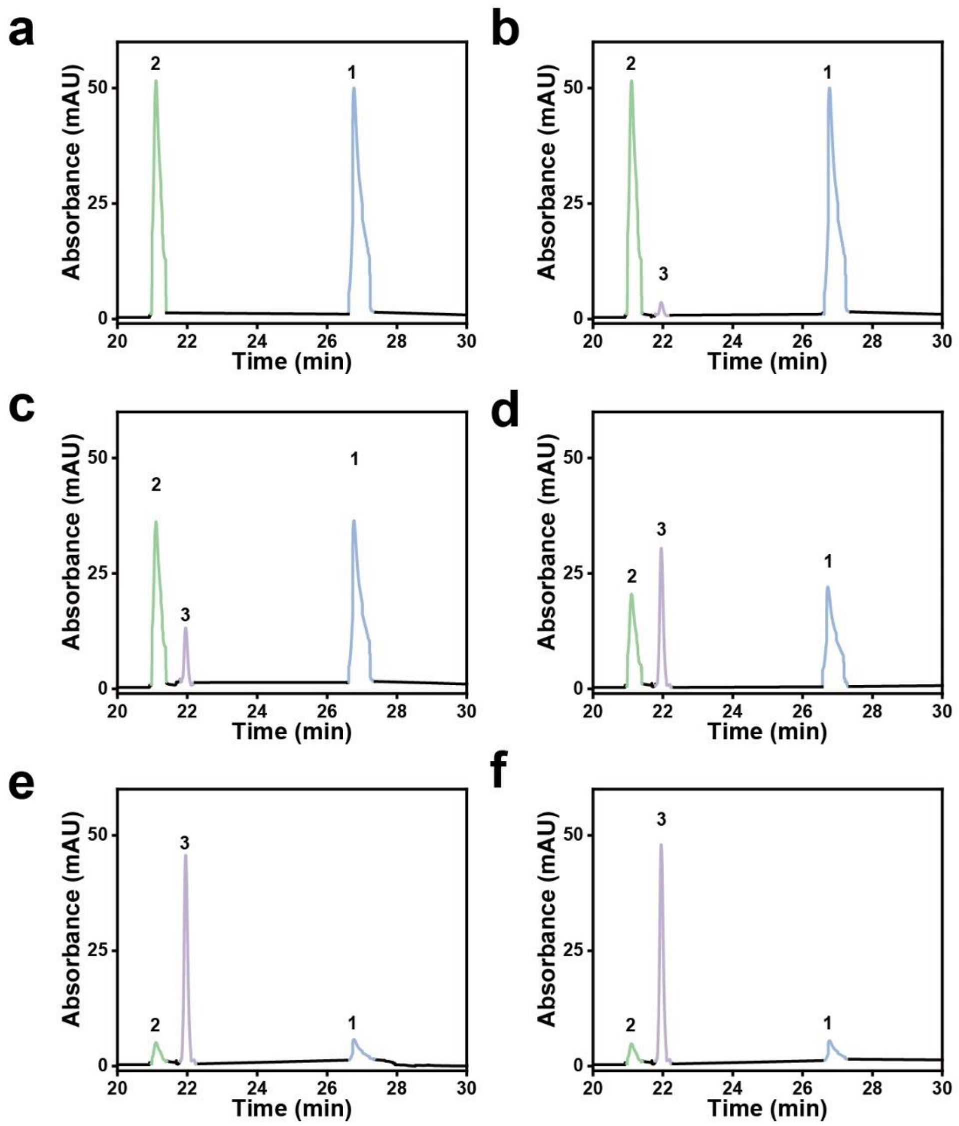

Supplementary Figure 10. HPLC analysis of CuAAC reaction catalyzed by Apt-Cu^{30} at different reaction time (0-60 min). **a-f**) The HPLC chromatogram of catalytic reaction with **1** and **2** co-incubation at 0, 1, 5, 10, 30, 60 min with Apt-Cu^{30}, respectively. HPLC chromatogram showing compound 2 at retention time 21.4 min, compound 1 at 26.7 min and product 3 at 22.5 min.

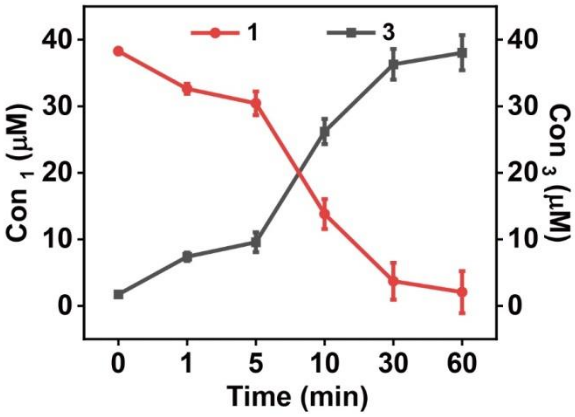

Supplementary Figure 11. The concentration changes of reactant and product in the CuAAC reaction catalyzed by Apt-Cu^{30} along with time (0-60 min). Error bars represented the standard deviation (n = 3 independent experiments). Data were presented as mean ± SD. Source data are provided as a Source Data file.

Supplementary Table 3. The catalytic efficiency of CuNPs for CuAAC in water.

	Catalyst	NaAsc	t (h)	Yield (%)
1	0	-	0.5	2.3
2	0	-	1	3.5
3	0	-	8	3.8
4	$CuSO_4$	-	0.5	3.2
5	$CuSO_4$	+	0.5	3.4
6	WA	-	0.5	5.1
7	Apt-Cu^{30}	-	0.5	90
8	$CuSO_4$	-	1	3.5
9	$CuSO_4$	+	1	7.8
10	WA	-	1	17.6
11	Apt-Cu^{30}	-	1	95
12	$CuSO_4$	-	8	4.6
13	$CuSO_4$	+	8	21.7
14	WA	-	8	21.2
15	Apt-Cu^{20}	-	8	62.1
16	Apt-Cu^{30}	-	8	>99
17	Apt-Cu^{40}	-	8	95.8

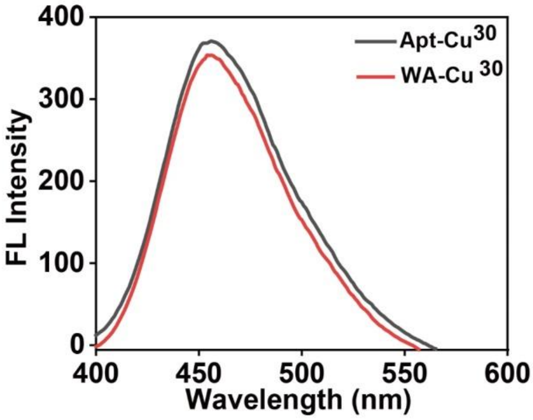

Supplementary Figure 12. Fluorescence spectra of Apt-Cu^{30}- and WA-Cu^{30}- catalyzed CuAAC reaction in H_2O. The concentration of nanocatalyst was 5 μM.

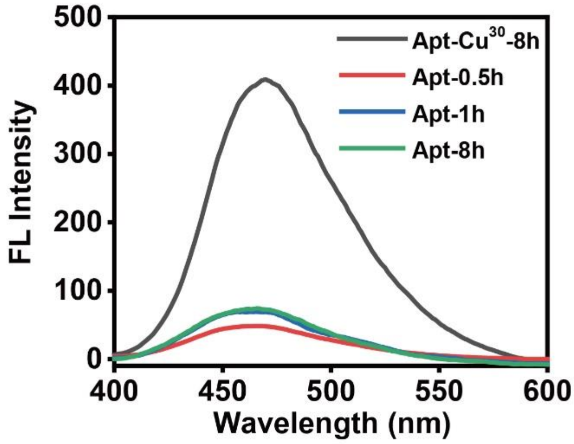

Supplementary Figure 13. Fluorescent spectra of click reaction using DNA strand without thymine-rich region at different time points in vial.

Supplementary Table 4. The catalytic efficiency of DNA sequences for click reaction in water.

	Catalyst	t (h)	Yield (%)
**1**	aptamer-spacer	**0.5**	**6.5**
**2**	aptamer-spacer	**1**	**11.4**
**3**	aptamer-spacer	**8**	**12.6**

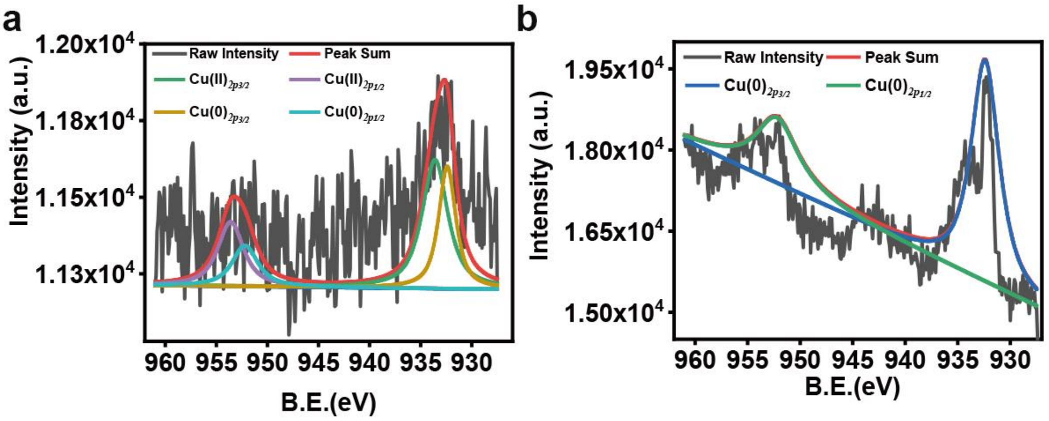

Supplementary Figure 14. XPS spectra of Apt-Cu[20] and Apt-Cu[40]. (arbitrary units, a. u.)

***In silico* simulations**

The single-stranded DNA model was built manually based on the nucleic acid secondary structure prediction web server, RNA structure 6.0.1.[1] The model was solvated in TIP3P water molecules and KCl (150 mM) in a 98×98×98 $Å^3$ box. The system simulated by the NAMD2.12 package[2] with CHARMM C36 force field[3-4] to pursue a stable conformation. It was run at the temperature of 310.15 K and the pressure of 1 atm. The Nosé-Hoover Langevin piston method[5-6] was applied for the pressure control and the Langevin thermostat for the temperature coupling. The Particle Mesh Ewald algorithm was used to treat Long-range electrostatic interactions.[7] 10-12 Å was employed to switch off the non-bonded interactions. The timestep was set as 2 fs and the system was run for 30 ns. The last frame of simulation was used for docking.

The docking poses of ligands were determined by AutoDock Vina 1.1.2.[8] Optimal binding sites were searched in a box of 30 Å × 30 Å × 30 Å, in which the best poses were picked for further analysis. The other options of AutoDock Vina were set as default. The binding interactions between DNA and ligand was analyzed by LigPlot+.[9]

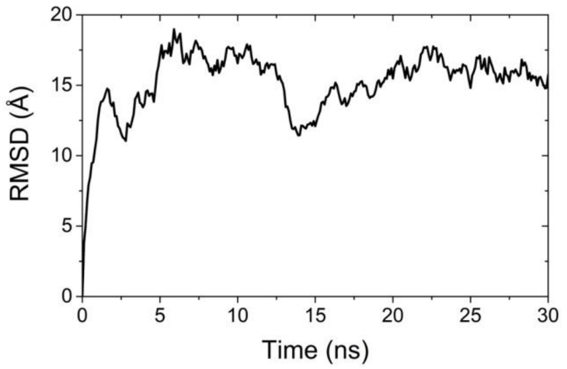

Supplementary Figure 15. The molecular dynamics simulation of DNA structure. The timestep was set as 2 fs and the system was run for 30 ns. The last frame of simulation was used for docking. The root-mean-square deviation (RMSD) values during the simulation indicated that the DNA sequence can reach a stable state in 20 ns. During the 30 ns simulation, the 15-base motif can quickly fold to a hairpin-like secondary structure. In contrast, the T30 motif did not form any stable secondary structures.

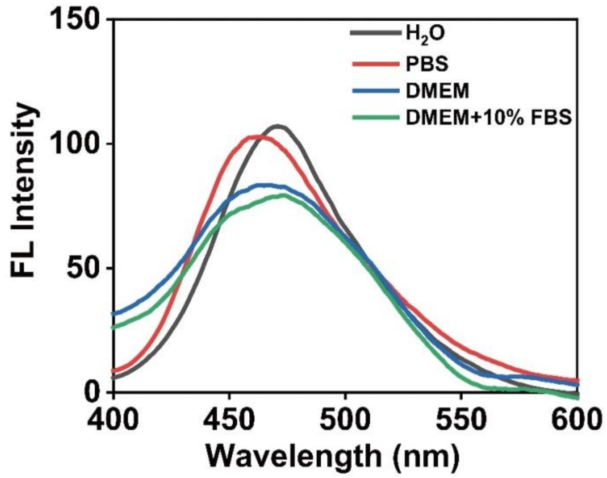

Supplementary Figure 16. Fluorescence spectra of CuAAC reaction catalyzed by Apt-Cu^{30} (5 μM) for 5 min at different biological conditions.

Supplementary 4. Activating the Fluorescence Probe by MApt-Cu^{30} inside Cell

Cellular culture

The MUC1-positive cell lines MCF-7 and A549, MUC1-negative cell lines HepG2 and MDA-MB-231, nucleolin-overexpressed cell line HeLa and the normal cell line HEK 293, and the normal cell line NIH 3T3 and RAW 264.7 were cultured with complete medium which was composed of high glucose DMEM, 10% FBS and 1% Penicillin-Streptomycin. The cells were placed in fresh medium every 3 days.

Cell viability assay

The cells with a density of 5000 cells/well were seeded in 96-well plate. After incubating for 24 h, the as-prepared MApt-Cu^{30} and AApt-Cu^{30} at indicated concentrations (0, 3.125, 6.25, 12.5, 25, 50 μM) were added, respectively. After culturing for another 24 h, cell medium was removed. And 100 μL of MTT solution was added to every well for additional 4 h. The medium of each well was replaced by 100 μL DMSO. The absorbance was measured by a Bio-Rad model-680 microplate reader at 570 nm. The cell viability was estimated according to the following equation: Cell Viability (%) = ($OD_{treated}/OD_{control}$) ×100%. $OD_{control}$ was obtained in the absence of nanocatalysts, whereas $OD_{treated}$ was obtained in the presence of nanocatalysts.

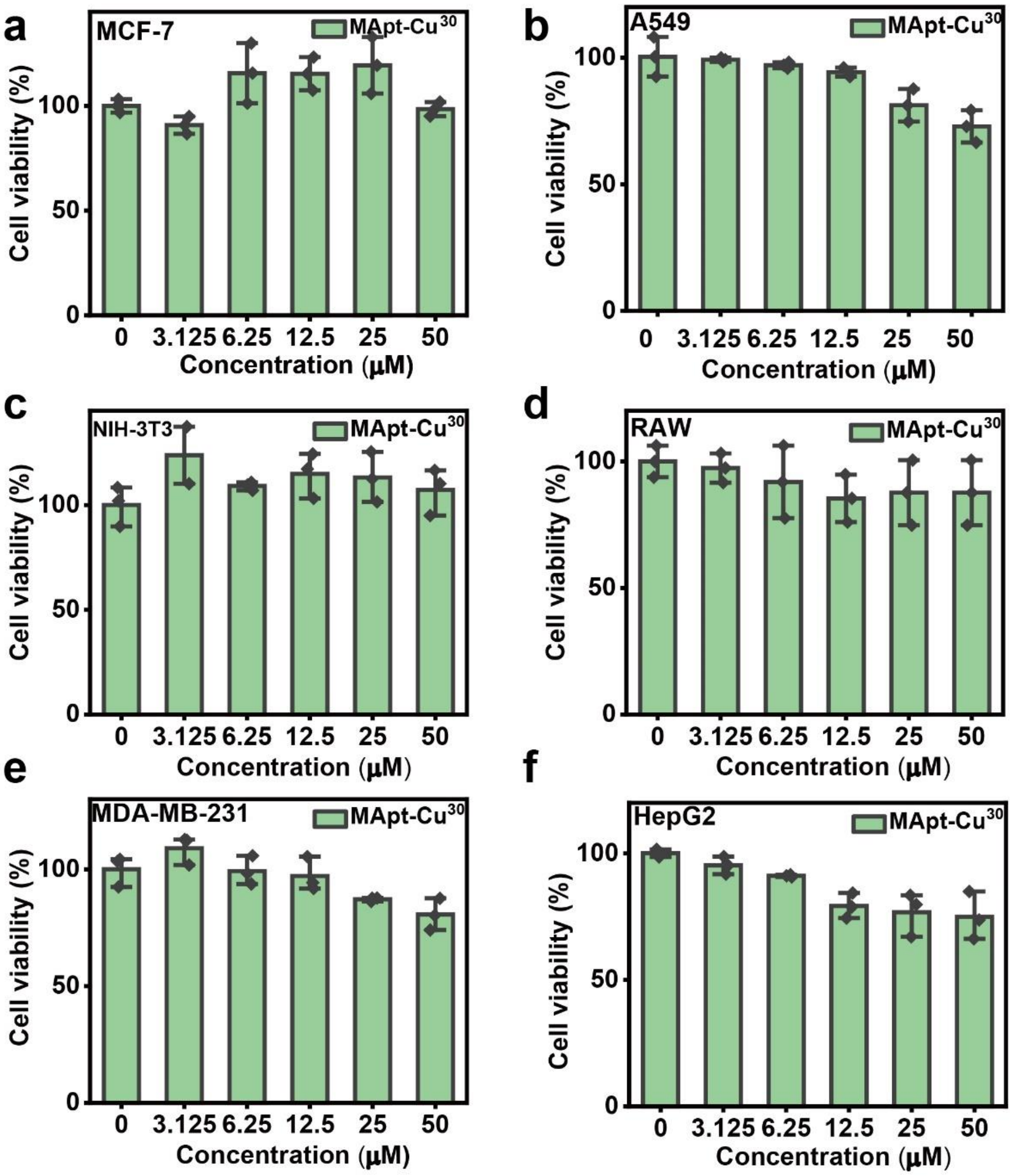

Supplementary Figure 17. MTT assay of MApt-Cu^{30} with different concentration on different cells. **a-f**) The cell viability of MCF-7, A549, NIH-3T3, RAW, MDA-MB-231 and HepG2 treated with MApt-Cu^{30}, respectively. Data were presented as mean ± SD (n = 3 independent experiments). Source data are provided as a Source Data file.

Cellular internalization of MApt-Cu^{30} and WA-Cu^{30}

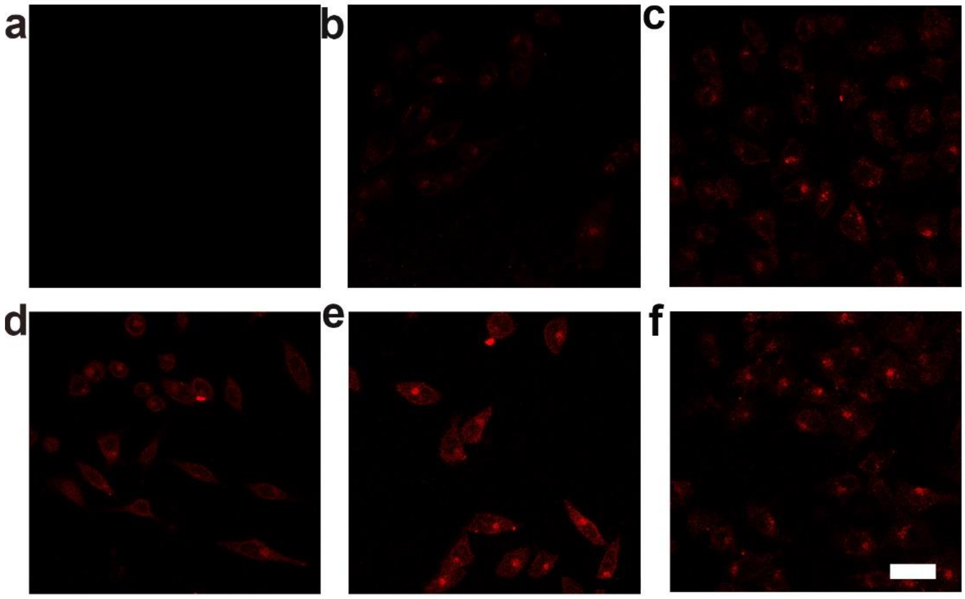

Supplementary Figure 18. Confocal microscopy images for tracking the cellular internalization of MApt-Cu^{30}. **a-f**) The cellular internalization of MApt-Cu^{30} for 0, 1, 2, 3, 4 and 6 h, respectively. Images are representative of three independent biological samples. Scale bars: 50 μm.

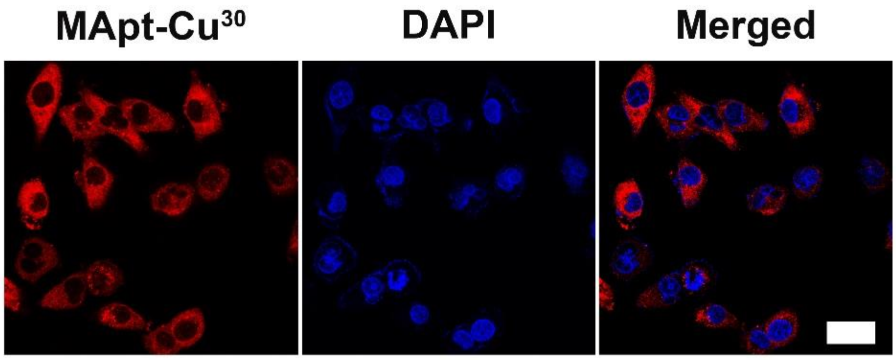

Supplementary Figure 19. The high-resolution confocal microscopy images of cellular uptake of MApt-Cu^{30} (red) at 4 h. DAPI (blue) was used to stain the cell nucleus. Images are representative of three independent biological samples. Scale bars: 50 μm.

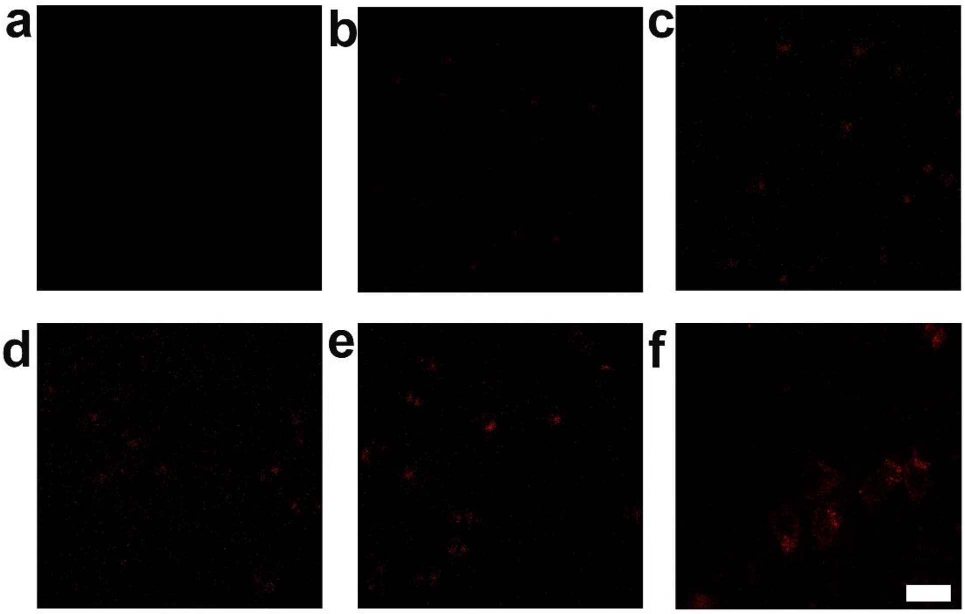

Supplementary Figure 20. Confocal microscopy images for tracking the cellular internalization of WA-Cu[30]. **a-f**) the cellular internalization of WA-Cu[30] for 0, 1, 2, 3, 4 and 6 h, respectively. Images are representative of three independent biological samples. Scale bars: 50 μm.

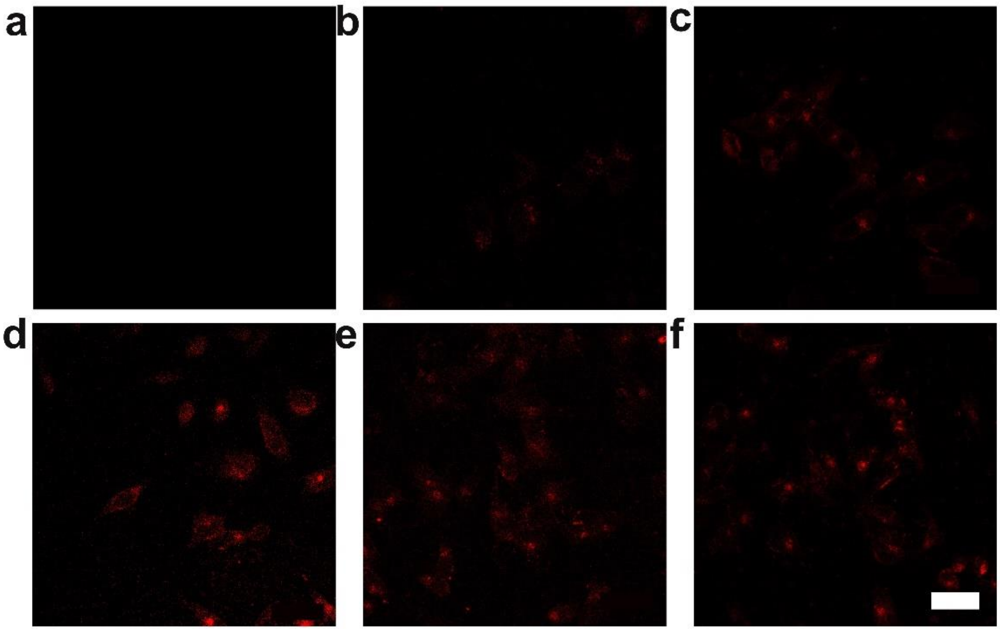

Supplementary Figure 21. Confocal microscopy images for tracking the cellular internalization of WA-Cu[30]. **a-f**) the cellular internalization of WA-Cu[30] for 0, 6, 12, 18, 24 and 30 h, respectively. Images are representative of three independent biological samples. Scale bars: 50 μm.

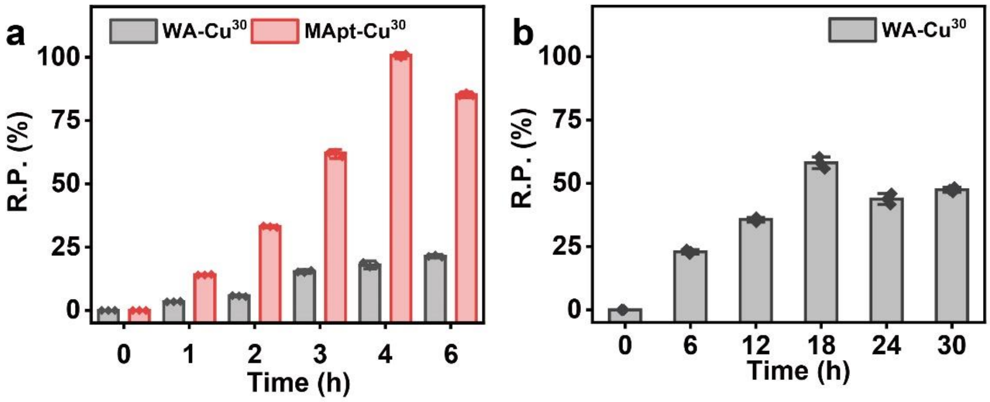

Supplementary Figure 22. The cellular uptake of nanocatalysts was tracked by ICP-MS. (**a**) The cellular uptake of MApt-Cu^{30} and WA-Cu^{30} for 0, 1, 2, 3, 4 and 6 h. Data were presented as mean ± SD (n = 3 independent experiments). (**b**) The cellular uptake of WA-Cu^{30} for 0, 6, 12, 18, 24, and 30 h. Data were presented as mean ± SD (n = 3 independent experiments). Source data are provided as a Source Data file.

To test the targeting capability of MApt-Cu^{30}, we analyzed the cellular uptake of CuNPs by ICP-MS via tracking Cu. The data showed that MApt-Cu^{30} could be efficiently uptaken in 4 h. However, the cellular uptake of WA-Cu^{30} was less than 25% in 6 h. The content of WA-Cu^{30} reached the maximum at 18 h. However, the count is only around half of cell uptake of MApt-Cu^{30} in four hours.

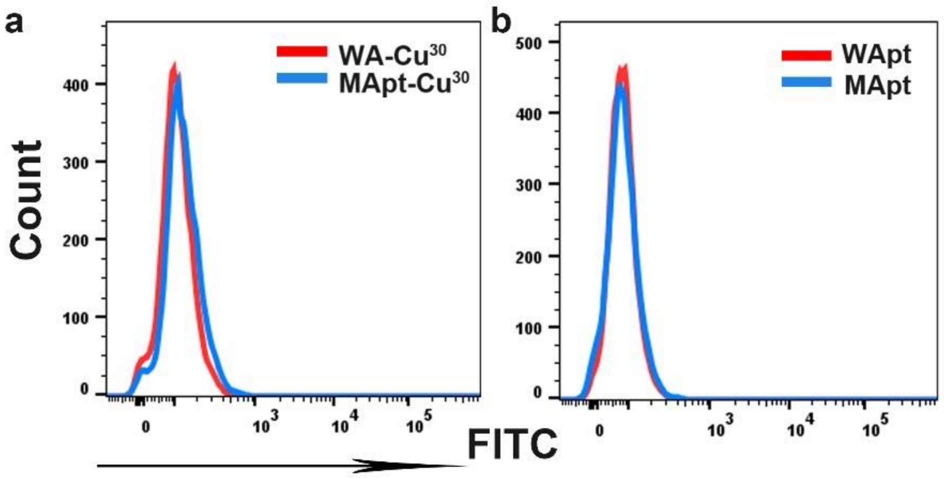

Supplementary Figure 23. (**a**) Flow cytometry assay of HEK293 cells treated with MApt-Cu^{30} and WA-Cu^{30}. (**b**) Flow cytometry assay of HEK293 cells treated with MApt-Cu and WA-Cu.

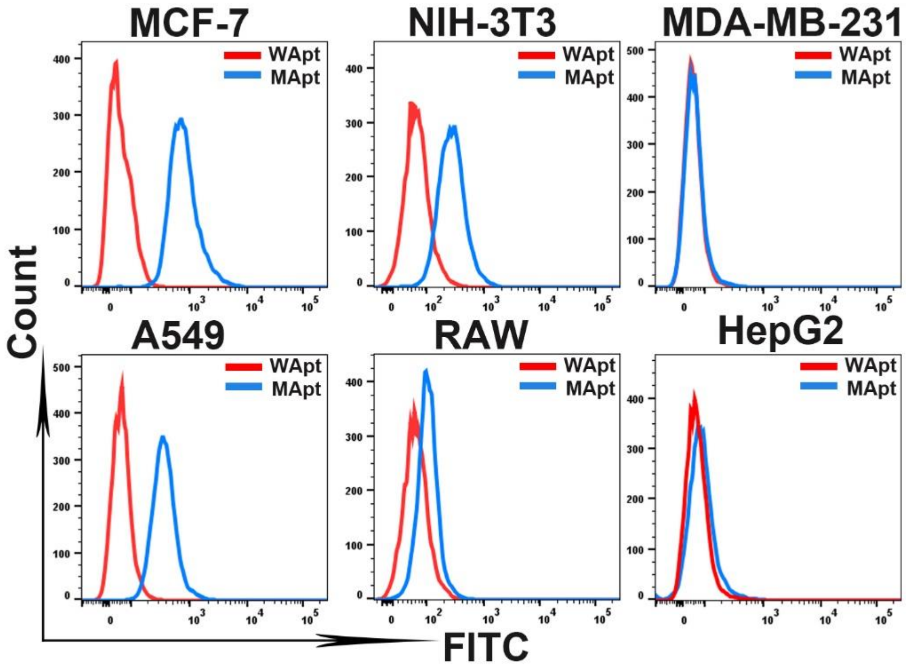

Supplementary Figure 24. Flow cytometry was performed to evaluate the binding of MApt and WApt with MCF-7, A549, NIH-3T3, RAW, MDA-MB-231 and HepG2 cells. FAM-labeled MApt and WApt were incubated with the above six kinds of cells for 30 minutes and washed. The red curves represent the results of DNA sequence without aptamer, and the blue curves represent the results of MUC1 aptamer.

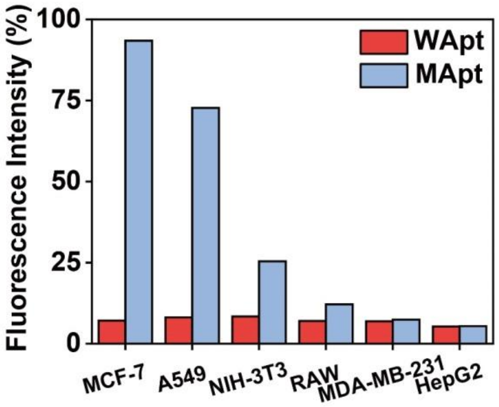

Supplementary Figure 25. Quantitative analysis of fluorescence intensity of MApt and WApt internalized into MCF-7, A549, NIH-3T3, RAW, MDA-MB-231 and HepG2 cells by flow cytometry. Event to record in the dashboard window was set to 10 000, and fluorescence intensities of the samples were automatically recorded.

MApt-Cu[30] mediated click reactions in living cells

The catalytic activity of the MApt-Cu[30] was investigated utilizing the coumarin "profluorophore" 3-azido-7-hydroxycoumarin **1** in living cells.

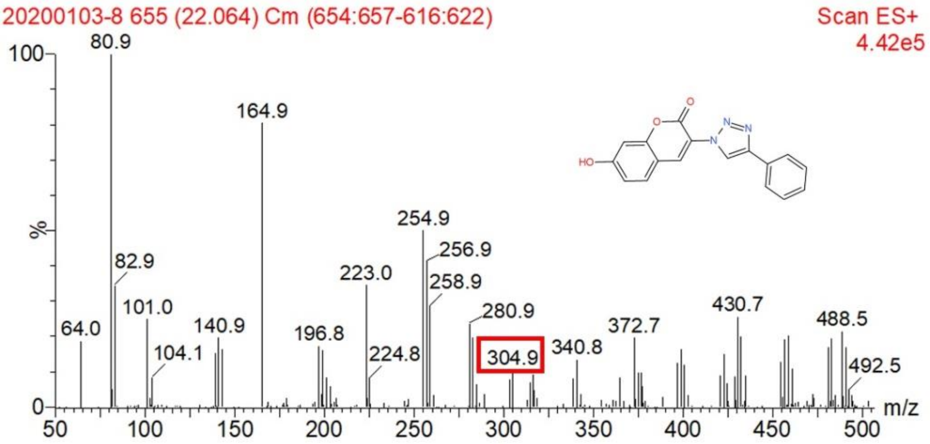

Supplementary Figure 26. LC-MS analysis of the cell lysate to confirm the presence of click coupling product **3**.

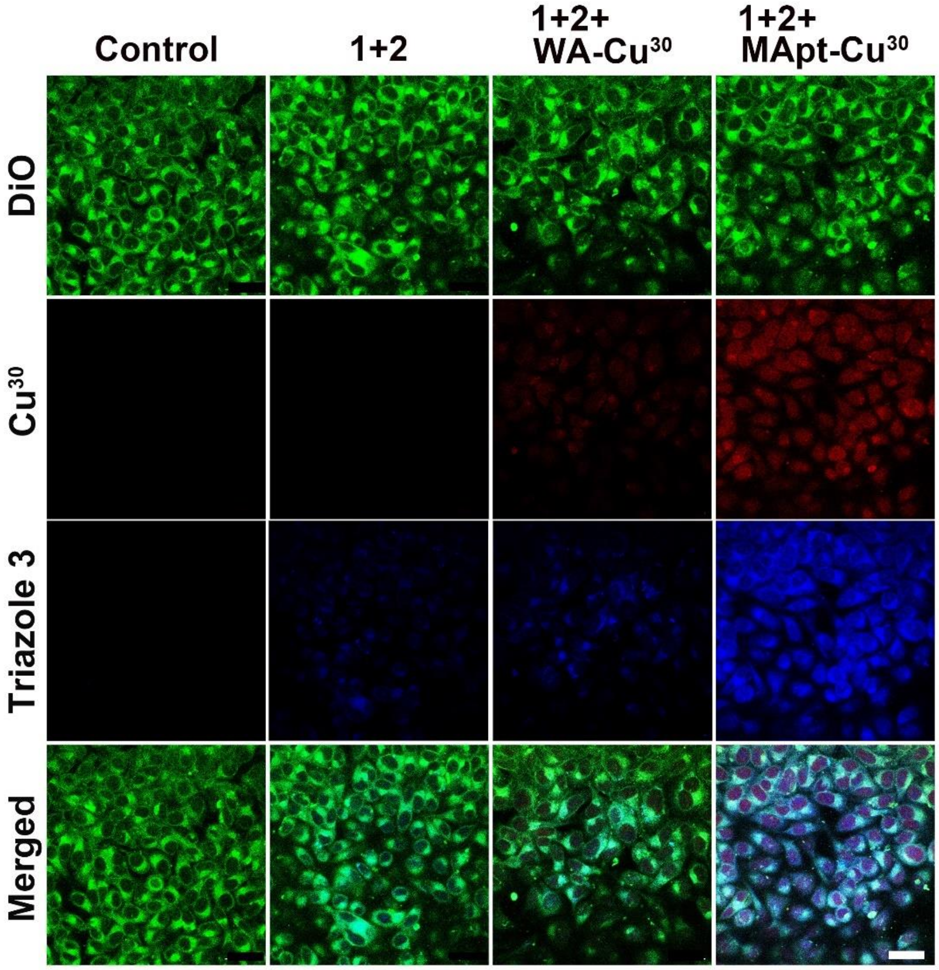

Supplementary Figure 27. Confocal microscopy images of A549 cells treated with **1**+**2**, **1**+**2**+ WA-Cu[30], and **1**+**2**+ MApt-Cu[30], respectively. Cytomembrane was stained with DIO (green), MApt-Cu[30] with autofluorescence of red and the product of triazole **3** (blue). Images are representative of three independent biological samples. Scale bars: 50 μm.

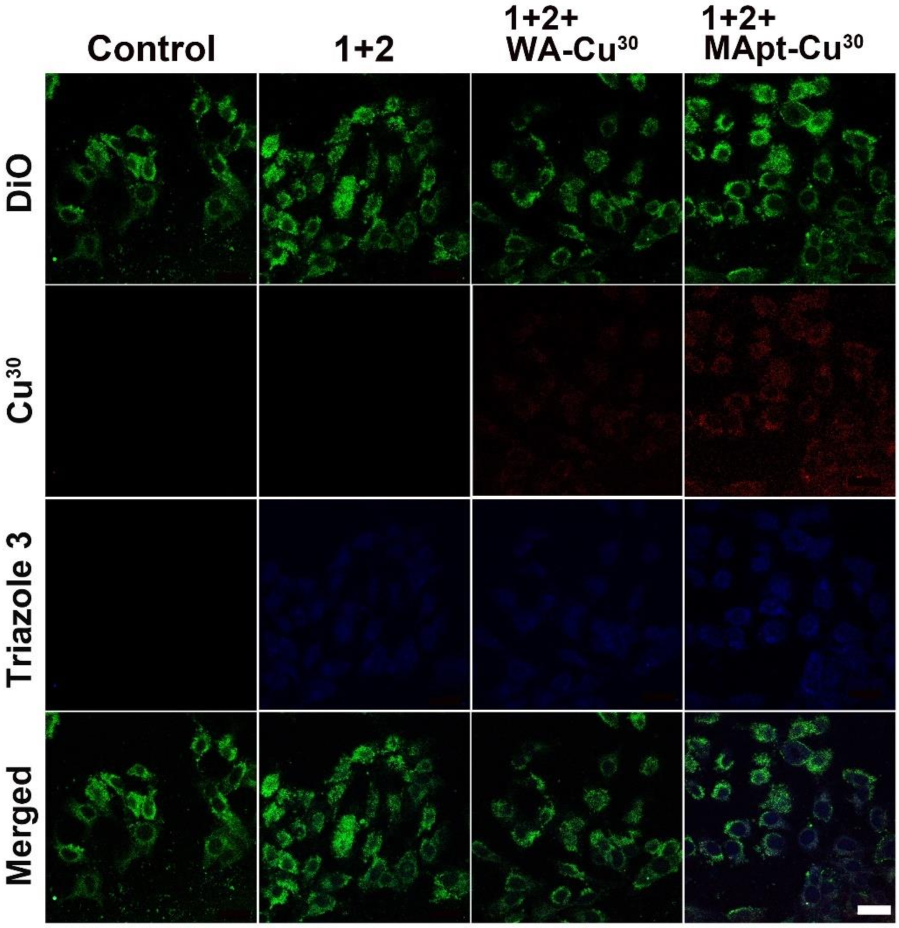

Supplementary Figure 28. Confocal microscopy images of HepG2 cells treated with **1**+**2**, **1**+**2**+ WA-Cu^{30}, and **1**+**2**+ MApt-Cu^{30}, respectively. Cytomembrane was stained with DIO (green), MApt-Cu^{30} with autofluorescence of red and the product of triazole **3** (blue). Images are representative of three independent biological samples. Scale bars: 50 μm.

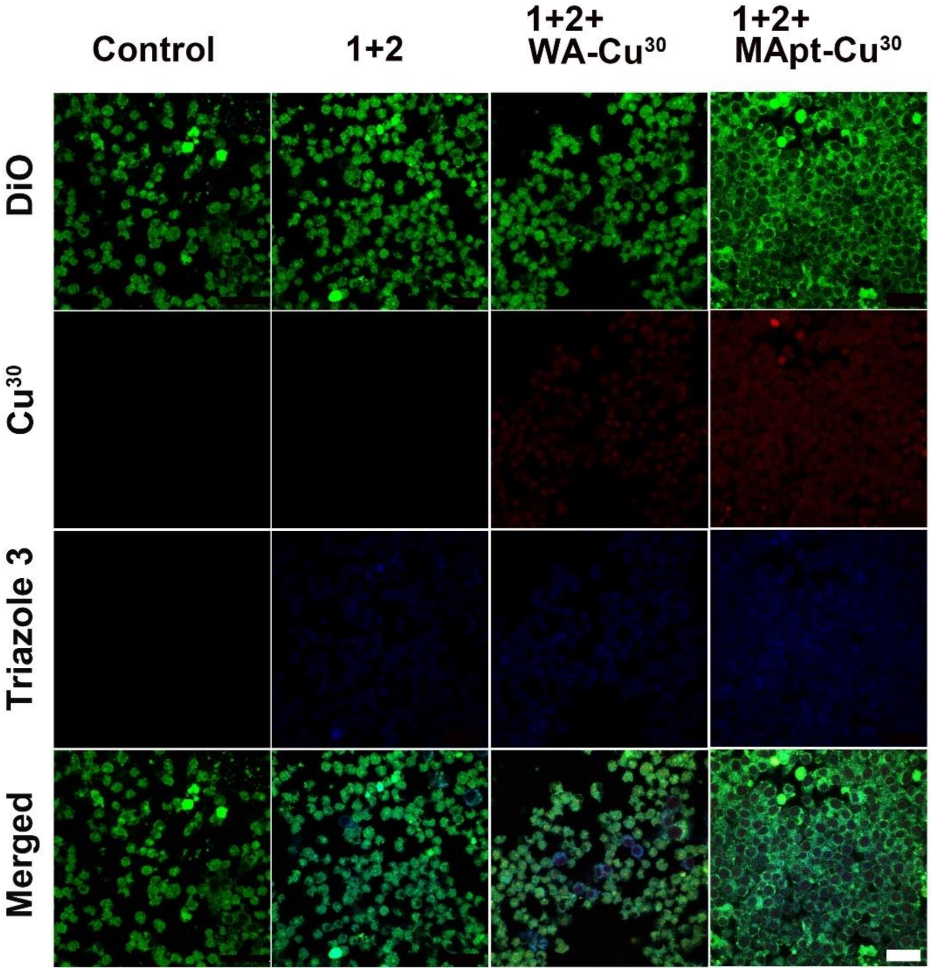

Supplementary Figure 29. Confocal microscopy images of RAW cells treated with **1**+**2**, **1**+**2**+ WA-Cu^{30}, and **1**+**2**+ MApt-Cu^{30}, respectively. Cytomembrane was stained with DIO (green), MApt-Cu^{30} with autofluorescence of red and the product of triazole **3** (blue). Images are representative of three independent biological samples. Scale bars: 50 μm.

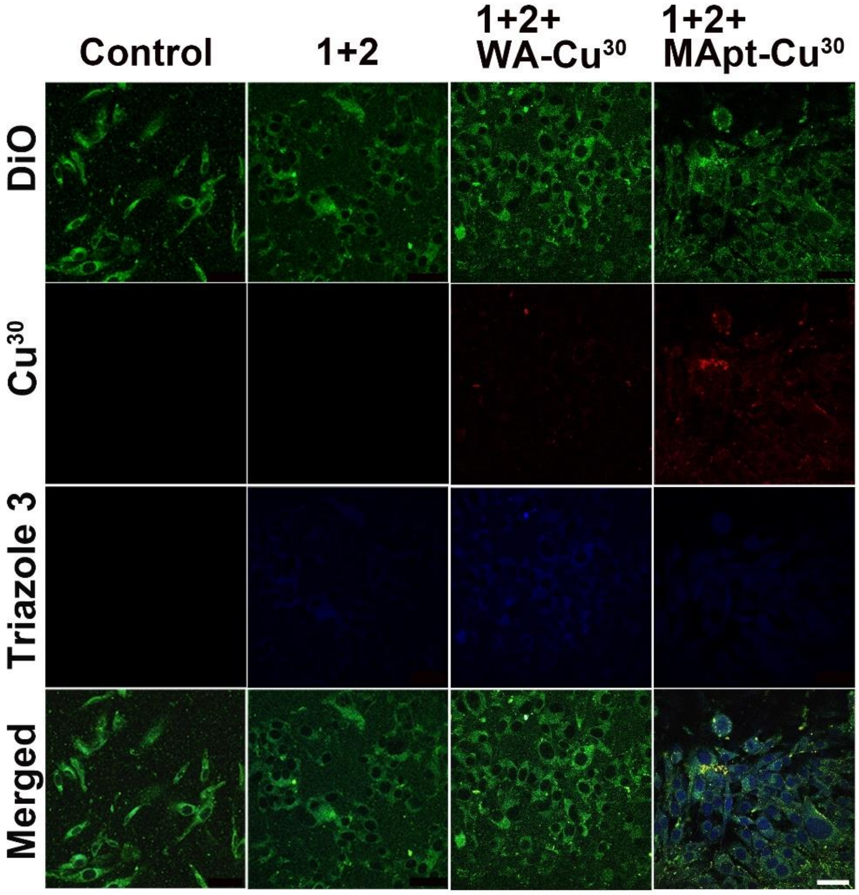

Supplementary Figure 30. Confocal microscopy images of NIH-3T3 cells treated with **1**+**2**, **1**+**2**+ WA-Cu^{30}, and **1**+**2**+ MApt-Cu^{30}, respectively. Cytomembrane was stained with DIO (green), MApt-Cu^{30} with autofluorescence of red and the product of triazole **3** (blue). Images are representative of three independent biological samples. Scale bars: 50 μm.

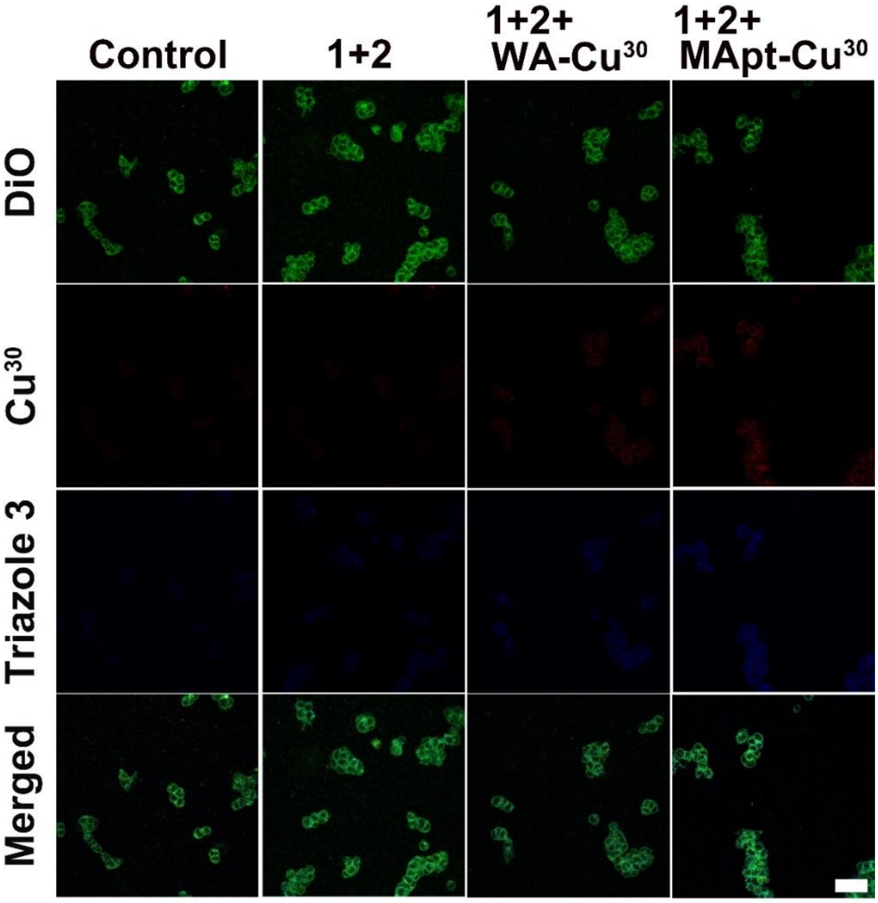

Supplementary Figure 31. Confocal microscopy images of HEK293 cells treated with **1**+**2**, **1**+**2**+WA-Cu^{30}, and **1**+**2**+MApt-Cu^{30}, respectively. Cytomembrane was stained with DIO (green), MApt-Cu^{30} with autofluorescence of red and the product of triazole **3** (blue). Images are representative of three independent biological samples. Scale bars: 50 μm.

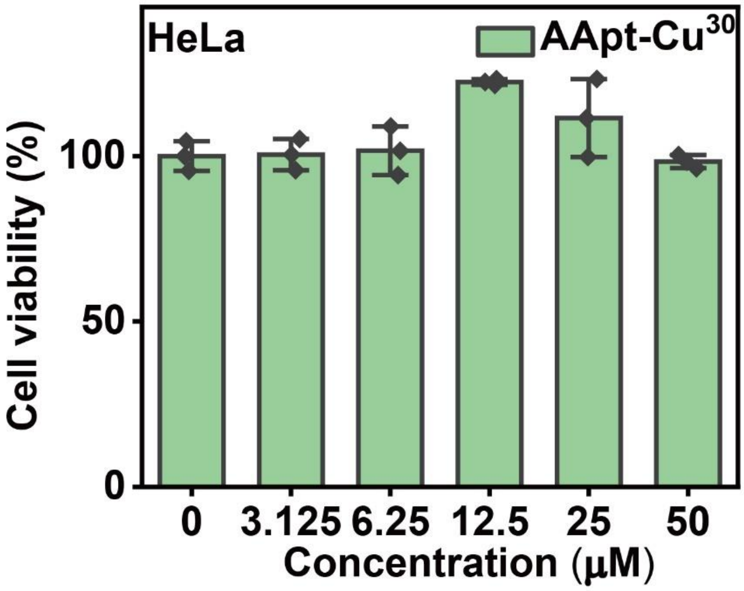

Supplementary Figure 32. MTT assay of AApt-Cu^{30} with different concentration on HeLa cells. Data were presented as mean ± SD (n = 3 independent experiments). Source data are provided as a Source Data file.

Fluorescence imaging for studying the catalytic activity of AApt-Cu^{30} in living cells

HeLa cells were planted in 24-well plates overnight. Then, AApt-Cu^{30} was added and cultured for 4 h. After washing with PBS, cells were incubated with azide coumarin, **1** (10 mM in DMSO) and alkyne, **2** (10 mM in DMSO) with a final concentration of 10 μM for 12 h. Subsequently, the cells were washed with PBS. The fluorescence images were collected by OLYMPUS-BX51 microscopes with U-25ND25 filter. AS1411 aptamer-FAM was used instead of AS1411 aptamer for the preparation of AApt-Cu^{30} to detect it fluorescence.

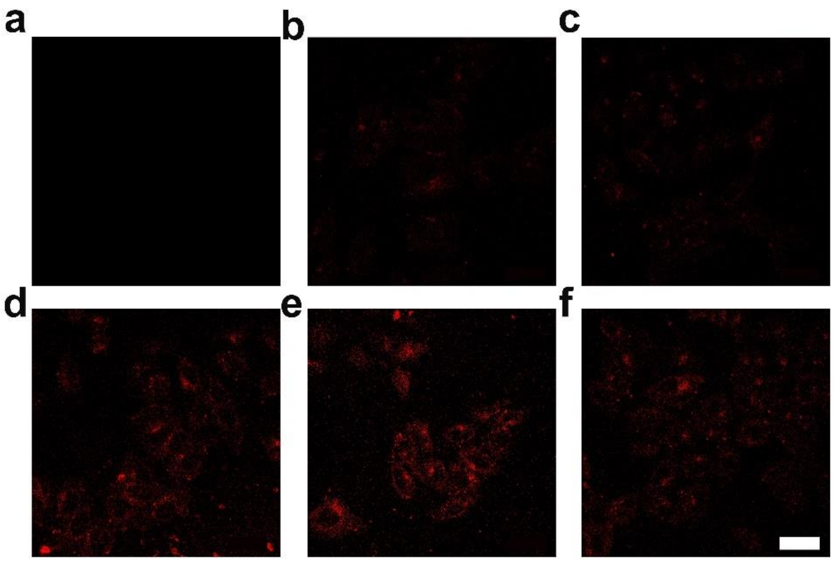

Supplementary Figure 33. Confocal microscopy images for tracking the cellular internalization of AApt-Cu^{30}. **a-f**) the cellular internalization of AApt-Cu^{30} for 0, 1, 2, 3, 4 and 6 h, respectively. Images are representative of three independent biological samples. Scale bars: 50 μm.

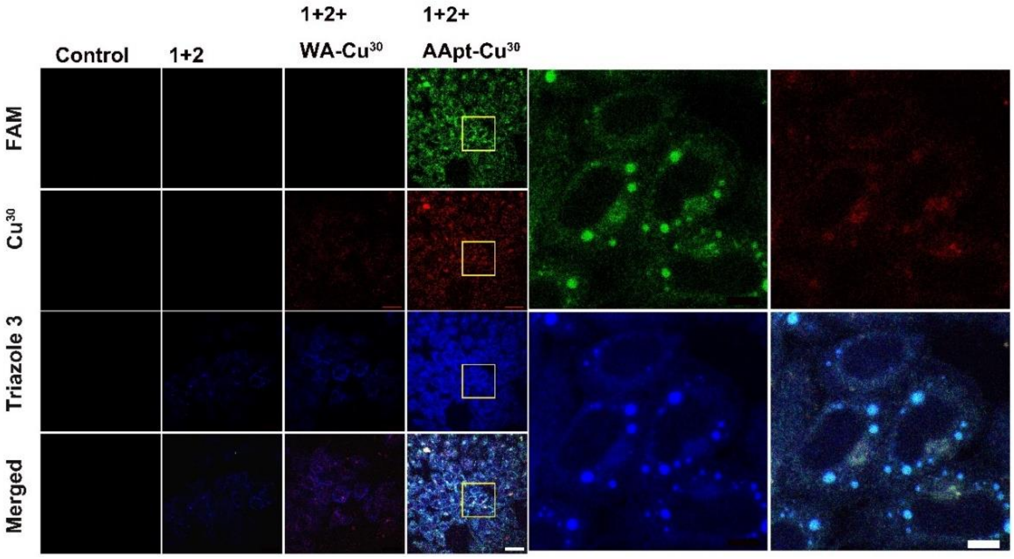

Supplementary Figure 34. Confocal microscopy images of Hela cells treated with **1**+**2**, **1**+**2**+WA-Cu^{30}, and **1**+**2**+AApt-Cu^{30}, respectively. Cytomembrane was stained with DIO (green), AApt-Cu^{30} with autofluorescence of red and the product of triazole **3** (blue). Images are representative of three independent biological samples. Scale bars: 50 μm. (High resolution images of corresponding HeLa cells treated with **1**+**2**+AApt-Cu^{30}. Scale bars: 10 μm.)

Supplementary 5. Synthesis of Resveratrol Analogue Catalyzed by MApt-Cu[30] in Living Cells.

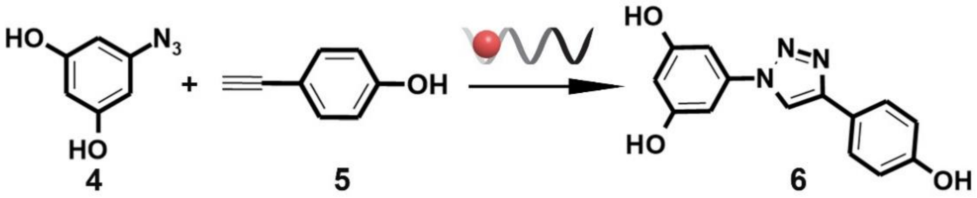

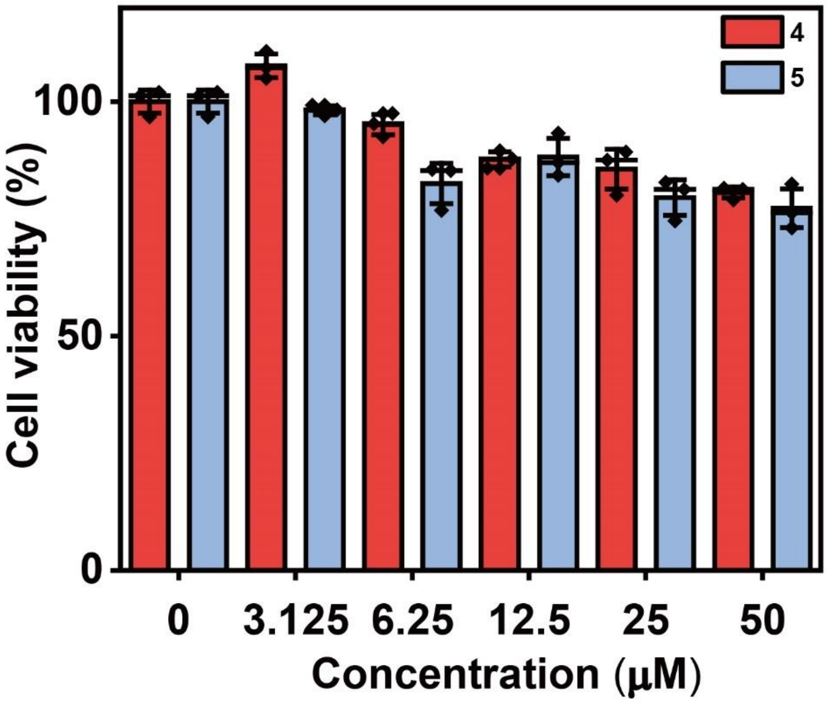

Supplementary Figure 35. MTT assays of MCF-7 cells treated with different concentrations of **4** and **5**. Data were presented as mean ± SD (n = 3 independent experiments). Source data are provided as a Source Data file.

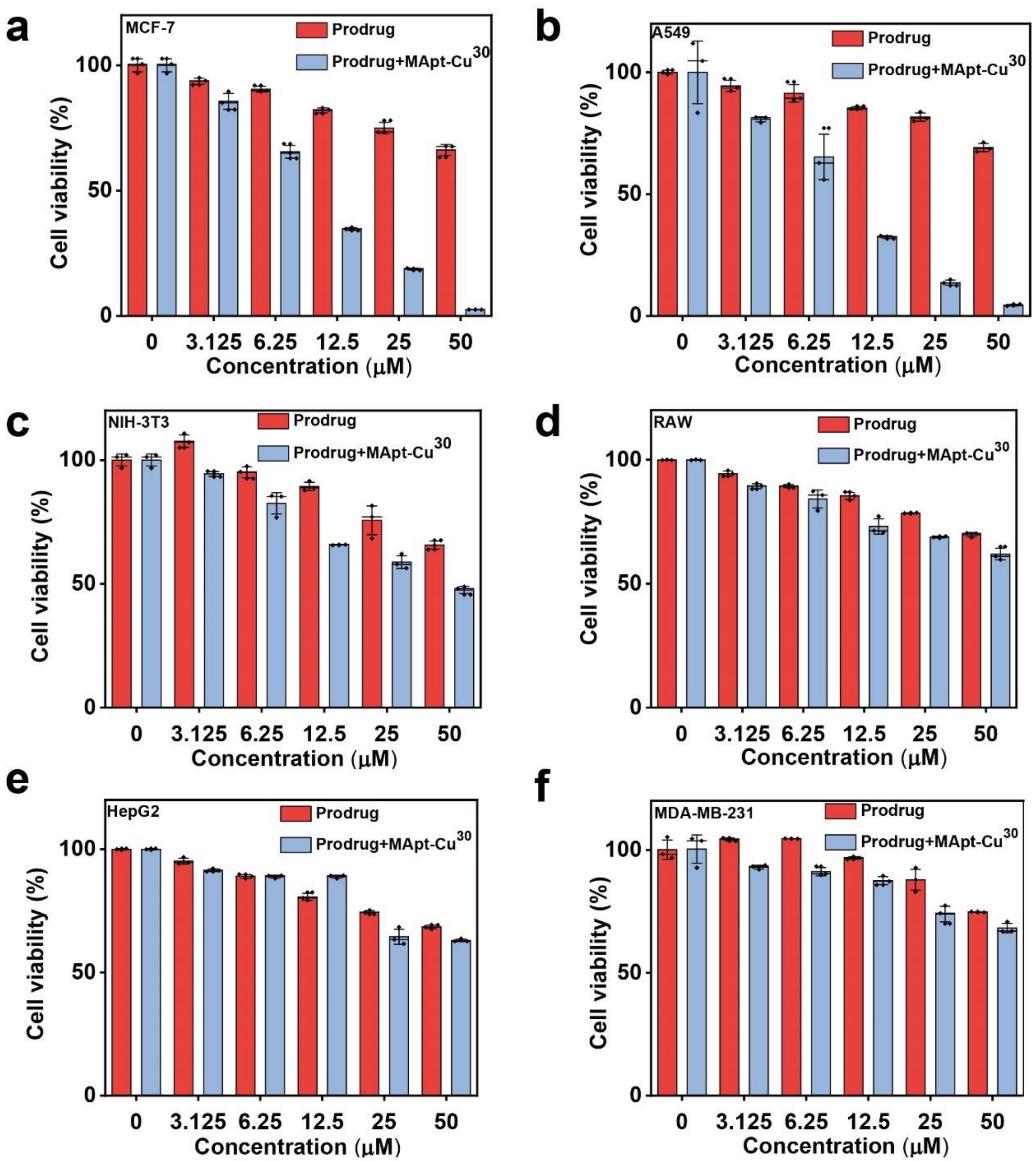

Supplementary Figure 36. MTT assays of different cells treated with different concentrations of prodrugs **4** and **5** without and with MApt-Cu^{30}. **a-f**) with MCF-7, A549, NIH-3T3, RAW, HepG2 and MDA-MB-231 cells, respectively. Data were presented as mean ± SD (n = 3 independent experiments). Source data are provided as a Source Data file.

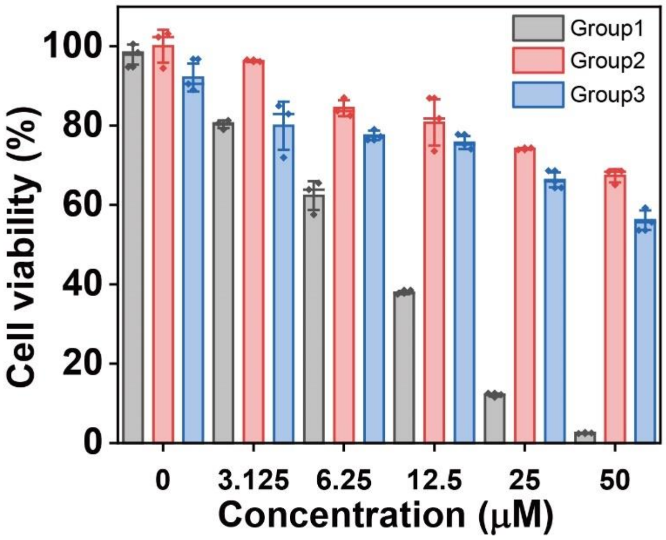

Supplementary Figure 37. MTT analysis of prodrug activation catalyzed by different DNA-templated CuNPs in MCF-7. Group 1: MApt-Cu^{30}, Group 2: MApt-Cu^{30}-block, Group 3: mutMApt-Cu^{30}. Error bars represented the standard deviation (n = 3 independent experiments). Data were presented as mean ± SD. Source data are provided as a Source Data file.

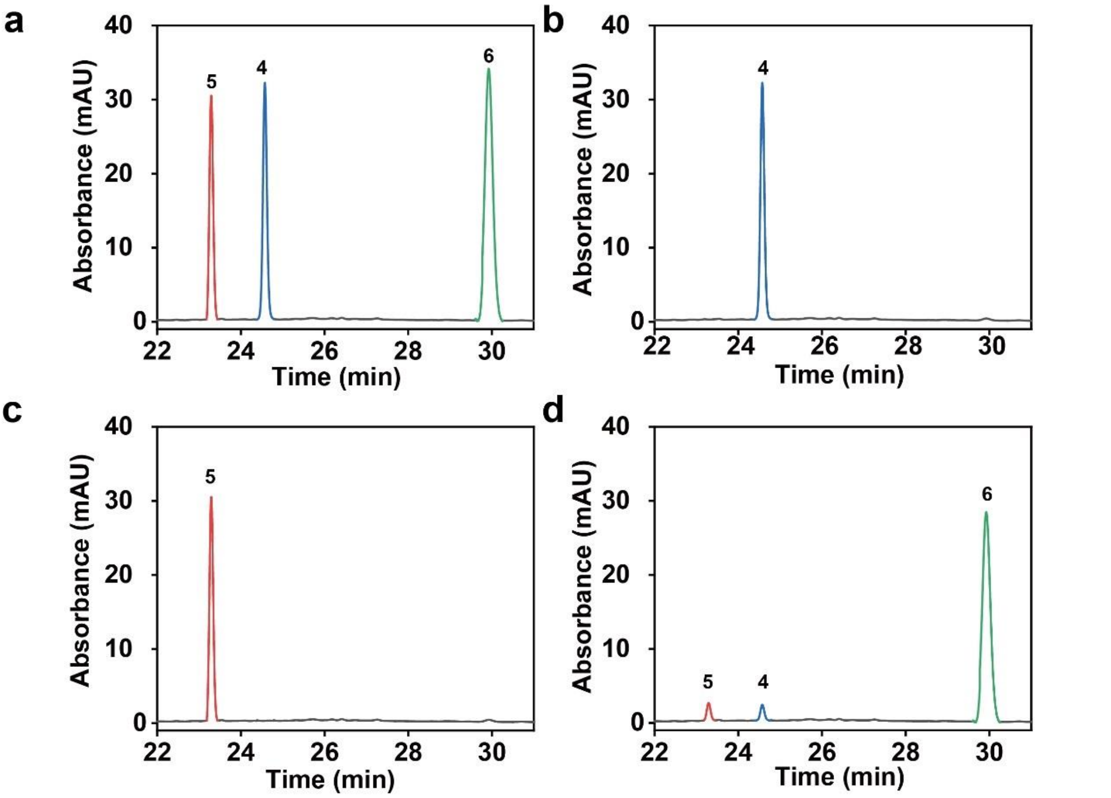

Supplementary Figure 38. HPLC analysis of cell lysate after reaction catalyzed by MApt-Cu[30]. A-C) HPLC chromatograms of the standard examples of **4**, **5**, and **6**. The retention times of **4**, **5**, and **6** were 24.57, 23.29, and 29.92 min, respectively. D) HPLC chromatogram of the cell lysate confirmed the presence of click coupling product **6**.

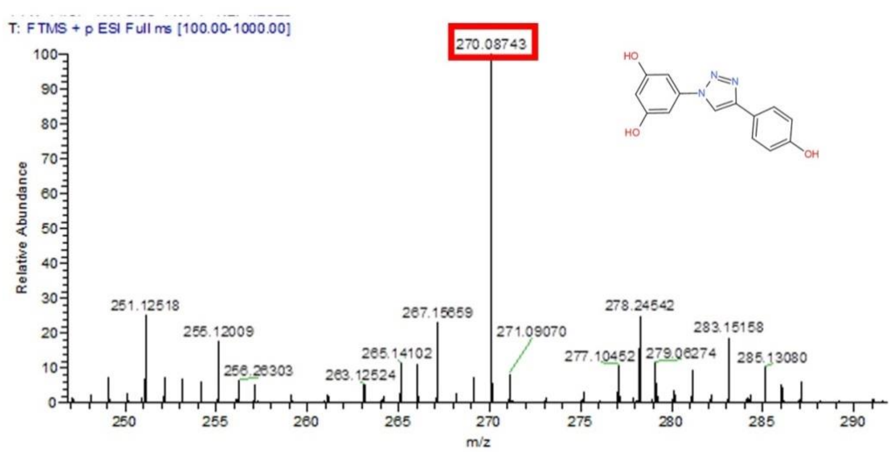

Supplementary Figure 39. The LC-MS analysis of the cell lysate confirmed the presence of click coupling product **6**.

Supplementary 6. Targeted prodrug activation *in vivo* for Tumor Therapy

CuAAC reaction catalyzed by MApt-Cu^{30} in *C. elegans* model.

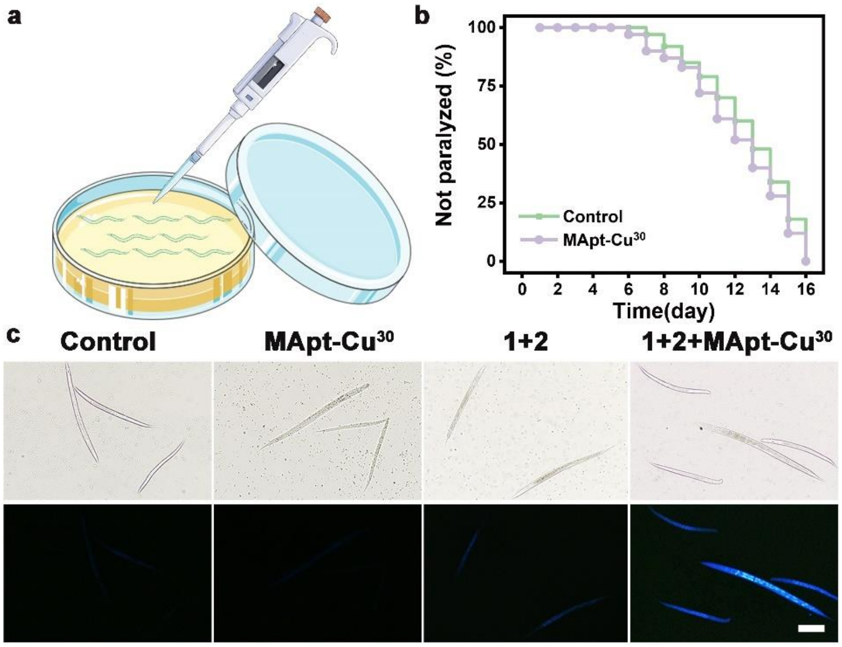

Supplementary Figure 40. (A) Schematic illustration of reaction setup. B) Kaplan-Meier survival curves of worms fed with and without MApt-Cu^{30}. (C) Bright-field and fluorescence images (blue channel) of worms treated with **1**+**2**, MApt-Cu^{30}, and **1**+**2**+MApt-Cu^{30}, respectively. Images are representative of three independent biological samples. Scale bars: 100 μm.

Animal Experiments.

Healthy *BalB/c-nu* mice (14~16 g) were purchased from the Laboratory Animal Center of Jilin University (Changchun, China). MCF-7 cells and MDA-MB-231 (2×10^5 cells) were subcutaneously injected into athymic nude mice to establish the tumor bearing mice model,[10,11] respectively. All animal handling procedures were according to the guidelines of the Regional Ethics Committee for Animal Experiments.

The biocompatibility of the MApt-Cu^{30} in mice.

Hemolysis test of MApt-Cu^{30} was first carried out. 1 mL whole blood was collected in tubes containing Li-heparin from the Orbital venous of *BalB/c-nu* mice. Then, 1 mL blood was mixed with proper amount of 1×PBS and centrifuged for 5 min at 1118 g. The supernatant was removed. Repeat 3-4 times until the supernatant became colorless and transparent. The precipitated erythrocytes were dispersed in 1×PBS to get erythrocyte suspension. The erythrocyte suspension diluted with ultrapure water was placed in tube 1 as positive control. The erythrocyte suspension diluted with 1×PBS was placed in tube 2 as negative control. In tube 3-7, MApt-Cu^{30} with final concentrations of 3.125, 6.25, 12.5, 25, 50 μM were added to the erythrocyte suspension (from left to right). These tubes were incubated for 8 h at room temperature. The hemolysis phenomena were observed and recorded. The specific absorption of hemoglobin at 540 nm was recorded and analyzed. The hemolysis rate (HR %) was calculated according to the following equation:

$$HR\% = (A_{MApt\text{-}Cu^{30}} - A_{NC}) \times 100\% / (A_{PC} - A_{NC}), \quad (1)$$

where $A_{MApt\text{-}Cu^{30}}$, A_{PC} and A_{NC} are the absorbance of the sample, the positive control, and the negative control, respectively.

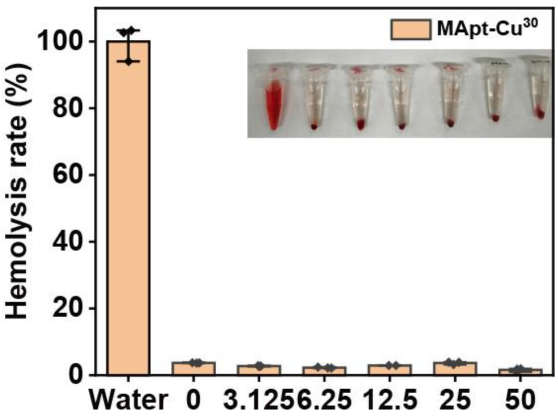

Supplementary Figure 41. Hemolysis rate (HR%) of MApt-Cu^{30} and photograph of hemolysis test (insert). Data were presented as mean ± SD (n = 3 independent experiments). Source data are provided as a Source Data file.

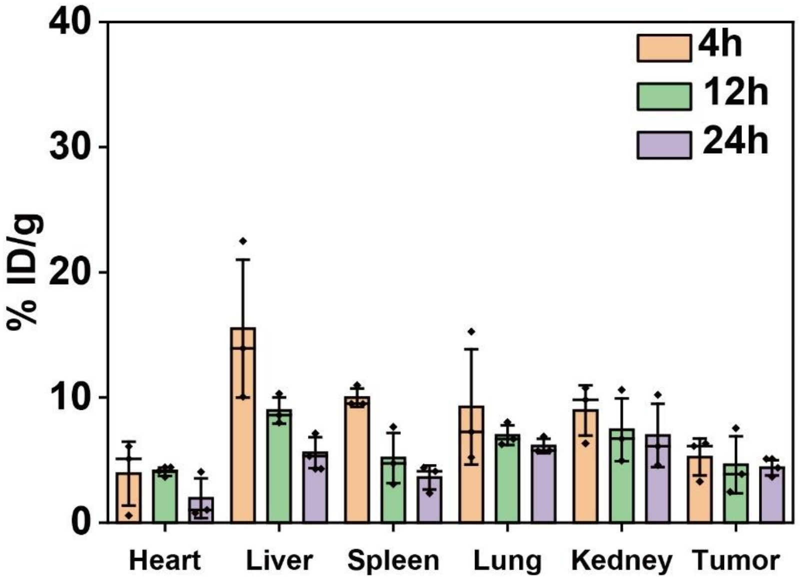

Supplementary Figure 42. The biodistribution of catalyst in MCF-7 tumor-bearing mice at different time points using ICP-MS analysis. Data were presented as mean ± SD (n = 3 independent experiments). Source data are provided as a Source Data file.

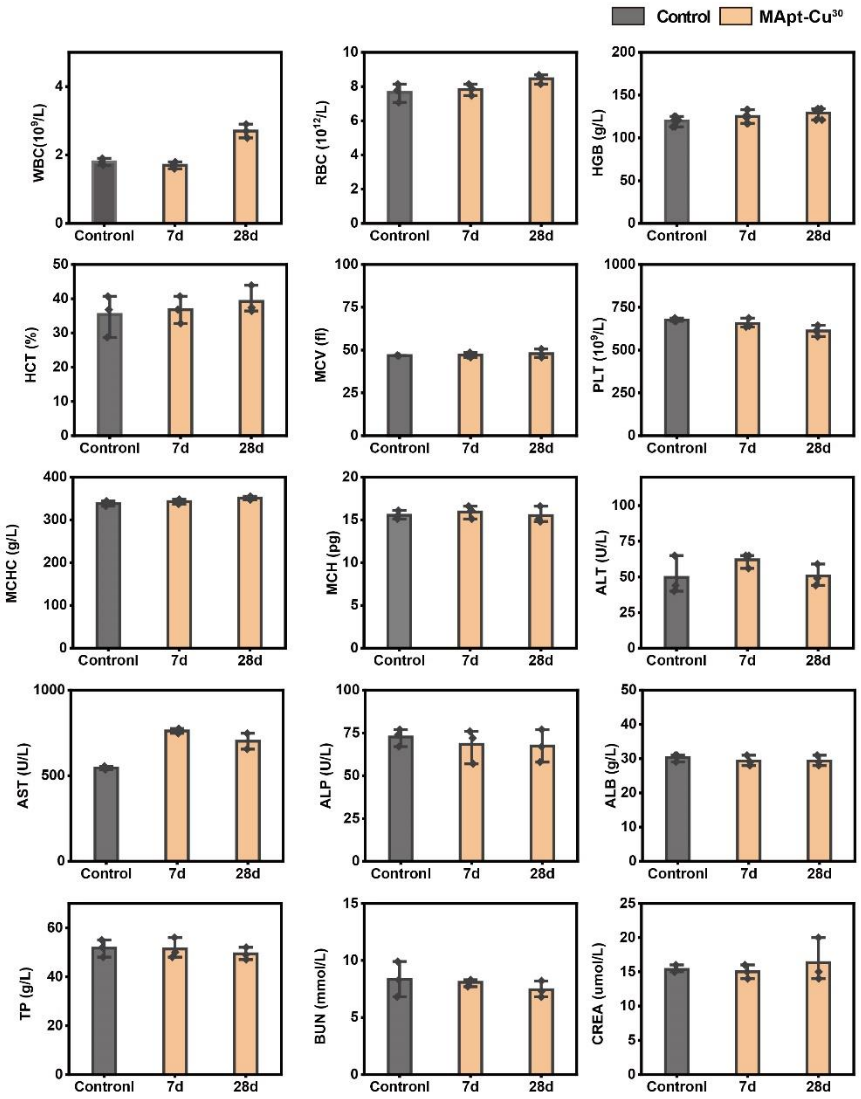

Supplementary Figure 43. The hematological parameters and blood biochemical levels of the mice after injection of PBS or MApt-Cu[30] collected on 7, 28 days post injection. Data were presented as mean ± SD (n = 3 independent experiments). Source data are provided as a Source Data file.

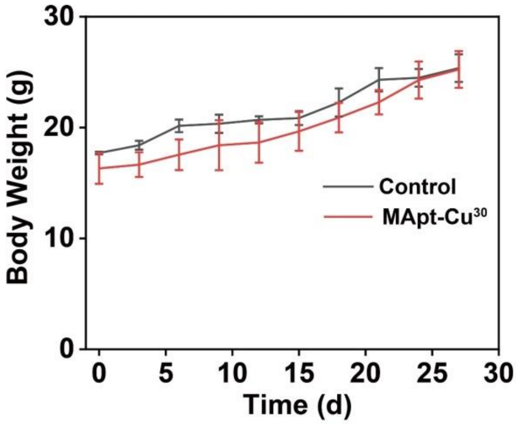

Supplementary Figure 44. Body weights of mice treated with PBS or MApt-Cu^{30}, recorded every three days. Data were presented as mean ± SD (n = 4 independent experiments). Source data are provided as a Source Data file.

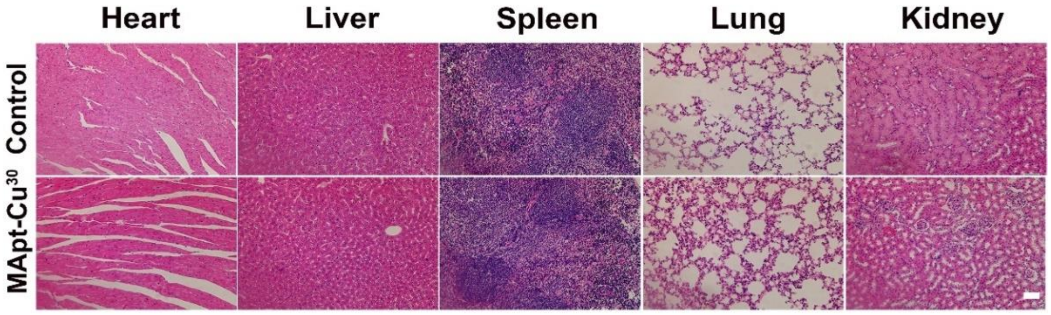

Supplementary Figure 45. H&E stained images of major organs 28 days after the injection of MApt-Cu^{30}. No abnormalities were observed in major organs compared with those of control group. Images are representative of three independent biological samples. (Scale bar= 50 μm)

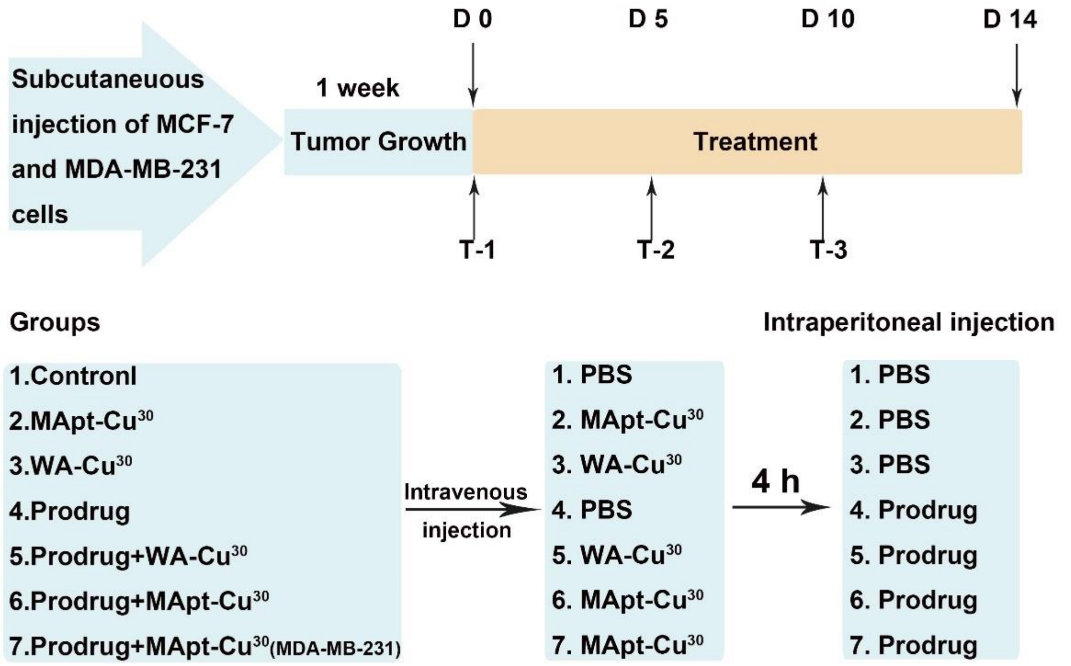

Supplementary Figure 46. Experimental design for the CuAAC reaction *in vivo* test.

Pharmacokinetic analysis of prodrug 4, prodrug 5 and product 6

21 BalB/c mice (female, 14~16 g) were randomly divided into 7 groups. Briefly, the mice were fasted for 12 hours before the administration and water was taken freely. According to different groups, the prodrug **5** was administered by intraperitoneal injection with 20 mg kg^{-1} concentration of equal volume (50 ul). After 0, 10, 15, 30, 60, 120, 240 minutes of administration, blood was collected from the mice posterior orbital venous cluster and then placed in a centrifugal tube containing heparin sodium. The plasma was separated by centrifugation at 1030 g for 10 minutes.[1] Prodrug **5** was analyzed and quantified via HPLC. Prodrug **4** was analyzed and quantified using the same way. For product **6,** 4 hours after intravenous injection of nanocatalyst, prodrug 4 and 5 were injected intraperitoneally. The blood was analyzed according to the above experimental steps.[12]

Supplementary Table 5. Quantitative analysis of prodrug 4, prodrug 5 and product 6 in blood.

Time (min)	Percentage of Product 6 (%)	Standard Deviation 1	Percentage of Prodrug 4(%)	Standard Deviation 2	Percentage of Prodrug 5 (%)	Standard Deviation 3
0	0	0	0	0	0	0
10	0.3875	0.34641	3.4	1.06066	5.325	5.37401
15	0.45	0.70711	9.675	4.52548	9.4375	3.11127
30	0.7	1.50444	11.2875	0.70711	11.4125	0.70711
60	0.3125	0.65574	3.9	5.86899	4.4625	5.16188
120	0.0625	0.23094	3.5125	5.65685	4.025	0.70711
240	0	0	3.6	5.16188	3.4	0.49497

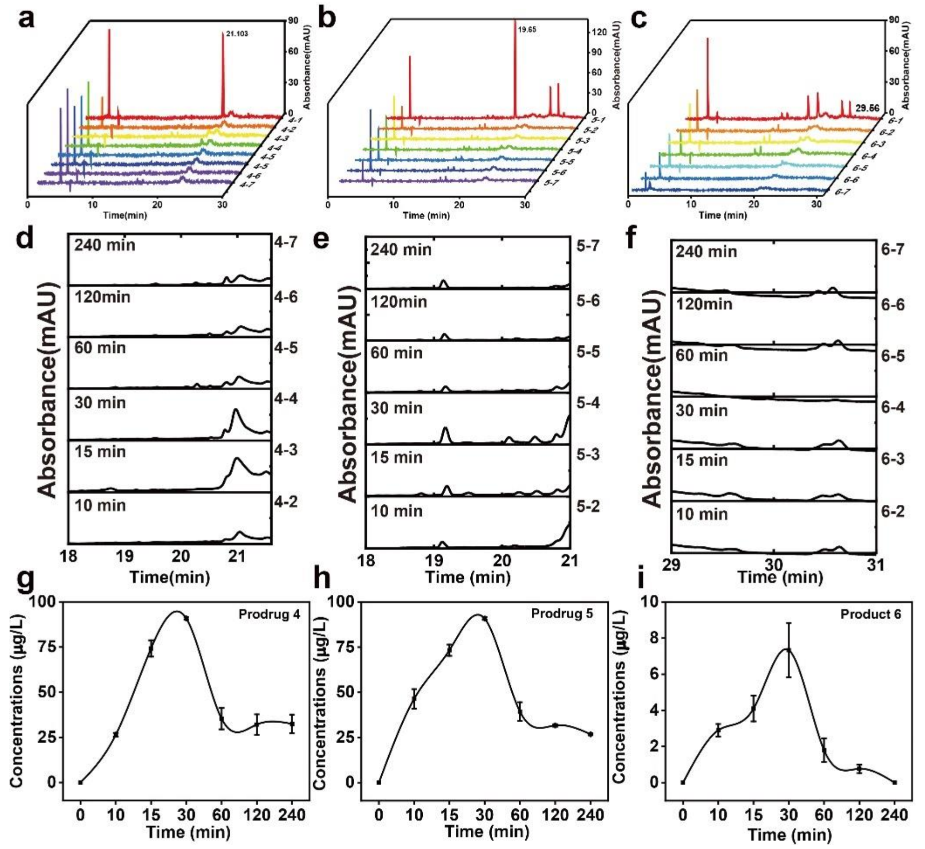

Supplementary Figure 47. HPLC analysis of prodrug **4**, prodrug **5** and product **6** in blood at different reaction time (0-240 min). **a-c**) HPLC chromatogram of prodrug **4**, prodrug **5** and product **6** at 0, 10, 15, 30, 60, 120, and 240 min in blood, respectively. **d-e**) The enlarged interval of HPLC analysis of prodrug **4**, prodrug **5** and product **6** at

0, 10, 15, 30, 60, 120, and 240 min, respectively. **g-i**) The concentration of prodrug **4**, prodrug **5** and product **6** in blood at 0, 10, 15, 30, 60, 120, and 240 min, respectively. Error bars represented the standard deviation (n = 3 independent experiments). Data were presented as mean ± SD. Source data are provided as a Source Data file.

Supplementary 7. Synthesis of Chemical Substrate Molecules

3-Azido-7-hydroxy-chromen-2-one (3-Azido-7-hydroxycoumarin) (1)

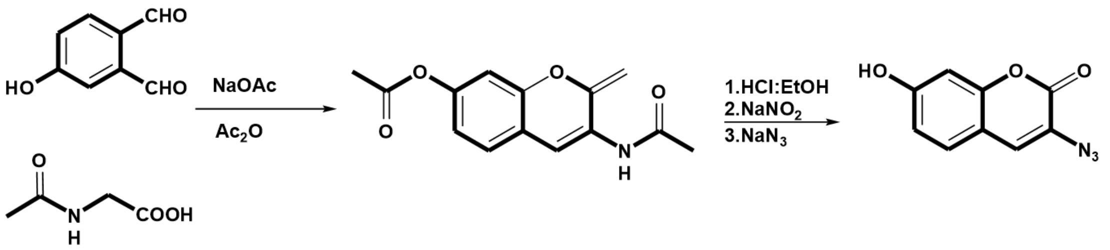

N-acetylglycine (20 mmol), 2, 4-dihydroxybenzaldehyde (20 mmol), anhydrous sodium acetate (60 mmol) were dissolved in 100 ml of acetic anhydride. Then the mixture refluxed under magnetic stirring. After 4 h, the mixture was put on the ice to obtain the yellow solid. The resulting solid was gathered using filtration and washed by ice water before it was refluxed in a solution of conc. HCl and CH_2OH (2:1) for 1 h. Then 40 mL ice water and $NaNO_2$ (40 mmol) were sequentially added into the solution in an ice bath. After stirring about 5 minutes, NaN_3 (60 mmol) was added in portions. After 15 minutes, the product was separated by filtration, washed with ultrapure water, and dried to obtain a brown solid; the yield was about 54 %. The product was pure enough for further reactions.[13]

^{1}H NMR (600 MHz, DMSO-*d6*) δ 10.55 (s, 1H), 7.61 (s, 1H), 7.50 (d, J = 8.5 Hz, 1H), 6.80-6.84 (dd, J= 8.5, 2.2 Hz, 1H), 6. 64 (d, J = 2.1 Hz, 1H).

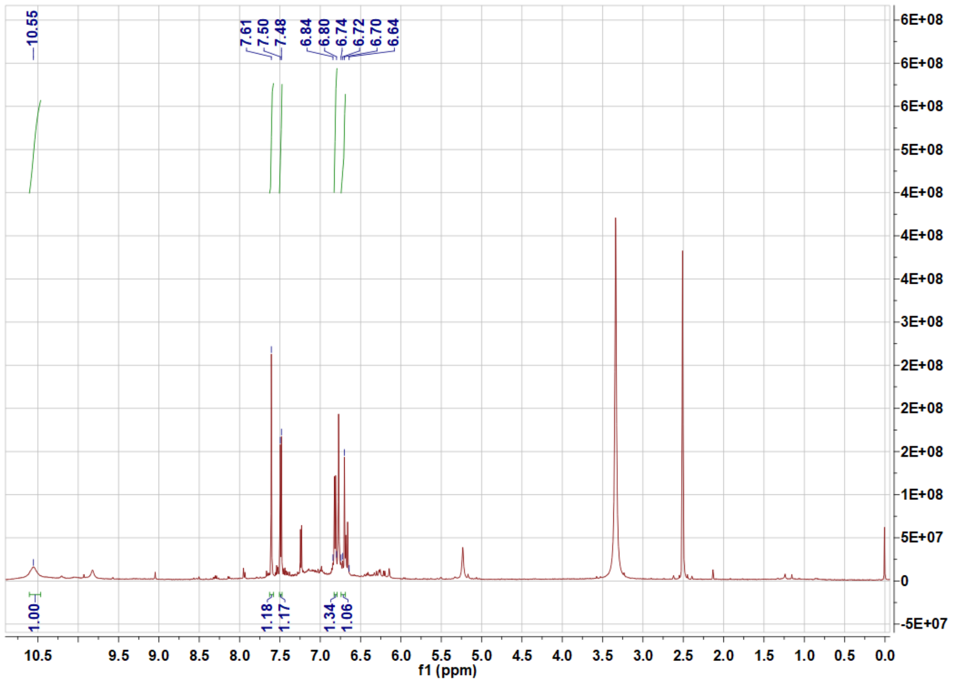

Supplementary Figure 48. ^{1}HNMR spectrum of 3-Azido-7-hydroxy-chromen-2-one (3-Azido-7-hydroxycoumarin), **1**. (H_2O δ=3.3; DMSO-*d6* δ=2.5).

7-hydroxy-3-(4-phenyl-1H- [1,2,3] triazole-1-yl)-coumarin (3)

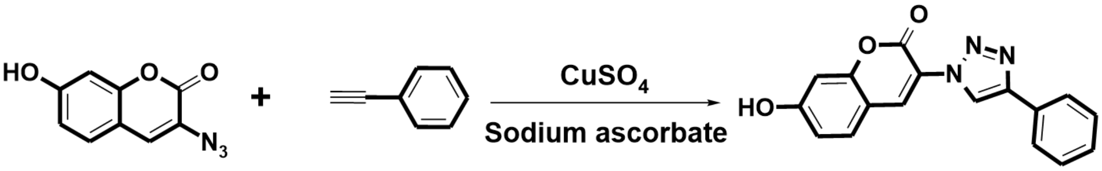

Phenylacetylene (0.17 mmol) and 3-Azido-7-hydroxycoumarin (0.17 mmol) were mixture in H_2O and CH_2OH (1:1, 5 mL), sodium ascorbate (0.034 mmol) of freshly prepared 1 M solution in water was added, followed by the addition of $CuSO_4 \cdot 5H_2O$ (0.0085 mmol) 7.5 % in water. The mixture reacted under vigorous stirring in the dark. After 10 h, the CH_2OH was removed and the residue was diluted with 5 mL water, cooled in ice, the precipitate was collected by filtration. Washing the precipitate with 10 ml cold water, the crude material was purified *via* silica gel column chromatography using CH_2Cl_2: MeOH (95:5) as the eluent. It was dried under vacuum to afford the pure product as a brown powder; the yield was about 65 %.

^{1}H NMR (600 MHz, DMSO-*d6*) δ 10.93 (s, 1H), 9.00 (s, 1H), 8.65 (s, 1H), 7.95 (s, 2H), 7.77 (s, 1H), 7.39 (s, 2H), 6.93 (s, 1H), 6.88 (s, 1H).

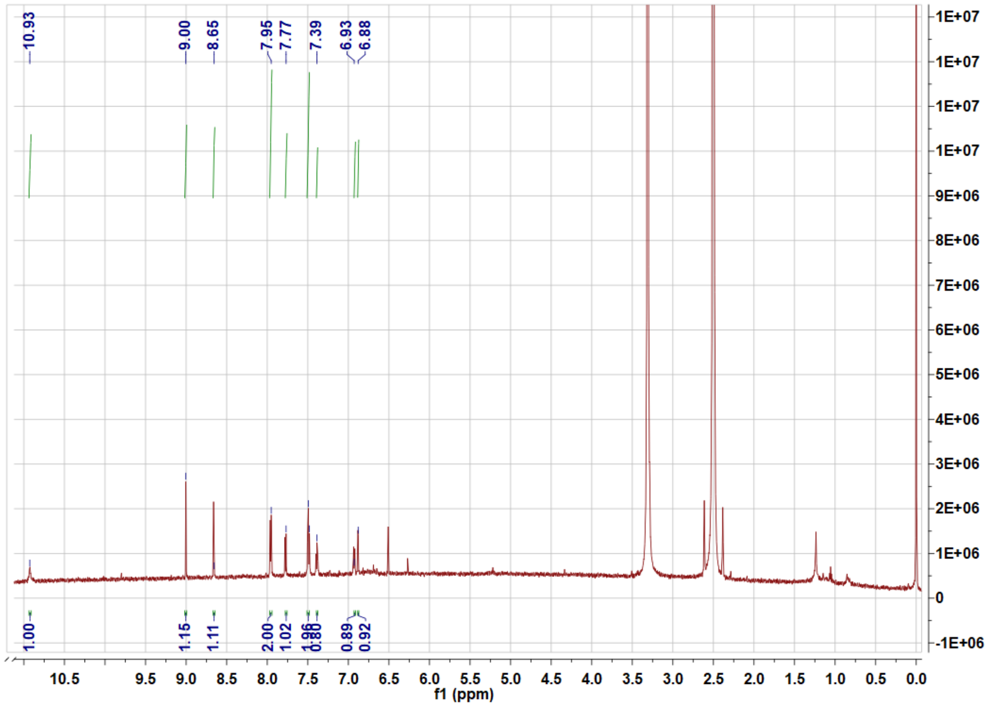

Supplementary Figure 49. ^{1}HNMR spectrum of 7-hydroxy-3-(4-phenyl-1H-[1, 2, 3] triazole-1-yl)-coumarin, **3**. (H_2O δ=3.3; DMSO-*d6* δ=2.5).

5-azidobenzene-1,3-diol (4)

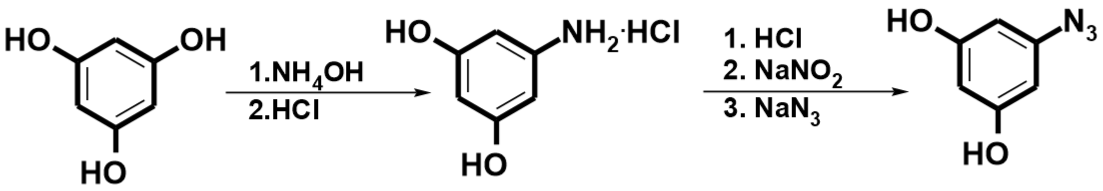

Phloroglucinol (79.5 mmol) and 78 mL $NH_3 \cdot H_2O$ were mixed under N_2 atmosphere. The mixture was stirred for 24h, then the solution was distilled in vacuum to remove the solvent. HCl (6 M) was added under ice bath to product the hydrochloric acid salt. The solvent distilled in vacuum, and then the product was purified through MeOH/CH_2Cl_2 reprecipitation. The obtained yellow precipitation is the precursor of compound (**4**).[14]

^{1}H NMR (600 MHz, CD_3OD) δ6.30 (s, 2H), 6.34 (s, 1H).

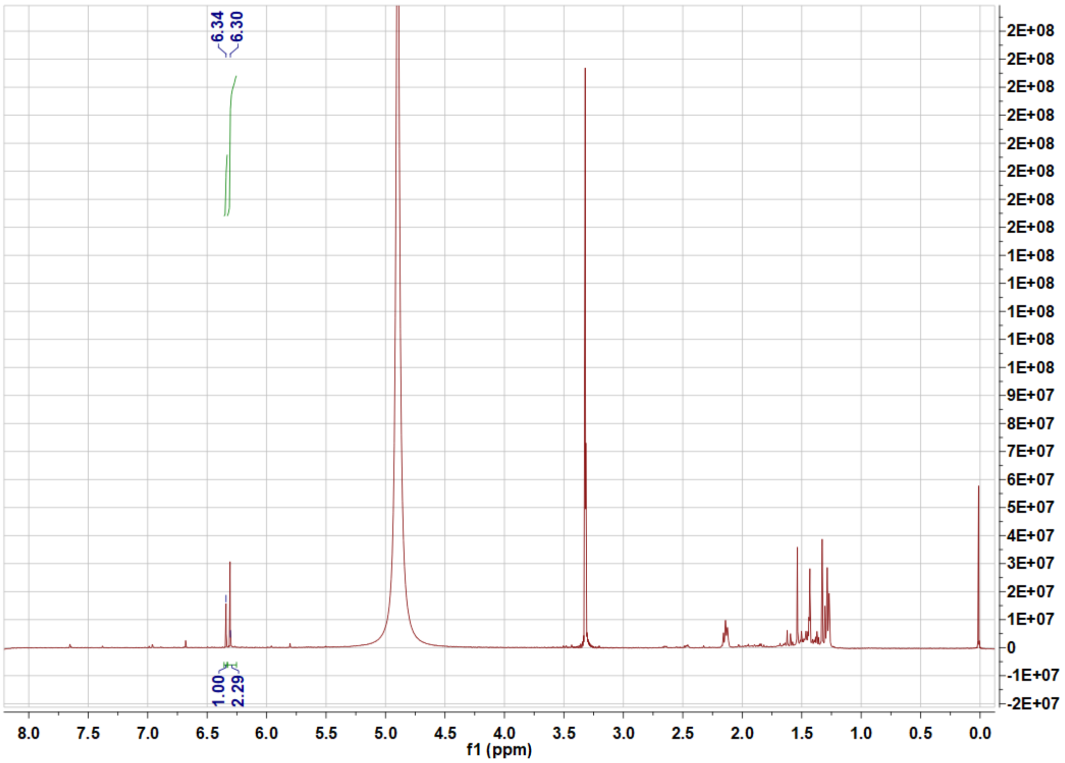

Supplementary Figure 50. ^{1}HNMR spectrum of the precursor of compound **4**. (H_2O δ=3.3).

5 g of 5-Aminoresorcinol hydrochloride was added to a solution of 12.5 mL distilled water and 12.5 mL conc. HCl at 0 °C. $NaNO_2$ (28 mmol) dissolved in 12.5 mL distilled water was added to the above solution slowly (>5 min). After 10 min, NaN_3 (33 mmol) dissolved in 12.5 mL distilled water was added, and the reaction then allowed to stir for additional 40 min at 0 °C. The resulting solution was extracted with EtOAc (3 x 20 mL). Organic layers were combined, washed with brine (1 x 20 mL), dried over anhydrous $MgSO_4$, and concentrated under vacuum. The crude product was chromatographed with Hexane/EtOAc as eluant, to afford the light-yellow crystal, **4**.

^{1}H NMR (600 MHz, d-acetone) δ 8.51 (s, 1H), 6.18 (s, 1H), 6.05 (d, J = 2.1 Hz, 1H).

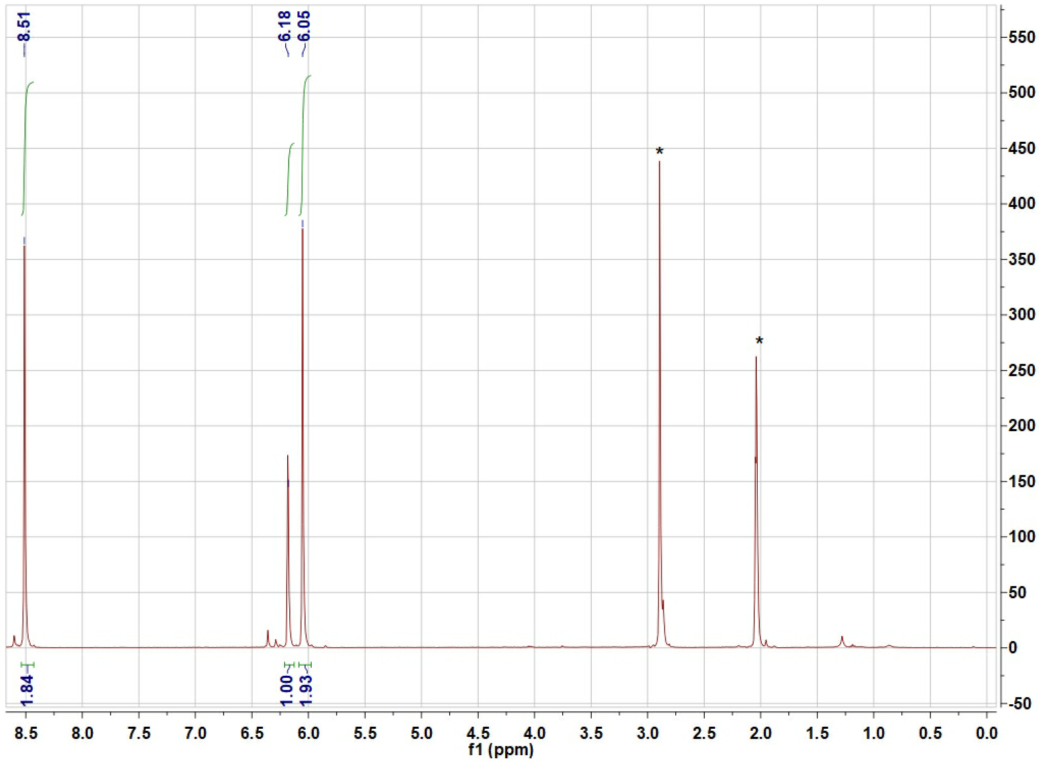

Supplementary Figure 51. ^{1}HNMR spectrum of 5-azidobenzene-1, 3-diol, **4** (H_2O δ=3.3; *d*-acetone δ=2.05).

The precursor of 4-ethynylphenol (5) 4-((trimethylsilyl)ethynyl) phenol

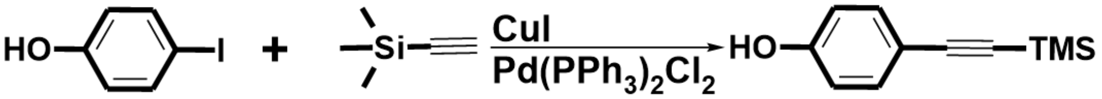

Ethynyltrimethylsilane (6.72 mmol) was added to a solution of 4-iodophenol (4.64 mmol), Pd $(PPh_3)_2Cl_2$ (0.140 mmol) and CuI (0.140 mmol) in 15 mL Et_3N and the mixture was refluxed at 80 °C for 3 h under nitrogen. The solution was then cooled to room temperature, filtered, concentrated in vacuo and the crude product was chromatographed with Hexane/EtOAc as eluant, to obtain the precursor of compound (**5**), trimethylsilyl ethynyl phenol (880 mg, 4.62 mmol, quantitative) as the brown oil.

^{1}H NMR (600 MHz, $CDCl_3$): δ 7.37 (d, J = 8.8 Hz, 2H), 6.76 (d, J = 8.8 Hz, 2H), 4.82 (br, 1H), 0.25 (s, 9H);

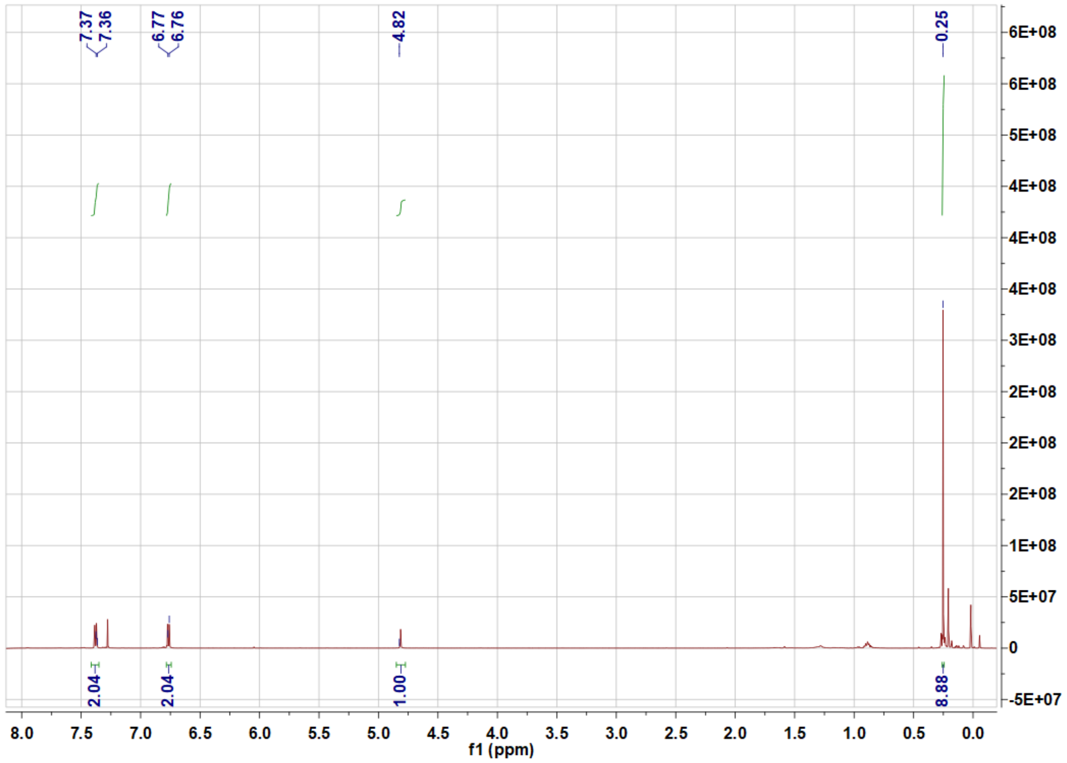

Supplementary Figure 52. ^{1}HNMR spectrum of the precursor of compound (**5**) 4-((trimethylsilyl) ethynyl) phenol. (*d*-$CDCl_3$; δ=7.26).

4-ethynylphenol (5)

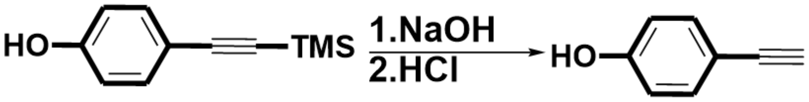

2 mL Aqueous NaOH (5 M) was added to a solution of 4-((trimethylsilyl)ethynyl)phenol (2.74 mmol) in 10 mL MeOH and the reaction solution was stirred for 3 h at room temperature under nitrogen. Then the solution was neutralized with conc. HCl and extracted with DCM (3 x 20 mL). The organic layers were combined, washed with brine (1 x 20 mL), dried over anhydrous $MgSO_4$, and concentrated under vacuum. The crude product was chromatographed with Hexane/EtOAc as eluant, to afford the ethynylphenol (the yield was about 58 %) as a dark red solid.[15]

^{1}H NMR (600 MHz, $CDCl_3$): δ 7.40 (d, J = 8.7 Hz, 2H), 6.79 (d, J = 8.7 Hz, 2H), 5.04 (br, 1H), 2.98 (s, 1H).

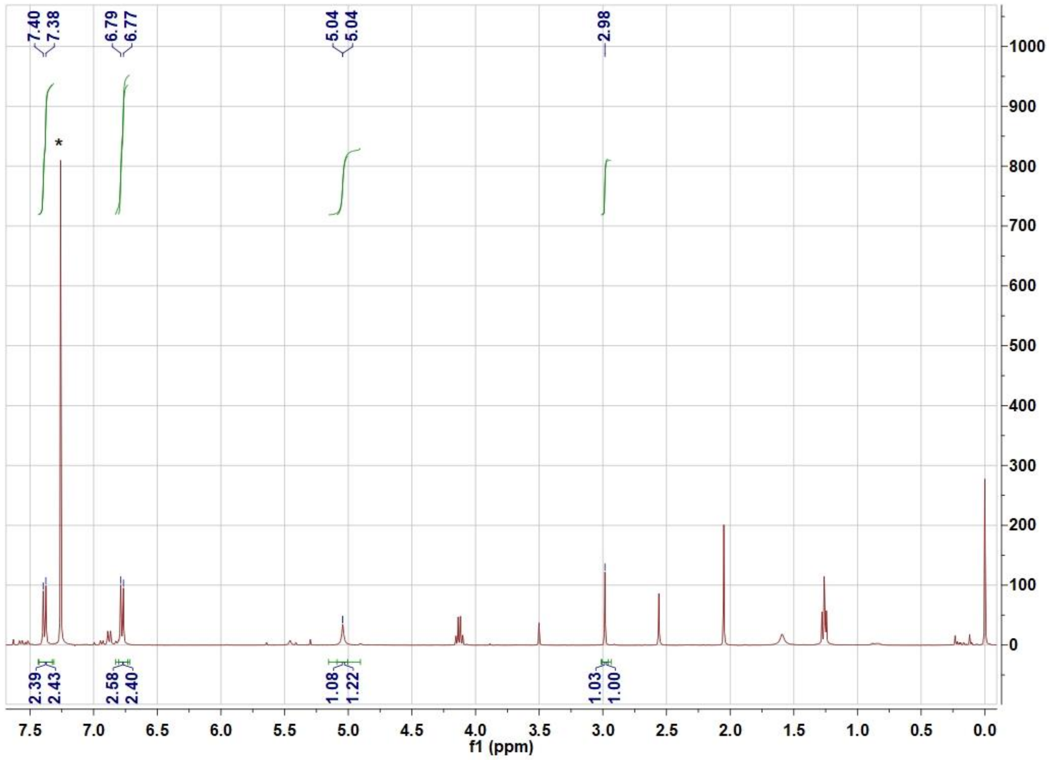

Supplementary Figure 53. ^{1}HNMR spectrum of 4-ethynylphenol, **5**. (*d*-$CDCl_3$; δ=7.26).

5-(4-(4-hydroxyphenyl)-1H-1,2,3-triazol-1-yl) benzene-1,3-diol (6)

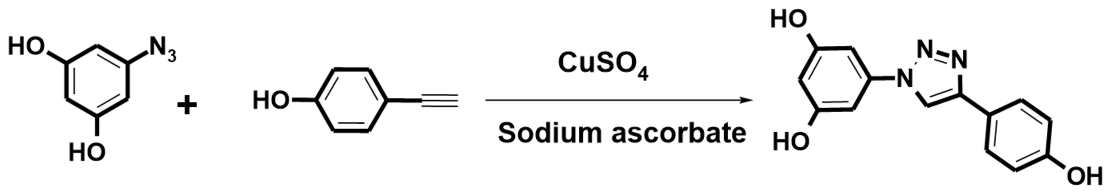

The synthesis was carried out with the following conditions: the overall volume in each test tube was 2 mL (H_2O: t-ButOH=1:1), containing a solution of azide, **4**, (1 eq) and alkyne, **5**, (1 eq). Freshly prepared sodium ascorbate solution (0.5 eq) was added followed by $CuSO_4 \cdot 5H_2O$ (0.05 eq). The reaction was allowed to vigorously stir for 6 h at room temperature. After being diluted with water, the reaction mixture was cooled in ice. The precipitate was collected by filtration, washed with diethyl ether, and dried under vacuum to afford a solid.

^{1}H NMR (600 MHz, DMSO-*d6*) δ 10.17 (s, 1H), 9.64 (s, 1H), 9.57 (s, 1H), 9.51 (s, 1H),7.62 (d, J = 8.5 Hz, 2H), 6.87 (d, J = 8.5 Hz, 2H), 6.84 (d, J = 1.9 Hz, 2H), 6.24 (s, 1H).

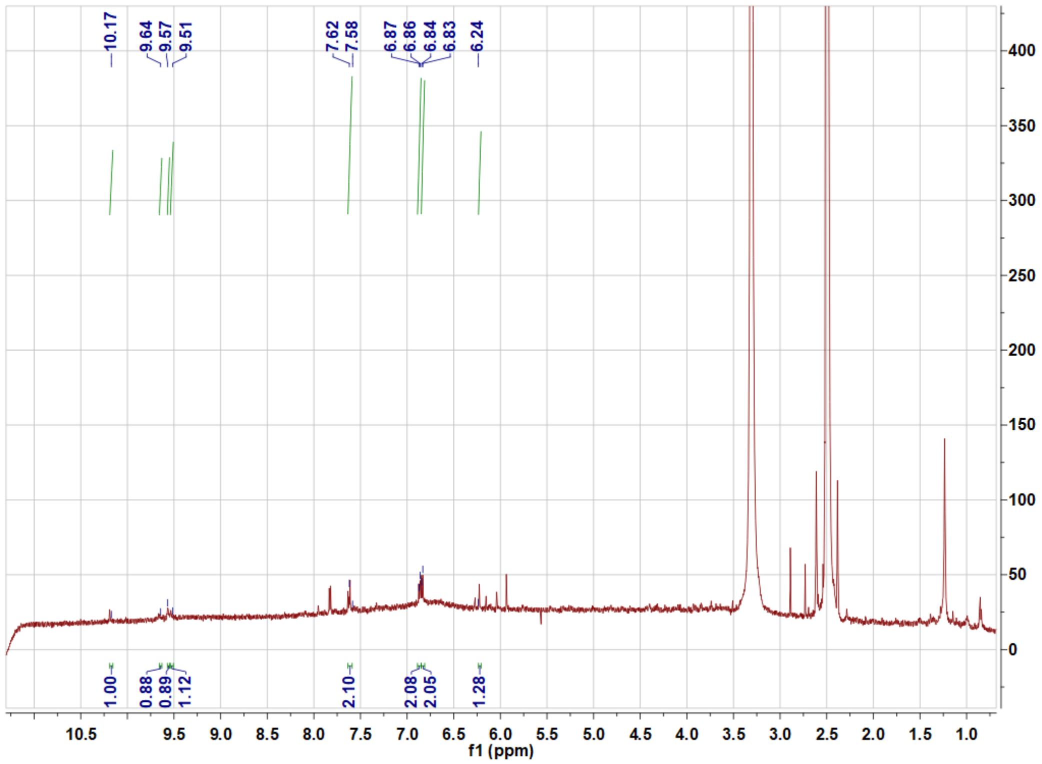

Supplementary Figure 54. ^{1}HNMR spectrum of 5-(4-(4-hydroxyphenyl)-1H-1, 2, 3-triazol-1-yl) benzene-1, 3-diol, **6**.

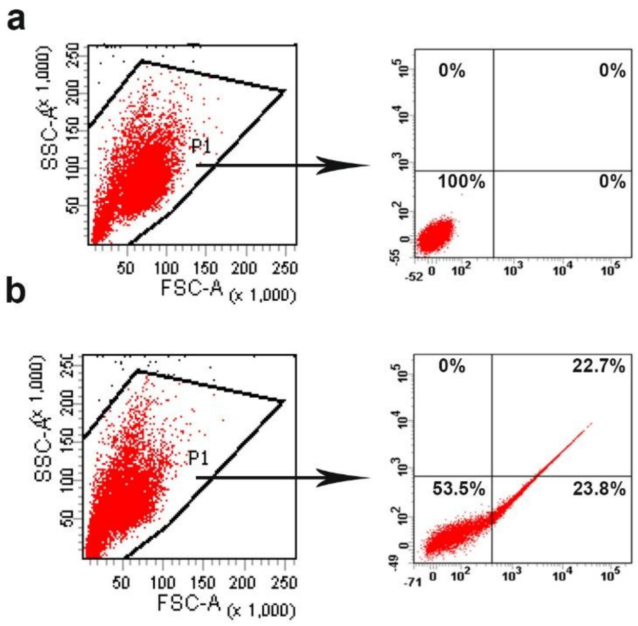

Supplementary Figure 55. The Gating strategy to analyse the apoptotic proportion of a) control group and b) experimental group in tumor cells, respectively.

References

1. Reuter, J. S.; Mathews, D. H., RNAstructure: software for RNA secondary structure prediction and analysis. Bmc Bioinformatics 2010, 11.

2. Phillips, J. C.; Braun, R.; Wang, W.; Gumbart, J.; Tajkhorshid, E.; Villa, E.; Chipot, C.; Skeel, R. D.; Kale, L.; Schulten, K., Scalable molecular dynamics with NAMD. J. Comput. Chem. 2005, 26 (16), 1781-802.

3. Best, R. B.; Zhu, X.; Shim, J.; Lopes, P. E.; Mittal, J.; Feig, M.; Mackerell, A. D., Jr., Optimization of the additive CHARMM all-atom protein force field targeting improved sampling of the backbone phi, psi and side-chain chi(1) and chi(2) dihedral angles. J. Chem. Theory. Comput. 2012, 8 (9), 3257-3273.

4. Klauda, J. B.; Venable, R. M.; Freites, J. A.; O'Connor, J. W.; Tobias, D. J.; Mondragon-Ramirez, C.; Vorobyov, I.; MacKerell, A. D., Jr.; Pastor, R. W., Update of the CHARMM all-atom additive force field for lipids: validation on six lipid types. J. Phys. Chem. B 2010, 114 (23), 7830-43.

5. Martyna, G. J.; Tobias, D. J.; Klein, M. L., Constant-Pressure Molecular-Dynamics Algorithms. J. Chem. Phys. 1994, 101 (5), 4177-4189.

6. Feller, S. E.; Zhang, Y. H.; Pastor, R. W.; Brooks, B. R., Constant-Pressure Molecular-Dynamics Simulation - the Langevin Piston Method. J. Chem. Phys. 1995, 103 (11), 4613-4621.

7. Essmann, U.; Perera, L.; Berkowitz, M. L.; Darden, T.; Lee, H.; Pedersen, L. G., A Smooth Particle Mesh Ewald Method. J Chem Phys 1995, 103 (19), 8577-8593.

8. Trott, O.; Olson, A. J., Software News and Update AutoDock Vina: Improving the Speed and

Accuracy of Docking with a New Scoring Function, Efficient Optimization, and Multithreading. J Comput Chem 2010, 31 (2), 455-461.
9. Laskowski, R. A.; Swindells, M. B., LigPlot+: Multiple Ligand-Protein Interaction Diagrams for Drug Discovery. J Chem Inf Model 2011, 51 (10), 2778-2786.
10. Miller, M. A.; Askevold, B.; Mikula, H.; Kohler, R. H.; Pirovich, D.; Weissleder, R. Nano-Palladium Is a Cellular Catalyst for In Vivo Chemistry. Nat. Commun,2017,8 ,15906.
11. Clavadetscher, J.; Hoffmann, S.; Lilienkampf, A.; Mackay, L.; Yusop, R. M.; Rider, S. A.; Mullins, J. J.; Bradley, M., Copper Catalysis in Living Systems and In Situ Drug Synthesis. Angew. Chem. Int. Ed. 2016, 55, 15662-15666.
12. Yang, R.; Zou, S.; Zhang, X.; Mei, Q., Simultaneous Determination of Polydatin and Its Metabolite in Plasma by Liquid Chromatography/Tandem Mass Spectrometry. Chin. J. Anal. Chem. 2007, 9, 1309-1313.
13. Sivakumar, K.; Xie, F.; Cash, B. M.; Long, S.; Barnhill, H. N.; Wang, Q. A Fluorogenic 1, 3-Dipolar Cycloaddition Reaction of 3-Azidocoumarins and Acetylenes. Org. Lett. 2004, 6, 4603-4606.
14. Andrus, M.B.; Liu, J.; Meredith, E.L.; Nartey, E., Synthesis of Resveratrol Using a Direct Decarbonylative Heck Approach from Resorcylic Acid. Tetrahedron Lett. 2003, 44, 4819-4822.
15. Hudson, S. A.; McLean, K. J.; Surade, S.; Yang, Y. Q.; Leys, D.; Ciulli, A.; Munro, A. W.; and Abell, C, Application of Fragment Screening and Merging to the Discovery of Inhibitors of the Mycobacterium Tuberculosis Cytochrome P450 CYP121. Angew. Chem. Int. Ed. 2012, 51, 9311-9316.
