# Supplementary material for: DNA-based platform for efficient and precisely targeted bioorthogonal catalysis in living systems
Source: Nat Commun. 2022 Mar 18;13:1459. doi: 10.1038/s41467-022-29167-x (PMC8933418; doi:10.1038/s41467-022-29167-x)
Supplement: Supplementary file 2 — Reporting Summary [file 41467_2022_29167_MOESM2_ESM.pdf]

Corresponding author(s): Guangming Li,Fang Pu, Jinsong Ren and Xiaogang Qu

Last updated by author(s): Dec 31, 2021

Reporting Summary

Nature Portfolio wishes to improve the reproducibility of the work that we publish. This form provides structure for consistency and transparency in reporting. For further information on Nature Portfolio policies, see our Editorial Policies and the Editorial Policy Checklist.

Statistics

For all statistical analyses, confirm that the following items are present in the figure legend, table legend, main text, or Methods section.

n/a	Confirmed	
☐	☒	The exact sample size (n) for each experimental group/condition, given as a discrete number and unit of measurement
☐	☒	A statement on whether measurements were taken from distinct samples or whether the same sample was measured repeatedly
☐	☒	The statistical test(s) used AND whether they are one- or two-sided<br>*Only common tests should be described solely by name; describe more complex techniques in the Methods section.*
☐	☒	A description of all covariates tested
☒	☐	A description of any assumptions or corrections, such as tests of normality and adjustment for multiple comparisons
☐	☒	A full description of the statistical parameters including central tendency (e.g. means) or other basic estimates (e.g. regression coefficient) AND variation (e.g. standard deviation) or associated estimates of uncertainty (e.g. confidence intervals)
☐	☒	For null hypothesis testing, the test statistic (e.g. F, t, r) with confidence intervals, effect sizes, degrees of freedom and P value noted<br>*Give P values as exact values whenever suitable.*
☒	☐	For Bayesian analysis, information on the choice of priors and Markov chain Monte Carlo settings
☒	☐	For hierarchical and complex designs, identification of the appropriate level for tests and full reporting of outcomes
☒	☐	Estimates of effect sizes (e.g. Cohen's d, Pearson's r), indicating how they were calculated

Our web collection on statistics for biologists contains articles on many of the points above.

Software and code

Policy information about availability of computer code

Data collection	The zeta-potential of the nanoparticles was measured in a Zetasizer 3000HS analyzer.Transmission electron microscopic (TEM) images were captured by a FEI TECNAI G2 20 high-resolution transmission electron microscope operating at 200 kV. 1H NMR spectrum was recorded on a Bruker-600 MHz NMR instrument. Fluorescence spectra were detected by JASCO F-6000 fluorescence spectrometer with a Peltier temperature control accessory. ICP-MS measurements were performed on a TheroScientific Xseries Ⅱ inductively coupled plasma mass spectrometer. The X-ray photoelectron Spectroscopy (XPS) spectra were analyzed by Thermo Fisher Scientific ESCALAB 250Xi Spectrometer Electron Spectroscopy (America). The High Performance Liquid Chromatography (HPLC) was measured by Ultimate 3000. The Liquid Chromatography Mass Spectra (LCMS) was obtained using Quattro Premier XE (USA). The confocal laser scanning microscopy (CLSM) characterization was acquired by a (Nikon Eclipse Ni-E, Japan) top-of-the-line motorized upright. The flow cytometry data were obtained by BD LSRFortessa™ Cell Analyzer. The docking poses of ligands were determined by AutoDock Vina 1.1.28.
Data analysis	Microsoft Office Excel 2018. and Origin 2020. were used for statistical analysis. FlowJo_V10. was used for analysis of Flow Cytometer.

For manuscripts utilizing custom algorithms or software that are central to the research but not yet described in published literature, software must be made available to editors and reviewers. We strongly encourage code deposition in a community repository (e.g. GitHub). See the Nature Portfolio guidelines for submitting code & software for further information.

Data

Policy information about availability of data

All manuscripts must include a data availability statement. This statement should provide the following information, where applicable:

- Accession codes, unique identifiers, or web links for publicly available datasets
- A description of any restrictions on data availability
- For clinical datasets or third party data, please ensure that the statement adheres to our policy

All experiment data supporting the findings of this study are available within the article, Supplementary Information, and Source Data. Source data are provided with this paper.

Field-specific reporting

Please select the one below that is the best fit for your research. If you are not sure, read the appropriate sections before making your selection.

[x] Life sciences   [ ] Behavioural & social sciences   [ ] Ecological, evolutionary & environmental sciences

For a reference copy of the document with all sections, see nature.com/documents/nr-reporting-summary-flat.pdf

Life sciences study design

All studies must disclose on these points even when the disclosure is negative.

Sample size	All data were obtained from a minimum of three independent experiments and were presented as the mean ± standard deviation (SD). Statistical evaluation was performed using two-tailed Student's t test analysis.
Data exclusions	No data were excluded from the analyses.
Replication	All experiments were conducted at least two times and could be reliably reproduced.
Randomization	The samples were divided into different groups randomly in all experiments.
Blinding	Formal blinding was used for H&E staining of tumor tissues.

Reporting for specific materials, systems and methods

We require information from authors about some types of materials, experimental systems and methods used in many studies. Here, indicate whether each material, system or method listed is relevant to your study. If you are not sure if a list item applies to your research, read the appropriate section before selecting a response.

Materials & experimental systems

n/a	Involved in the study	
[x]	[ ]	Antibodies
[ ]	[x]	Eukaryotic cell lines
[x]	[ ]	Palaeontology and archaeology
[ ]	[x]	Animals and other organisms
[x]	[ ]	Human research participants
[x]	[ ]	Clinical data
[x]	[ ]	Dual use research of concern

Methods

n/a	Involved in the study	
[x]	[ ]	ChIP-seq
[ ]	[x]	Flow cytometry
[x]	[ ]	MRI-based neuroimaging

Eukaryotic cell lines

Policy information about cell lines

Cell line source(s)	Mouse NIH-3T3 embryo fibroblast cells, murine RAW 264.7 macrophage cell, human HepG2 hepatocellular carcinoma cells, human A549 lung cancer cell, human MDA-MB-231 breast cancer cell, human MCF-7 breast cancer cells,human HeLa cervical cancer cells and human HEK 293 embryonic kidney cells were purchased from China Center for Type Culture Collection.
Authentication	Cell lines used were not further authenticated.
Mycoplasma contamination	Cell lines were not tested for mycoplasma contamination.
Commonly misidentified lines (See ICLAC register)	None of these cell lines were used.

Animals and other organisms

Policy information about studies involving animals; ARRIVE guidelines recommended for reporting animal research

Laboratory animals	Six-week-old female Balb/c nude mice (14 - 16 g) were used in the experiment. All mice were housed in a specific pathogen-free environment at 26±1 ℃ and 50±5% humidity, with a 12h light-dark cycle.
Wild animals	The study did not involve wild animals.
Field-collected samples	The study did not involve samples collected from the field.
Ethics oversight	All animal handling procedures were in accordance with the guidelines of the Animal Ethics Committee of Jilin University for Animal Experiments.

Note that full information on the approval of the study protocol must also be provided in the manuscript.

Flow Cytometry

Plots

Confirm that:

- [x] The axis labels state the marker and fluorochrome used (e.g. CD4-FITC).
- [x] The axis scales are clearly visible. Include numbers along axes only for bottom left plot of group (a 'group' is an analysis of identical markers).
- [x] All plots are contour plots with outliers or pseudocolor plots.
- [x] A numerical value for number of cells or percentage (with statistics) is provided.

Methodology

Sample preparation	MCF-7, A549, NIH-3T3, RAW, MDA-MB-231, HepG2,HeLa, HEK293 cells were plated in 6-wellplates. Nanocatalysts(5 μM) were added and incubated with the cells for 4 h, followed by washing with PBS for 3 times.Next, azide coumarin (10 μM) and alkyne (10 μM) were added and incubated with the cells for 12 h. After being washed with PBS for 2 times, the cells were harvested by trypsin and resuspended in PBS. The intracellular fluorescence of product 3 was analyzed using flow cytometry (450/50).For nanocatalyst-mediated synthesis of resveratrol analogue inside living cells (MCF-7, A549, NIH-3T3, RAW, MDA-MB-231, HepG2 cells) were plated in 6-well plates. , the experimental conditions were the same as above, except for replacing azide coumarin and alkyne with prodrugs. The apoptosis of these cells induced by resveratrol analogue was analyzed by double staining with Annexin VFITC and propidium iodide using commercial apoptosis detection kit.
Instrument	BD LSRFortessa™ Cell Analyzer.
Software	BD LSRFortessa™ Cell Analyzer Software and FlowJo_V10.
Cell population abundance	The absolute cells around 10000 were analyzed for each group.
Gating strategy	Initial cell populations were gated for a live population using FSC and SSC plot of cell only sample. The gate was set to remove cell debris and dead cells.

- [x] Tick this box to confirm that a figure exemplifying the gating strategy is provided in the Supplementary Information.
